# Small molecule inhibitors targeting the cancers

**DOI:** 10.1002/mco2.181

**Published:** 2022-10-13

**Authors:** Gui‐Hong Liu, Tao Chen, Xin Zhang, Xue‐Lei Ma, Hua‐Shan Shi

**Affiliations:** ^1^ Department of Biotherapy State Key Laboratory of Biotherapy Cancer Center, West China Hospital Sichuan University Chengdu China; ^2^ Department of Cardiology The First Affiliated Hospital of China Medical University Shenyang Liaoning China

**Keywords:** combination therapy, multikinase molecule inhibitors, resistance, small molecule inhibitors, small molecule kinase inhibitors

## Abstract

Compared with traditional therapies, targeted therapy has merits in selectivity, efficacy, and tolerability. Small molecule inhibitors are one of the primary targeted therapies for cancer. Due to their advantages in a wide range of targets, convenient medication, and the ability to penetrate into the central nervous system, many efforts have been devoted to developing more small molecule inhibitors. To date, 88 small molecule inhibitors have been approved by the United States Food and Drug Administration to treat cancers. Despite remarkable progress, small molecule inhibitors in cancer treatment still face many obstacles, such as low response rate, short duration of response, toxicity, biomarkers, and resistance. To better promote the development of small molecule inhibitors targeting cancers, we comprehensively reviewed small molecule inhibitors involved in all the approved agents and pivotal drug candidates in clinical trials arranged by the signaling pathways and the classification of small molecule inhibitors. We discussed lessons learned from the development of these agents, the proper strategies to overcome resistance arising from different mechanisms, and combination therapies concerned with small molecule inhibitors. Through our review, we hoped to provide insights and perspectives for the research and development of small molecule inhibitors in cancer treatment.

## Graphical abstract

Small molecule inhibitors have been permitted to be used in post‐line therapy, first‐line treatment, and adjuvant therapy. Compared with post‐line and first‐line treatment, attempts at adjuvant therapy are just beginning. Several factors should be considered in the adjuvant setting, including other standard treatments, subsequent therapy, financial benefit ratio, and treatment‐related side effects.

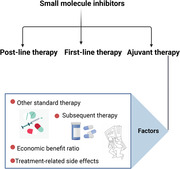

## 1 INTRODUCTION

Tumors are complex, and there are some ways to treat them. Cancer chemotherapy and radiation therapy have some selectivity for tumor cells because of their increased proliferation rate.^1^ With the advent of modern cell biology since the 1980s, lots of molecular drivers of cancer have been obtained and targeted cancer therapy has become dominant in novel drug development.^2^

Antibody therapies and small molecule inhibitors are the two main methods for targeted cancer treatment.^3^ The mechanism of small molecule inhibitors is to inhibit the target proteins’ function by binding to the “pocket” on their surface. Small molecule inhibitors can bind a wider range of extracellular and intracellular targets compared with antibodies due to their smaller size. Besides, most small molecule inhibitors can be taken orally, while antibodies are administered subcutaneously or intravenously. What is more, some of small molecule inhibitors can penetrate the blood–brain barrier to control intracranial lesions.^1^, ^2^, ^3^, ^4^

The targets of these drugs cover a large scope. Most small molecule inhibitors belong to protein kinase inhibitors.^5^, ^6^, ^7^ In addition, drugs involved in deoxyribonucleic acid (DNA) repair, epigenetics, apoptosis, tumor metabolism, and beyond are also being discovered.^8^, ^9^, ^10^, ^11^ Surprisingly, targets considered untargetable or difficult to target in the past, such as RAS, have also been approved recently.^12^, ^13^ It is undeniable that small molecule inhibitors still encounter many challenges such as low response rate and drug resistance.

In our review, we first describe the classification of small molecule inhibitors, including selective small molecule kinase inhibitors, selective small molecule nonkinase inhibitors, and multikinase small molecule inhibitors. Then, we discuss the small molecule inhibitors according to signaling pathways and the classification of small molecule inhibitors. In each part of specific inhibitors, we first introduce this target and the pathways involved in the target, as well as its significance in tumors. Followed by discussing United States Food and Drug Administration (US FDA)‐approved small molecule inhibitors. We subsequently introduce small molecule inhibitors in clinical trials summarized from clinical trials website. At the end of our review, we also discuss the main issues and future directions concerned with small molecule inhibitors.

## 2 CLASSIFICATION OF SMALL MOLECULE INHIBITORS

**FIGURE 1:**
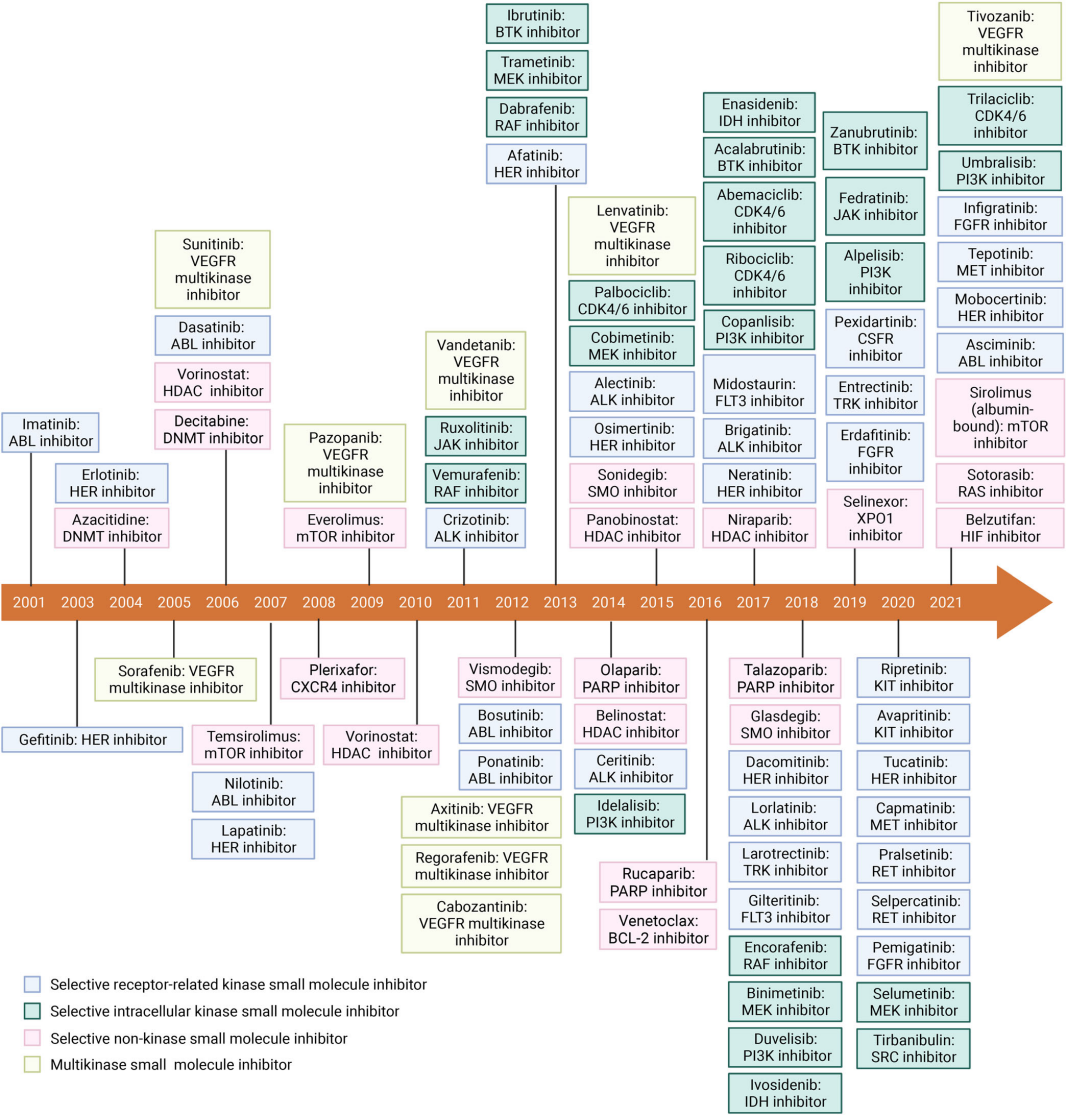
Timeline for the US FDA‐approved small molecule inhibitors targeting the cancers. Figure created with BioRender.com

To our knowledge, there are 88 small molecule inhibitors approved by the US FDA for oncology indications by August 2022^5^, ^6^, ^7^, ^8^, ^9^, ^10^, ^11^ (Figure 1). According to target selectivity, small molecule inhibitors can be divided into selective small molecule inhibitors and multikinase small molecule inhibitors. According to whether the substrate is a protein kinase, selective small molecule inhibitors are further divided into selective small molecule kinase inhibitors and selective small molecule nonkinase inhibitors.^10^

Selective small molecule inhibitors usually bind to a single target and inhibit the target‐related cell signaling. A subset of cancers strongly relies on a few dysfunctions related to growth, survival, apoptosis, differentiation, cancer metabolism, and even immune modulation.^14^, ^15^ Selective small inhibitors can antagonize the critical target to inhibit its unusual function or reverse its regular action, correspondingly, to treat tumors. Patients commonly need strict screening for the presence or absence of specific gene alteration detected from solid tumor tissue or circulating tumor cells in the blood or other body fluid when treated by selective small molecule inhibitors.^16^ Under this condition, selective small molecule inhibitors can effectively target tumors and avoid side effects brought by off‐target inhibition.^17^, ^18^

**FIGURE 2:**
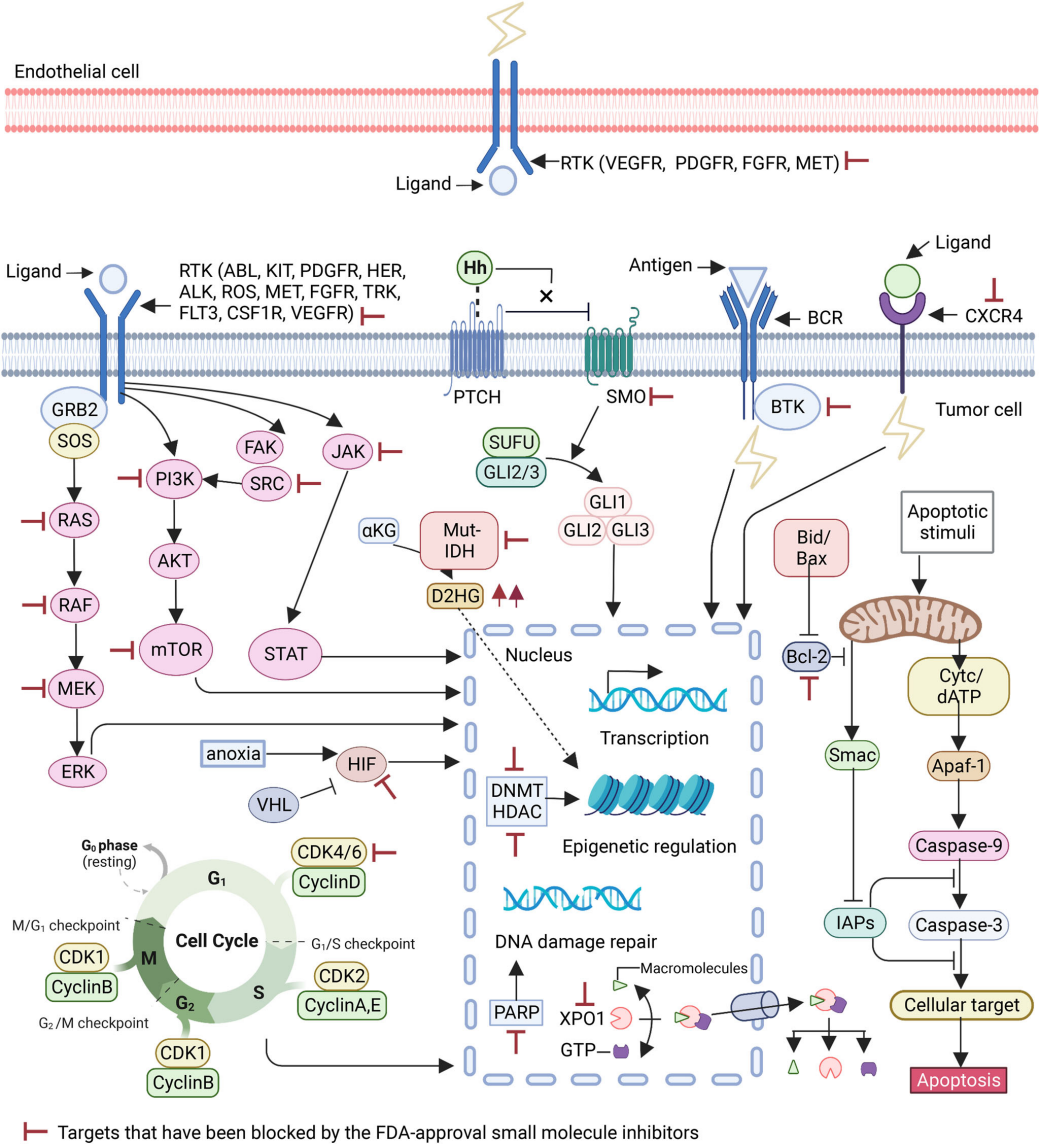
The mechanisms of the US FDA‐approved small molecule inhibitors targeting the cancers. The cell surface receptors and signaling pathways, DNA transcription, DNA damage repair, epigenetic modifications, nuclear transport, blood vessels, and apoptosis are involved in the targets of the US FDA‐approved small molecule inhibitors. Figure created with BioRender.com

Selective small molecule inhibitors are further divided into selective small molecule kinase inhibitors and selective small molecule nonkinase inhibitors.^10^ Protein kinase inhibitors are the main category of small molecule inhibitors and the criterion for a protein kinase inhibitors to be a multikinase or selective small molecule kinase inhibitor is the number of kinases whose values of IC50 of inhibitory activity are below 10 nM.^19^ Multikinase small molecule inhibitors exert anticancer activity by repressing multiple protein kinases in the tumor. Multikinase inhibitors do not require precise detection but rely on histology.^20^, ^21^ Most approved drugs are multikinase inhibitors of the vascular endothelial growth factor receptors (VEGFR) owning antiangiogenic and antiproliferative effects. Selective small molecule kinase inhibitors account for most selective small molecule inhibitors.^10^ The selective small molecule kinase inhibitors contains receptor‐related kinase inhibitors, kinase inhibitors targeted intracellular signaling pathways, and inhibitors targeting other cytoplasmic kinases.^22^ Beyond the kinome, many molecular targets with proven roles in cancer have gradually developed into selective small molecule nonkinase inhibitors. The molecular targets involved in the nucleus, whose mechanisms are gene transcription, DNA repair, epigenetic modification, and nuclear protein exportation. Some of receptor and intracellular signaling inhibitors and agents involved in triggering apoptosis are also fall into this category.^23^ The details of mechanisms will be discussed below and have been depicted in Figure 2.

Both selective small molecule kinase inhibitors and multikinase small molecule inhibitors belong to protein kinase inhibitors.^24^ 560 protein kinases in the human kinome are divided into 500 eukaryotic protein kinases (ePKs) and 60 atypical protein kinases (aPKs). Tyrosine kinases and threonine/serine kinases are two essential classes of eight categories of ePKs, while lipid kinases belong to aPKs.^25^ Protein kinases bind adenosine triphosphate (ATP) through their ATP‐binding pocket and then transfer the γ phosphate group at the end of ATP to the substrate, thereby activating the substrate and the signal transduction pathway.^26^ Reversible protein phosphorylation mediated by kinases and phosphatases has a crucial role in regulating cellular functions.^27^ Deregulation of kinases’ function owing to their mutations, translocations, or overexpression in cancer offers an opportunity for small molecule kinase inhibitors, which can block the binding of ATP to protein kinases.^28^

The highly dynamic nature of protein kinases allows for the design of inhibitors that recognize the active or multiple inactive conformations. Correspondingly, protein kinase inhibitors are classified into six types (type I–VI) based on their biochemical mechanisms of action.^29^ Type I inhibitors interact directly with the ATP binding site and target the kinase active state, which is characterized by the DFG‐in conformation. Besides, αC is in their active “in” position and the G‐loop is not in an active conformation.^30^ Type II inhibitors also bind to the ATP binding site but with DFG‐out catalytically inactive conformation. The state of αC and G‐loop of type II inhibitors maintains the same as type I inhibitors. The binding modes of type I1/2 inhibitors are between those of canonical type I and type II inhibitors.^31^ Type I1/2 inhibitors definitely disrupt the kinase's R‐​spine, and the DFG motif remains in a DFG‐in position.^32^ Both type III and IV inhibitors belong to allosteric inhibitors, which bind to the hydrophobic pocket of kinase and are more selective than ATP‐competitive inhibitors. The difference between type III and IV inhibitors is the distance between the hydrophobic pocket and the ATP‐binding site. Type III inhibitors’ hydrophobic binding pocket is adjacent to the ATP‐binding site, while type IV inhibitors bind away from the ATP binding pocket.^33^ Type V inhibitors, also known as bi‐substrate inhibitors, interact with both the allosteric and ATP binding pockets.^34^ The above classes of inhibitors interact reversibly, while type VI inhibitors form an irreversible covalent bond with cysteine residues in and around the ATP‐binding site of the kinase. These covalent approaches make type VI inhibitors more potent and more specific.^35^

In our review, we will introduce them in the order of selective small molecule kinase inhibitors, selective small molecule nonkinase inhibitors, and multikinase small molecule inhibitors.

## 3 SELECTIVE SMALL MOLECULE KINASE INHIBITORS

The number of selective small molecule kinase inhibitors is the largest in the US FDA‐approved small molecule inhibitors. This part will initially describe the first approved ABL kinase inhibitor, followed by receptor‐related kinase inhibitors like human epidermal growth factor recepter (HER), anaplastic lymphoma kinase (ALK), and fibroblast growth factor receptor (FGFR) kinase inhibitors. Then, attention will be given to kinase inhibitors targeted intracellular signaling pathways involving RAF/MEK/ERK, PI3K/AKT/mTOR, and JAK/STAT3 signaling. Finally, inhibitors targeting other cytoplasmic kinases will be introduced. The selective small molecule kinase inhibitors approved by the US FDA are summarized in Table 1 and the pivotal candidates of small molecule kinase inhibitors are listed in Table S1.

**TABLE 1:** Summary of approved selective small molecule kinase inhibitors

Class	Drug name	Company	First approval	Target	Protein substrate	Administration pathway	Indications
ABL	Imatinib (Gleevec)	Novartis	2001	BCR–ABL, PDGFR, SCF, KIT	Tyrosine	Oral	Ph‐positive CML, Ph‐positive ALL, PDGFR rearrangements MDS/MPD, ASM, HES, CEL, DFSP, KIT‐positive GIST
ABL	Dasatinib (Sprycel)	Bristol‐Myers Squibb	2006	BCR–ABL, SRC family (SRC, LCK, YES, FYN), and KIT, EPHA2, PDGFRβ	Tyrosine	Oral	Ph‐positive CML, Ph‐positive ALL,
ABL	Nilotinib (Tasigna)	Novartis	2007	BCR–ABL, PDGFRB, KIT	Tyrosine	Oral	Ph‐positive CML
ABL	Bosutinib (Bosulif)	Wyeth Inc	2012	BCR–ABL, SRC‐family (SRC, LYN, and HCK)	Tyrosine	Oral	Ph‐positive CML
ABL	Ponatinib (Iclusig)	Ariad	2012	BCR–ABL, BCR–ABL (T315I), VEGFR, PDGFR, FGFR, EPH receptors, SRC families of kinases, KIT, RET, TIE2, FLT3	Tyrosine	Oral	Ph‐positive CML and Ph‐positive ALL resistant/intolerant to therapy, T315I‐positive CML, T315I‐positive/Ph‐positive ALL
ABL	Asciminib (Scemblix)	Novartis	2021	BCR–ABL, BCR–ABL (T315I)	Tyrosine	Oral	Ph‐positive CML‐CP resistant to therapy, T315I‐positive CML
KIT	Ripretinib (Quinlock)	Deciphera	2020	KIT, PDGFRA, PDGFRA mutations, PDGFRB, TIE2, VEGFR2, BRAF	Tyrosine	Oral	GIST
KIT	Avapritinib (Ayvakit)	Blueprint Medicines	2020	KIT, KIT D816V, KIT exon 11, 11/17, and 17 mutants, PDGFRA and PDGFRA D842 mutants, PDGFRB, and CSFR1	Tyrosine	Oral	PDGFRA exon 18 mutation (including D842V) positive GIST, advanced systemic mastocytosis
HER	Gefitinib (Iressa)	AstraZeneca	2003	EGFR and HER family	Tyrosine	Oral	NSCLC
HER	Erlotinib (Tarceva)	OSI	2004	EGFR and HER family	Tyrosine	Oral	NSCLC with EGFR 19del or L858R, pancreatic cancer
HER	Afatinib (Gilotrif)	Boehringer Ingelheim	2013	EGFR and HER family	Tyrosine	Oral	NSCLC with nonresistant EGFR mutations, squamous NSCLC
HER	Osimertinib (Tagrisso)	AstraZeneca	2015	EGFR and HER family	Tyrosine	Oral	NSCLC with EGFR 19del or L858R, NSCLC with T790M positive
HER	Dacomitinib (Vizimpro)	Pfizer	2018	EGFR and HER family	Tyrosine	Oral	NSCLC with EGFR 19del or L858R
HER	Mobocertinib (Exkivity)	Takeda Pharmaceuticals	2021	EGFR and HER family	Tyrosine	Oral	NSCLC with EGFR 20 exon insertion
HER	Lapatinib (Tykerb)	SmithKline Beecham	2007	EGFR and HER family	Tyrosine	Oral	HER2‐positive breast cancer
HER	Neratinib (Nerlynx)	Puma Biotechnology	2017	EGFR and HER family	Tyrosine	Oral	HER2‐positive breast cancer
HER	Tucatinib (Tukysa)	Seattle Genetics	2020	EGFR and HER family	Tyrosine	Oral	HER2‐positive breast cancer
ALK	Crizotinib (Xalkori)	PF Prism CV	2011	ALK, HGFR, c‐Met, ROS1, RON	Tyrosine	Oral	ALK‐ or ROS1‐positive NSCLC, ALK‐positive anaplastic large cell lymphoma
ALK	Ceritinib (Zykadia)	Novartis	2014	ALK, IGF‐1R, InsR, ROS1	Tyrosine	Oral	ALK‐positive NSCLC
ALK	Alectinib (Alecensa)	Roche	2015	ALK, RET	Tyrosine	Oral	ALK‐positive NSCLC
ALK	Brigatinib (Alunbrig)	ARIAD	2017	ALK, ROS1, IGF‐1R, FLT‐3, EGFR deletion and point mutations	Tyrosine	Oral	ALK‐positive NSCLC
ALK	Lorlatinib (Lorviqua)	Pfizer	2018	ALK, ROS1, TYK1, FER, FPS, TRKA, TRKB, TRKC, FAK, FAK2, ACK	Tyrosine	Oral	ALK‐positive NSCLC
MET	Capmatinib (Tabrecta)	Novartis	2020	MET, MET exon 14 skipping	Tyrosine	Oral	NSCLC with MET exon 14 skipping
MET	Tepotinib (Tepmetko)	Merck	2021	MET, MET exon 14 skipping	Tyrosine	Oral	NSCLC with MET exon 14 skipping
RET	Pralsetinib (Gavreto)	Blueprint Medicines	2020	wild‐type RET, oncogenic RET fusions (CCDC6‐RET), RET mutations (RET V804L, RET V804M and RET M918T)	Tyrosine	Oral	RET fusion‐positive NSCLC, RET mutant MTC, RET fusion‐positive thyroid cancer
RET	Selpercatinib (Retevmo)	Eli Lilly	2020	wild‐type RET, multiple mutated RET isoforms	Tyrosine	Oral	RET fusion‐positive NSCLC, RET mutant MTC, RET fusion‐positive thyroid cancer
FGFR	Erdafitinib (Balversa)	Janssen	2019	FGFR1, FGFR2, FGFR3, FGFR4, RET, CSF1R, PDGFRA, PDGFRB, FLT4, KIT, VEGFR2	Tyrosine	Oral	Urothelial carcinoma with FGFR3 or FGFR2 genetic alterations
FGFR	Pemigatinib (Pemazyre)	Incyte	2020	FGFR1, FGFR2, FGFR3	Tyrosine	Oral	Cholangiocarcinoma with FGFR2 fusion or other rearrangement
FGFR	Infigratinib (Truseltiq)	Helsinn Hlthcare	2021	FGFR1, FGFR2, FGFR3, FGFR4	Tyrosine	Oral	Cholangiocarcinoma with FGFR2 fusion or other rearrangement
TRK	Larotrectinib (Vitrakvi)	Loxo Oncology	2018	NTRK1, NTRK2, NTRK3	Tyrosine	Oral	NTRK fusion‐positive tumours
TRK	Entrectinib (Rozlytrek)	Genentech	2019	NTRK1, NTRK2, NTRK3, ROS1, ALK, JAK2, TNK2	Tyrosine	Oral	NTRK fusion‐positive tumours, ROS1 positive NSCLC
FLT3	Midostaurin (Rydapt)	Novartis	2017	FLT3	Tyrosine	Oral	FLT mutant AML, ASM, SM‐AHN, MCL
FLT3	Gilteritinib (Xospata)	Astellas	2018	FLT3	Tyrosine	Oral	FLT mutant AML
CSF1R	Pexidartinib (Turalio)	Daiichi Sankyo	2019	CSF1R, KIT, FLT3 with ITD mutation	Tyrosine	Oral	Tenosynovial giant cell tumor
RAF	Vemurafenib (Zelboraf)	Hoffmann La Roche	2011	mutated forms of BRAF, wild‐type BRAFCRAF, ARAF, SRMS, ACK1, MAP4K5, FGR	Serine‐threonine	Oral	Melanoma with BRAF V600E
RAF	Dabrafenib (Tafinlar)	GSK	2013	BRAF V600E, BRAF V600K, and BRAF V600D, wild‐type BRAF, CRAF, SIK1, NEK11, LIMK1	Serine‐threonine	Oral	Melanoma with BRAF V600E, in combination with trametinib: melanoma with BRAF V600E or V600K, NSCLC with BRAF V600E, ATC with BRAF V600E, solid tumors with BRAF V600E
RAF	Encorafenib (Braftovi)	Array BioPharma	2018	BRAF V600E, wild‐type BRAF, CRAF, JNK1, JNK2, JNK3, LIMK1, LIMK2, MEK4, STK36	Serine‐threonine	Oral	In combination with binimetinib: melanoma with BRAF V600E or V600K, in combination with cetuximab: CRC with BRAF V600E
MEK	Trametinib (Mekinist)	GSK	2013	MEK1, MEK2	Serine‐threonine	Oral	Melanoma with BRAF V600E or V600K, in combination with dabrafenib: melanoma with BRAF V600E or V600K, NSCLC with BRAF V600E, ATC with BRAF V600E, solid tumors with BRAF V600E
MEK	Cobimetinib (Cotellic)	Genentech/Exelixis	2015	MEK1, MEK2	Serine‐threonine	Oral	In combination with vemurafenib: melanoma with a BRAF V600E or V600K
MEK	Binimetinib (Mektovi)	Array BioPharma	2018	MEK1, MEK2	Serine‐threonine	Oral	In combination with encorafenib: melanoma with a BRAF V600E or V600K
MEK	Selumetinib (Koselugo)	Astra Zeneca	2020	MEK1, MEK2	Serine‐threonine	Oral	Neurofibromatosis type 1
PI3K	Idelalisib (Zydelig)	Gilead Sciences	2014	PI3Kδ	Phosphatidylinosi‐tol 3‐kinase	Oral	CLL
PI3K	Copanlisib (Aliqopa)	Bayer Healthcare	2017	PI3Kα, PI3Kδ	Phosphatidylinosi‐tol 3‐kinase	Intravenous	FL
PI3K	Duvelisib (Copiktra)	Secura	2018	PI3Kδ, PI3Kγ	Phosphatidylinosi‐tol 3‐kinase	Oral	CLL/SLL
PI3K	Alpelisib (Piqray)	Novartis	2019	PI3Kα	Phosphatidylinosi‐tol 3‐kinase	Oral	HR‐positive, HER2‐negative breast cancer, PIK3CA‐mutated breast cancer (in combination with fulvestrant)
PI3K	Umbralisib (Ukoniq)	TG Theraps	2021	PI3Kδ, CK1ε	Phosphatidylinosi‐tol 3‐kinase	Oral	MZL, FL
JAK	Ruxolitinib (Jakafi)	Incyte	2011	JAK1, JAK2	Tyrosine	Oral	Myeloproliferative neoplasms
JAK	Fedratinib (Impact)	Impact	2019	JAK2	Tyrosine	Oral	Myeloproliferative neoplasms
CYC	Palbociclib (Ibrance)	Pfizer	2015	CDK4, CDK6	Serine‐threonine	Oral	HR‐positive, HER2‐negative breast cancer
CYC	Ribociclib (Kisqali)	Novartis	2017	CDK4, CDK6	Serine‐threonine	Oral	HR‐positive, HER2‐negative breast cancer
CYC	Abemaciclib (Verzenio)	Eli Lilly	2017	CDK4, CDK6	Serine‐threonine	Oral	HR‐positive, HER2‐negative breast Cancer
CYC	Trilaciclib (Cosela)	G1 Therap	2021	CDK4, CDK6	Serine‐threonine	Intravenous	Prevent chemotherapy‐induced myelosuppression in SCLC
BTK	Ibrutinib (Imbruvica)	Sandoz	2013	BTK	Tyrosine	Oral	MCL, CLL/SLL, CLL/SLL with 17p deletion, WM, MZL
BTK	Acalabrutinib (Calquence)	AstraZeneca	2017	BTK	Tyrosine	Oral	MCL, CLL/SLL
BTK	Zanubrutinib (Brukinsa)	BeiGene	2019	BTK	Tyrosine	Oral	MCL, WM, MZL
IDH1 and IDH2	Enasidenib (Idhifa)	CelGene	2017	IDH1 and IDH2	Tyrosine	Oral	IDH2 mutant AML
IDH1 and IDH2	Ivosidenib (Tibsovo)	Servier	2018	IDH1 and IDH2	Tyrosine	Oral	IDH1 mutant AML, IDH1 mutant cholangiocarcinoma
SRC	Tirbanibulin (Klisyri)	Almirall	2020	SRC	Tyrosine	Opical	Actinic keratosis

Abbreviations: Ph‐positive CML, Philadelphia chromosome‐positive chronic myeloid leukemia; Ph‐positive ALL, Philadelphia chromosome positive acute lymphoblastic leukemia; MDS/MPD, myelodysplastic/myeloproliferative diseases; ASM, aggressive systemic mastocytosis; HES, hypereosinophilic syndrome; CEL, chronic eosinophilic leukemia; DFSP, dermatofibrosarcoma protuberans; GIST, gastrointestinal stromal tumors; NSCLC, nonsmall cell lung cancer; MTC, medullary thyroid cancer; AML, acute myeloid leukemia; SM‐AHN, systemic mastocytosis with associated hematological neoplasm; MCL, mantle cell lymphoma; CRC, colorectal cancer; ATC, anaplastic thyroid cancer; CLL/SLL, chronic lymphocytic leukemia/small lymphocytic lymphoma; FL, follicular lymphoma; MZL, marginal zone lymphoma; MCL, mantle cell lymphoma; WM, waldenström's macroglobulinemia. Data sources: https://www.fda.gov/drugs/development‐approval‐process‐drugs/drug‐approvals‐and‐databases.

### 3.1 ABL1 kinase inhibitors

Philadelphia (Ph) chromosome translocation results in a fusion between the abelson murine leukemia viral oncogene homolog 1 (ABL1) on chromosome 9 and the breakpoint cluster region (BCR) gene on chromosome 22, forming an aberrant BCR–ABL1 fusion gene on chromosome 22, which encodes a 210 kDa tyrosine kinase. This BCR–ABL1 protein further triggers phosphorylation of numerous substrates to activate MAPK, PI3K/AKT/mTOR, and JAK/STAT pathways, thereby driving the uncontrolled proliferation of leukemia cells. Nearly all of patients with chronic myeloid leukemia (CML) and 20–30% of patients with acute lymphoblastic leukemia (ALL) have BCR–ABL1 gene fusions, which are the driving molecular abnormality of these diseases. Correspondingly, BCR–ABL1 fusion tyrosine kinase is a crucial target in treatment of certain leukemias.

Imatinib, initially approved for treating BCR–ABL1‐driven CML by the US FDA in 2001, opens a new era in using protein kinase inhibitors in cancer treatment.^36^ Imatinib binds to a catalytically inactive conformation of the BCR–ABL1 kinase domain (type II). In the phase III IRIS study, patients with newly diagnosed CML in chronic phase (CML‐CP) were assigned to receive imatinib or interferon alfa plus low‐dose cytarabine. At 18 months, the rate of major cytogenetic response was 87.1% (95%CI: 84.1, 90.0) in the imatinib arm, as compared with 34.7% (95%CI: 29.3, 40.0) in the group that received interferon alfa plus cytarabine (*p* < 0.001). The rates of complete cytogenetic response (CCyR) were 76.2% (95%CI: 72.5, 79.9) and 14.5% (95%CI, 10.5, 18.5), respectively (*p* < 0.001).^37^ Long‐term follow‐up of IRIS trial participants revealed a 5‐year survival rate of 89% and a 10‐year survival rate of 83.3% with infrequent serious side effects. The 5‐year and 10‐year CCyR rates in imatinib group were 87 and 82.8%, respectively.^38^, ^39^ Though imatinib significantly improves outcomes in patients with BCR–ABL1‐driven CML, resistance emerges unavoidably after long‐term treatment, such as M244, G250, Q252, Y253, and E255 located in the P loop, T315 and F317 in the ATP‐binding region, M351 and F359 in SH2 contact and C‐lobe region, and H396 in the activation loop.^40^ Second‐ and third‐generation inhibitors dasatinib, nilotinib, bosutinib, and ponatinib can mitigate resistance caused by specific point mutations in BCR–ABL1 kinase domain.^41^, ^42^, ^43^, ^44^ Dasatinib and bosutinib bind at the ATP site in an active conformation of the ABL1 kinase domain (type I) and are demonstrated to bind to ABL1 kinase domain much stronger than that of imatinib. The binding mode of nilotinib is similar to imatinib, but with a 50‐fold higher BCR–ABL1 inhibitory activity in vitro than imatinib. Dasatinib, nilotinib, and bosutinib are not only efficacious after imatinib failure but have also been shown to be superior to imatinib when used in the first‐line treatment of CML‐CP, with deeper molecular responses.^45^, ^46^, ^47^ The T315I mutant produces resistance to imatinib, dasatinib, nilotinib, and bosutinib. Ponatinib shows its advantage to overcome resistance from T315I mutation and is approved as a rescue strategy.^48^, ^49^, ^50^, ^51^, ^52^ The above‐mentioned BCR–ABL1 inhibitors all belong to ATP competitive inhibitors, which are difficult to limit their targets only to BCR–ABL1.^48^, ^49^, ^50^, ^51^, ^52^ In 2021, the US FDA approved an allosteric inhibitor, asciminib, to treat Ph‐positive CML‐CP with the T315I mutation and for third‐line therapy of Ph‐positive CML‐CP. As it binds to the ABL1 myristoyl pocket, its targets are limited to BCR–ABL1 and mutated BCR–ABL1, including the gatekeeper T315I mutant. The side effects associated with imprecise targets are significantly reduced.^53^, ^54^, ^55^

Three ABL1 kinase inhibitors (imatinib, dasatinib, and ponatinib) are granted US FDA approval for Ph‐positive ALL. Though the efficacy of single‐agent tyrosine kinase inhibitor (TKI) therapy for Ph‐positive ALL is poor, the combination of ABL1 inhibitors with conventional chemotherapy largely improves the response and survival and now is standard of care for Ph‐positive ALL.^56^ Adding imatinib to hyper‐CVAD led 93% (42 out of 45) of patients to achieve complete remission (CR) and 48% of patients to live beyond 3 years.^57^ The combination of dasatinib with hyper‐CVAD further improved the prognosis of Ph‐positive ALL, with the CR rate and 3‐year survival rate reaching 96 and 60%, respectively.^58^ The occurrence of T315I kinase domain mutations in up to 75% of patients and low complete molecular remission (CMR) rates (∼50% with high‐intensity combination) after treatment with first‐ and second‐generation ABL1 inhibitors requires the outcome of third‐generation ABL1 inhibitor ponatinib, which is more potent and might further improve outcomes by inducing higher CMR rates and suppress the emergence of T315I mutations.^52^, ^59^ The CMR rate was 84%, the CR rate reached 98%, and the 3‐year survival rate was 79% for patients in the ponatinib plus hyper‐CVAD group.^60^ However, careful dose adjustment was recommended for ponatinib when combined with hyper‐CVAD in order to avoid serious toxic effects, such as vascular events and pancreatitis.^61^, ^62^ Furthermore, the high remission rate of ABL1 inhibitor plus chemotherapy decreases the proportion of patients underwent allogeneic hematopoietic stem cell transplantation (allo‐HSCT) and increases the probability of patients to proceed to allo‐HSCT.^63^ There are other combination regimes for treating Ph‐positive ALL, including low‐intensity chemotherapy and chemotherapy‐free combination. ABL1 kinase inhibitor is combined with low‐intensity chemotherapy or steroids in patients 60 years and older and those unable to receive intensive chemotherapy.^64^ The chemotherapy‐free regimens of ABL1 inhibitor dasatinib or ponatinib and a CD3–CD19 bispecific antibody blinatumomab represent a new trend because of their safety and excellent results in frontline setting. Notably, phase II studies showed that patients received a CMR rate of 86% and a 3‐year survival rate of 94% in the combination of ponatinib plus blinatumomab group.^52^

Researches on ABL1 kinase inhibitors are ongoing, mainly focused on three directions. First, new ABL1 kinase inhibitors still arouse great interest. Both flumatinib and radotinib belong to second‐generation ABL1 kinase inhibitors and have been approved for Ph‐positive CML‐CP in China and Korea, respectively. In addition, both of them have been proved to be superior to imatinib in first‐line therapy for newly diagnosed CML‐CP.^65^, ^66^ Resistance to ponatinb requires the development of new T315I‐targeted inhibitors. The third‐generation ABL1 kinase inhibitor olverembatinib was licensed in China for TKI‐resistant CML‐CP or accelerated‐phase CML harboring the T315I mutation.^67^ Vodobatinib is a third‐generation ABL1 kinase inhibitor and is in phase I/II stage for treatment‐refractory/intolerant CML (NCT02629692). Rebastinib is a noncompetitive conformational control inhibitor designed to overcome BCR–ABL1 gatekeeper mutations. However, phase I study (NCT00827138) failed to show sufficient clinical benefit.^68^ PF‐114 is a fourth‐generation ABL1 kinase inhibitor. Phase I/II study (NCT02885766) of this agent is recruiting patients with Ph‐positive CML whose disease is resistant to the second‐generation ABL1 inhibitors or has T315I mutation in the BCR–ABL gene. Second, combination with other mechanism drugs to increase efficacy or overcome drug resistance is also popular. A phase III study (NCT04530565) is recruiting patients with Ph‐positive ALL to compare the effect of usual treatment of chemotherapy and steroids and an ABL1 inhibitor to the same treatment plus blinatumomab. Combined with BCL‐2 inhibitor venetoclax is another choice. The efficacy and safety of dasatinib plus venetoclax for Ph‐positive CML is assessed in a phase II study (NCT02689440). A potent combination regime concluding decitabine, venetoclax, and ponatinib for Ph‐positive ALL or Ph‐positive myeloid blast phase or accelerate phase CML is under estimation in a phase II study (NCT04188405). Combination with immune checkpoint inhibitors (ICIs) is also worth expectance. A phase II study (NCT03516279) is assessing pembrolizumab and ABL1 kinase inhibitor (dasatinib, imatinib, or nilotinib) for patients with CML and persistently detectable minimal residual disease. Third, dose reduction or discontinuation has also been studied. The standard dose of dasatinib for CML‐CP in adults is 100 mg once daily, which is associated with myelosuppression and pleural effusions. Dasatinib at a lower dose of 50 mg daily was demonstrated to be active and well tolerated in patients with newly diagnosed CML‐CP in phase II studies.^69^, ^70^ Cessation of ABL1 kinase inhibitors has been thoroughly and continuously studied. The conclusion of clinical trials supported discontinuation in patients with a confirmed deep molecular response for at least 1 year.^71^, ^72^

### 3.2 KIT receptor kinase inhibitors

Stem cell factor, also known as c‐KIT ligand, exerts its biological functions by binding to and activating the receptor tyrosine kinase (RTK) c‐KIT, also known as stem cell growth factor receptor. Dimerization of c‐KIT leads phosphorylation and activation of downstream kinases in PI3K/AKT/mTOR, MAPK, and JAK/STAT pathways and further maintains cell survival, migration, and proliferation.^73^ c‐KIT is mainly expressed in stem cells, progenitor cells, and other cells with self‐renewal potency. However, dysregulation of c‐KIT can promote tumor formation and progression. Overexpression or gain of function mutations of c‐KIT has been reported in various cancers, such as gastrointestinal stromal tumors (GISTs), small‐cell lung carcinomas (SCLC), advanced systemic mastocytosis, and acute myeloid leukemia (AML).^74^

About 99% of GISTs have an identifiable driver alteration. The KIT (mostly in exon 11, followed by exon 9) and PDGFRA (mainly in exon 18 and less frequently in exon 12 or 14) mutations are the two major molecular subtypes that disrupt autoinhibitory regions of the RTK and thereby result in ligand‐independent activation, occurring in around 70 and 15% of all GISTs, respectively. GISTs without KIT and PDGFRA mutations can be divided into SDH‐deficient and SDH‐competent GISTs.^75^, ^76^

Before 2000, no effective therapies were available for patients with advanced GISTs, while imatinib revolutionized the treatment of patients with this disease.^77^ Imatinib inhibits not only BCR–ABL1 but also the kinases of the KIT and PDGFA receptors.^78^, ^79^ Mutations in KIT exon 11, 9, and 13 confer sensitivity to imatinib, while most KIT exon 17 mutations are considered to be resistant to imatinib.^76^ In advanced GISTs with KIT mutations, the response to imatinib of advanced GISTs with KIT exon 11 mutations is higher than that of GISTs with exon 9 mutations, especially when using standard‐dose imatinib (400 mg total daily dose). Heinrich et al. reported that the partial response (PR) rate in patients with GISTs harboring exon 11 KIT mutations was 83.5%, whereas the percent decreased to 47.8% in patients with tumors containing an exon 9 KIT mutation. With a median follow‐up of 19 months, patients whose tumors contained exon 11 KIT mutations had a longer event‐free survival (EFS) (687 vs. 200 days; *p* < 0.0001) and overall survival (OS) (*p* = 0.0034) than those whose tumors expressed exon 9 KIT mutations.^80^ The data from the North American phase III SWOG S0033/CALGB 150105 study further confirmed the favorable impact of KIT exon 11 mutations when compared with KIT exon 9 mutations for the objective response rate (ORR) (71.7 vs. 44.4%; *p* = 0.007), median time to tumor progression (24.7 vs. 16.7 months; *p* = 0.0013), and median OS (60.0 vs. 38.4 months; *p* = 0.011).^81^ Imatinib is also used in the adjuvant setting for patients with GISTs harboring KIT or PDGFRA mutations that confer imatinib sensitivity. Compared with placebo, 12‐month imatinib prolonged recurrence‐free survival (RFS) compared with placebo (98 vs. 83%; HR, 0.35; 95%CI: 0.22, 0.53; *p* < 0.0001).^82^ Patients with KIT exon 11 deletions assigned to 1 year of adjuvant imatinib had a longer RFS.^83^ Three‐year imatinib further prolonged 5‐year RFS (65.6 vs. 47.9%; HR, 0.46; 95%CI: 0.32, 0.65; *p* < 0.001), significantly prolonged 5‐year OS (92.0 vs. 81.7%; HR, 0.45; 95%CI: 0.22, 0.89; *p* = 0.02), compared with 1‐year imatinib.^84^ In the 3‐year imatinib group, 5‐year OS and 10‐year OS rates were 92.0 and 79.0%, respectively, and in the 1‐year imatinib group 85.5 and 65.3%, respectively.^85^ Research also showed that the benefit only existed in patients with KIT exon 11 deletion or insertion‐deletion mutation whose 5‐year RFS increased from 41.3% in the 1‐year group to 71.0% in the 3‐year group (*p* < 0.001).^86^

The main imatinib‐resistance mechanisms are activating other signaling pathways and secondary KIT mutations. Second‐line sunitinib and third‐line regorafenib are both multikinase inhibitors and will be discussed below.^87^ The new type II kinase inhibitor, ripretinib, can bind to a novel region of both the KIT and PDGFRA kinases to force the activation loop into an inactive conformation and target a broad spectrum of KIT and PDGFRA mutations.^88^ In 2020, ripretinib was approved by the US FDA for fourth‐line therapy for patients with advanced GISTs who have received prior treatment with three or more kinase inhibitors, including imatinib, based on the data that ripretinib resulted in an ORR of 9% versus 0% with placebo (*p* = 0.05), a median progression‐free survival (PFS) of 6.3 months versus 1.0 months (HR, 0.15; 95%CI: 0.09,0.5; *p* < 0.0001) and median OS of 15.1 months versus 6.6 months (HR, 0.36; 95%CI: 0.21–0.62) in the phase III INVICTUS trial.^89^, ^90^ Subsequent analysis of the phase III INVICTUS trial uncovered that ripretinib inhibits a broad range of KIT/PDGFRα mutations based on the data that ripretinib provided PFS benefit regardless of mutation status in patients with advanced GISTs.^91^

Currently, there are still many researches on KIT kinase inhibitors. Like imatinib, other ABL1 kinase inhibitors also inhibit c‐KIT, such as dasatinib, nilotinib, and ponatinib. In a single‐arm phase II trial, ponatinib demonstrated activity in advanced GISTs with failure of prior TKI, particularly in subtype with KIT exon 11 mutations.^92^ However, nilotinib failed to show its advantages over imatinib in treating first‐line GISTs in a phase III trial.^93^ In addition to GISTs, KIT inhibitors have also been tried in other tumor types with KIT alterations, particularly in KIT‐altered melanoma. Imatinib pioneered the clinical trials in melanoma, followed by nilotinib, dasatinib, and regorafenib. Phase II trials showed that imatinib was effective in melanoma with KIT amplification and/or mutations, with ORR fluctuating from 16 to 29%.^94^, ^95^, ^96^ The value of ORR was similar in nilotinib‐treated/KIT‐altered melanoma but was much lower in dasatinib‐treated individuals.^97^, ^98^ A phase II study is recruiting patients with c‐KIT‐mutated melanoma for second‐line therapy with regorafenib (NCT02501551). The combination of KIT inhibitors with PD‐1 inhibitors is a new trend for melanoma with c‐KIT gene mutations (NCT05274438). In addition, a phase I trial (NCT02571036) is designed to evaluate ripretinib in patients with advanced malignancies. Most importantly, new KIT kinase inhibitors are still being developed. Masitinib is a potent and highly selective TKI with activity against the wild‐type c‐Kit receptor and its juxtamembrane mutation. A phase II study (NCT00998751) evaluated masitinib as the first‐line treatment of advanced GIST, which showed masitinib was comparable with imatinib in terms of safety and response.^99^ Masitinib also have been trialed in advanced GISTs after failure of imatinib. The results of a phase III trial (NCT01694277) ensured that the masitinib arm could satisfy a prespecified PFS threshold and received significantly longer OS with lower occurrence of severe adverse events compared with sunitinib.^100^ Masitinib has also been explored in many other tumor types, such as colorectal cancer (CRC), systemic mastocytosis, prostate cancer, ovarian cancer, and pancreatic cancer (NCT03556956, NCT04333108, NCT03761225, NCT02490488, and NCT00789633). Amuvatinib is a novel TKI with in vitro pharmacological activity against mutant KIT, PDGFRA, and Rad51. Phase I trial (NCT00894894) proved its safety profile and transient response in refractory GISTs and more trials are needed to prove its efficacy.^101^ AZD3229 is a pan‐KIT mutant kinase inhibitor that also targets PDGFRα. It is 15–60 times more potent than imatinib in inhibiting KIT primary mutations and has low nanomolar activity against a broad spectrum of secondary mutations. However, it is still in the preclinical stage.^102^ In total, the expansion of the indications of existing drugs and the development of novel KIT inhibitors are the main research directions.

### 3.3 PDGFR kinase inhibitors

PDGFRs (PDGFRA and PDGFRB), activated in PDGF‐dependent or PDGF‐independent modes, phosphorylate substrates and engage in signaling cascades that drive physiological or pathological functions.^103^ In cancers, the PDGF/PDGFR system influences tumor growth, metastasis, and drug response through direct impact on tumor cells or indirect impact on tumor stromal fibroblasts and perivascular cells. Point mutations, rearrangements, and amplification of genetic alterations in tumor cells are known to activate PDGFRs.^104^ PDGFR‐signaling in malignant cells with PDGFRA alterations are the main targets, such as PDGFRA‐mutated GISTs. At the same time, the expression of PDGFR in the extracellular matrix is associated with angiogenesis. The activation of PDGF/PDGFR pathway is one potential resistance mechanism to VEGFR2 inhibition. High expression of PDGFR in stromal fibroblasts and perivascular cells can be found in various cancers, such as breast, gastric, colorectal, kidney, ovarian, and pancreatic cancer, and predicts poor prognosis in these tumor types.^105^

As mentioned above, 15% of all GISTs occur PDGFRA mutations. Imatinib‐sensitive PDGFRA mutants include mutations in exon 12, 14, or indels in exon 18. GISTs with PDGFRA exon 18 D842V variant leads to primary resistance to imatinib and other type II PDGFRA/KIT TKIs and predicts poor outcomes for patients with GISTs. The D842V mutation, the most common PDGFRA mutation (9–10% of all primary GISTs), became a key target for rational drug design.^76^, ^106^ In 2020, the US FDA approved the type I PDGFRA/KIT TKI avapritinib for patients with advanced GISTs harboring a PDGFRA exon 18 mutation (including D842V).^107^, ^108^ In the phase I NAVIGATOR trial, the ORR reached 91 and 61% of patients responded over 6 months.^109^ After a median follow‐up of 27.5 months, the ORR, median duration of response (DOR), and median PFS were 91%, 27.6 months, and 34.0 months, respectively. Median OS was not reached with a manageable safety profile.^110^ The above data showed avapritinib resulted in an unprecedented and durable clinical benefit in patients with PDGFRA D842V‐mutant GISTs. However, avapritinib failed to prolong PFS in patients with molecularly unselected, late‐line GISTs compared with regorafenib.^111^ The effect of avapritinib in treating malignant solid tumors with c‐KIT or PDGFRA mutations is under research (NCT04771520).

A large number of multikinase TKIs with inhibitory activity toward PDGFR are used to target stromal PDGFR expression, including sorafenib for hepatocellular carcinoma (HCC), renal cell carcinoma (RCC), and radioiodine‐refractory DTC and pazopanib for RCC and soft tissue sarcoma (STS). However, tumors often cannot be controlled through single inhibition of PDGFR expression in the stroma, which is proved by two PDGFRA antibody, olaratumab and MEDI‐575. Olaratumab, combined with doxorubicin, is approved for the treatment of adult patients with STS based on a phase II trial, in which the researchers found olaratumab plus doxorubicin improved PFS and OS compared with monotherapy with doxorubicin.^112^ But the conclusion was overturned by the phase III ANNOUNCE trial. In this phase III clinical trial, the addition of olaratumab to doxorubicin resulted in no significant difference in OS.^113^ Though the safety profile of PDGFRA antibodies is acceptable, the prognosis cannot be improved by olaratumab and MEDI‐575 in various tumor types, including ovarian cancer, glioma, lung cancer, and prostate cancer.^114^, ^115^, ^116^, ^117^ On the contrary, positive results can be obtained by using olaratumab to inhibit PDGFR alterations in tumor cells. Olaratumab prolonged disease control in previously treated patients with PDGFRA D842V‐mutant GISTs compared with historical data.^118^

Some PDGFR kinase inhibitors are undergoing clinical trials. Crenolanib is a highly specific PDGFR kinase inhibitor and is proved to be safe in several phase I clinical trials.^119^, ^120^ A phase II study (NCT01243346) evaluated the antitumor efficacy and pharmacokinetics of crenolanib in patients with D842‐mutant GISTs. A phase III trial (NCT02847429) is ongoing to assess crenolanib in D842‐mutant GISTs. Similar to PDGFR antibodies, single inhibition of PDGFR in tumor stroma by crenolanib is not an ideal method to control tumors. Another phase II study (NCT01229644) was designed to evaluate the antitumor efficacy of crenolanib in patients with recurrent high‐grade glioma and in patients with low‐grade glioma, which was terminated ahead because of poor efficacy. The same situation happens to a type III PDGFRβ kinase inhibitor, tandutinib, which can only be used to inhibit the expression of PDGFR in tumor stroma. The results of phase II studies (NCT00379080 and NCT00408902) of tandutinib on glioblastomas or RCC did not meet the primary end points.^121^, ^122^ In the future, selective PDGFR kinase inhibitors should preferably only target tumor cells with PDGFR alterations.

### 3.4 HER kinase inhibitors

The EGFR or HER family members comprise four structurally related RTKs that are EGFR/HER1, HER2, HER3, and HER4.^123^ PI3K/AKT/mTOR, MAPK, PLCγ, and JAK/STAT are the four major representative downstream signaling pathways activated by EGFR or HER family members, which are related with tumorigenesis, tumor growth, and progression. Mutations in EGFR/HER1 tyrosine kinases play an essential role in tumor growth and progression, especially for nonsmall cell lung cancer (NSCLC).^124^, ^125^ HER2 overexpression or amplification has been observed in approximately 15–30% of breast cancer and the HER2‐positive subtype predicts a worse prognosis than the HER2‐negative breast cancer.^126^, ^127^

From 2003, the treatment for NSCLC progressed significantly. First‐generation EGFR TKIs, gefitinib and erlotinib, were originally designed for patients with overexpression of wild‐type EGFR.^128^ In the following clinical trials, researchers found that these inhibitors are more sensitive to tumors with EGFR exon 19 deletion or exon 21 (L858R) mutations.^129^, ^130^, ^131^ The US FDA has approved both gefitinib and erlotinib as first‐line treatment for NSCLC with EGFR exon 19 deletion or exon 21 (L858R) mutations, based on the evidence that they can significantly improve median PFS and ORR, compared with chemotherapy. First‐line gefitinib prolonged median PFS from 5.4 months in the paclitaxel plus carboplatin group to 10.8 months (HR, 0.30; 95%CI: 0.22, 0.41; *p* < 0.001) and ORR from 30.7% to 73.7% (*p* < 0.001).^132^ In Chinese patients with advanced EGFR mutation‐positive NSCLC, median PFS was 13.1 months with erlotinib and 4.6 months with chemotherapy (HR, 0.16; 95%CI: 0.10, 0.26; *p* < 0.0001), which was 9.7 months and 5.2 months (HR, 0.37; 95%CI: 0.25, 0.54; *p* < 0.0001), respectively, in European patients.^133^, ^134^ As pancreatic cancer overexpresses EGFR, erlotinib combined with gemcitabine was also approved for first‐line treatment for pancreatic cancer because OS was significantly longer with erlotinib plus gemcitabine than with gemcitabine (median OS: 6.24 vs. 5.91 months; HR, 0.82; 95%CI: 0.69, 0.99; *p* = 0.038).^135^

Second‐generation EGFR TKIs, afatinib and dacomitinib, bind irreversibly to the ATP pocket of EGFR TK, developed to circumvent drug resistance or increase efficacy.^136^, ^137^ In 2018, the US FDA approved afatinib to treat NSCLC with nonresistant EGFR mutations. The LUX‐Lung 3 trial showed the median PFS increased from 6.9 months in the pemetrexed plus cisplatin arm to 11.1 months in the afatinib arm (HR, 0.58; 95%CI: 0.43, 0.78; *p* = 0.001), ORR from 19.1 to 50.4%.^138^ The LUX‐Lung 6 trial further consolidated afatinib in first‐line treatment of NSCLC harboring EGFR mutations, which showed first‐line afatinib significantly improved PFS compared with gemcitabine plus cisplatin (median PFS: 11.0 vs. 5.6 months; HR, 0.28; 95%CI: 0.2, 0.39; *p* < 0.0001).^139^ Post‐hoc analysis of LUX‐Lung 2, LUX‐Lung 3, and LUX‐Lung 6 indicated that afatinib was active in NSCLC harboring certain types of uncommon EGFR mutations, such as Gly719Xaa, Leu861Gln, and Ser768Ile, but was inactive in NSCLC with T790M and exon 20 insertion mutations.^140^ The LUX‐Lung 7 trial demonstrated that afatinib slightly prolonged PFS, time‐to‐treatment failure, and ORR and exhibited a manageable tolerability profile when compared with gefitinib for first‐line treatment of EGFR mutation‐positive NSCLC.^141^ What is more, afatinib has also been approved to treat patients with squamous NSCLC who progressed on platinum‐based doublet chemotherapy in 2016, based on the LUX‐Lung 8 trial, in which median OS (8.4 vs. 6.6 months; HR, 0.81; 95%CI: 0.62, 1.05; *p* = 0.12) and median PFS (3.5 vs. 2.5 months; HR, 0.69; 95%CI: 0.51, 0.92; *p* = 0.01) of afatinib were slightly better than those of erlotinib.^142^ While another second‐generation EGFR TKI dacomitinib, a pan‐HER inhibitor, demonstrated its advantages over gefitinib in median PFS (14.7 vs. 9.2 months; HR, 0.59; 95%CI: 0.47, 0.74; *p* < 0.0001) and median OS (34.1 vs. 26.8 months; HR, 0.76; 95%CI: 0.58, 0.99; *p* = 0.04), was also approved for first‐line treatment for NSCLC with EGFR exon 19 deletion or exon 21 (L858R) mutations.^143^, ^144^

Osimertinib, third‐generation EGFR‐TKI, binds irreversibly to the activating and T790M mutation at approximately ninefold lower concentrations than wild‐type EGFR. Surprisingly, osimertinib can distribute to the brain at plasma AUC ratios of approximately 2 following oral dosing.^145^ The phase III FLAURA trial demonstrated that osimeritinib significantly improved median PFS from 10.2 months in erlotinib or gefitinib to 18.9 months in osimeritinib (HR, 0.46; 95%CI: 0.37, 0.57; *p* < 0.0001) in first‐line therapy. In FLAURA, osimeritinib also showed its advantage in controlling central nervous system (CNS) lesions over erlotinib or gefitinib.^146^ After a long‐term follow‐up for OS, the duration of median OS in the osimeritinib group was 38.6 months and 31.8 months in the comparator group (erlotinib or gefitinib) (HR, 0.80; 95.05%CI: 0.64 to 1.00; *p* = 0.046).^147^ When used for previously treated EGFR T790M mutation‐positive NSCLC, osimeritinib increased median PFS from 4.4 months in chemotherapy to 10.1 months in osimeritinib (HR, 0.30; 95%CI: 0.23, 0.41; *p* < 0.001), ORR from 29 to 65%. Because of its merits, it has been approved for first‐line treatment for NSCLC with EGFR exon 19 deletion or exon 21 (L858R) mutations and EGFR T790M mutation‐positive NSCLC in 2018 and 2015, respectively. Osimertinib has also made great strides in adjuvant therapy and has already been allowed for adjuvant therapy for NSCLC with EGFR exon 19 deletion or exon 21 (L858R) mutations in 2020. The phase III ADAURA trial showed that median disease‐free survival (DFS) was not reached and 19.6 months in placebo (HR, 0.17; 95%CI: 0.12, 0.23; *p* < 0.0001). However, the OS data were immature.^146^, ^147^, ^148^

EGFR exon 20 insertion is another observable mutation type and associated with a poor prognosis, accounting for about 1–12% of EGFR mutations.^149^ The above EGFR TKIs cannot be applied for NSCLC with EGFR exon 20 insertion. A new EGFR TKI, mobocertinib, changed this situation in 2021. The ORR was 28% and the investigator‐assessed confirmed response rate was 43% with median DOR of 14 months in patients with EGFR exon 20 insertion mutations whose disease has progressed on or after platinum‐based chemotherapy.^150^ However, more clinical trials are needed to verify its efficacy.

HER2‐targeted therapy is important for patients with HER2‐positive advanced breast cancer. First‐line treatment with the combination of trastuzumab and pertuzumab plus taxane and second‐line treatment with trastuzumab deruxtecan significantly improve the outcome of patients with advanced or metastatic HER2‐positive breast cancer, leading to great enthusiasm to develop novel anti‐HER2 agents. HER kinase inhibitors for breast cancer are primarily approved in patients whose disease refractory to traditional HER2‐directed therapies. The addition of lapatinib to capecitabine after trastuzumab plus chemotherapy in patients with HER2‐positive breast cancer has advantages in the time to progression (TTP), from 4.4 months in the placebo plus capecitabine group to 8.4 months in the lapatinib plus capecitabine group (HR,0.49; 95%CI: 0.34, 0.71; *p* < 0.001).^151^ Furthermore, the phase III NALA trial demonstrated that neratinib combined with capecitabine improved PFS (HR, 0.76; 95%CI: 0.63, 0.93; *p* = 0.0059) and time to intervention for CNS disease compared with lapatinib plus capecitabine.^152^ In the phase II HER2CLIMB trial, another HER kinase inhibitor, tucatinib, was added to trastuzumab plus capecitabine after one or more prior anti‐HER2‐based regimens not only extended PFS (HR, 0.54; 95%CI: 0.42, 0.71; *p* < 0.001) and OS (HR, 0.66; 95%CI: 0.50, 0.88; *p* = 0.005) in patients with HER2‐positive metastatic breast cancer but also resulted in better PFS (median PFS: 7.6 vs. 5.4 months; HR, 0.48; 95%CI: 0.34, 0.69; *p* < 0.001) in those with brain metastases.^153^ Further analyses in patients with brain metastases showed that the addition of tucatinib to trastuzumab and capecitabine reduced the risk of intracranial progression or death by 68%, doubled duration of median CNC‐PFS (intracranial progression or death), and prolonged OS.^154^ Based on the above clinical trials, they are allowed to use in patients with advanced or metastatic HER2‐positive breast cancer who have received prior anti‐HER2‐based regimens. In addition, lapatinib combined with letrozole is granted approval by the US FDA for first‐line therapy of HR‐positive and HER2‐positive metastatic breast cancer.^155^ The US FDA also approved neratinib in using extended adjuvant treatment of HER2‐positive breast cancer following adjuvant trastuzumab‐based therapy, based on its ability to prolong invasive DFS.^156^

Research on already approved HER kinase inhibitors and novel HER kinase inhibitors will give us more choices. Already approved HER kinase inhibitors are mainly for EGFR‐mutant NSCLC and HER2‐positive breast cancer. To treat NSCLC, monotherapy with gefitinib, erlotinib, afatinib, or dacomitinib, is successfully used in advanced NSCLC with EGFR exon 19 deletion or exon 21 (L858R) mutations. The third‐generation EGFR TKI osimertinib is demonstrated to be superior to the first‐ and second‐generation EGFR TKIs and used in EGFR T790M mutation‐positive NSCLC and first‐line treatment and adjuvant therapy for NSCLC with EGFR exon 19 deletion or exon 21 (L858R) mutations.^124^ Combination therapy is widely studied for EGFR TKIs. First, the results of EGFR TKI combined with chemotherapy are encouraging. Compared with gefitinib alone, gefitinib combined with carboplatin plus pemetrexed improved PFS in patients with untreated advanced NSCLC with EGFR mutations.^157^ However, the continuation of gefitinib after progression on first‐line gefitinib did not prolong PFS in patients who received platinum‐based doublet chemotherapy as second‐line treatment.^158^ Dual inhibition of EGFR and VEGF pathways presents another trend. Bevacizumab plus erlotinib significantly improved PFS in patients with untreated metastatic EGFR‐mutated NSCLC but not OS.^159^ On the contrary, adding bevacizumab to osimertinib failed to show prolongation of PFS in patients with advanced lung adenocarcinoma with EGFR T790M mutation.^160^ Previous studies showed that ICIs was not active in patients with EGFR‐mutant or ALK‐rearrangement NSCLC.^161^ Several combinations of ICIs and EGFR‐TKIs are being evaluated in TKI‐failed EGFR mutant patients. Preclinical research suggested that EGFR inhibitors could increase the efficacy of immunotherapy in lung adenocarcinomas.^162^ However, the phase Ib TATTON study indicated that osimertinib plus durvalumab was not feasible due to increased reporting of interstitial lung disease.^163^ More trials are needed to show whether they can be combined. Studies offer us several single agents or combination regimes to treat previously untreated patients with advanced EGFR‐mutated NSCLC. However, which one is the most suitable? Zhao included 18 trials involved 4628 patients and 12 treatments and used network meta‐analysis to compare the efficacy and safety of first‐line treatments for these individuals. Twelve treatments were EGFR TKIs (osimertinib, dacomitinib, afatinib, erlotinib, gefitinib, and icotinib), pemetrexed‐based chemotherapy, pemetrexed free chemotherapy, and combination treatments (afatinib plus cetuximab, erlotinib plus bevacizumab, gefitinib plus pemetrexed‐based chemotherapy, and gefitinib plus pemetrexed). The authors found that osimertinib resulted in the best PFS for patients with EGFR exon 19 deletion and gefitinib plus pemetrexed‐based chemotherapy gave superiority for patients with EGFR exon 21 (L858R) mutations.^164^ In addition, many new third‐generation EGFR TKIs for EGFR T790M‐mutated NSCLC are emerging. Icotinib, furmonertinib, and almonertinib have been approved in China and olmutinib got approval in Korea.^165^, ^166^ Abivertinib also belongs to third‐generation EGFR TKI and is in the phase III stage for NSCLC (NCT03856697). To date, mobocertinib is the only approved EGFR TKI for second‐line therapy of NSCLC with EGFR exon 20 insertions.^150^ A phase III study (NCT04129502) is underway to evaluate mobocertinib as a first‐line treatment versus platinum‐based chemotherapy for NSCLC with EGFR exon 20 insertions. A new EGFR‐TKI poziotinib is an irreversible pan‐HER TKI that targets EGFR, HER2, and HER4 and showed an ORR of 27.8% in second‐line therapy for NSCLC with EGFR exon 20 insertions in a phase II study (NCT03318939).^167^ A phase III study (NCT05378763) is ongoing to further verify the efficacy of poziotinib for NSCLC with EGFR exon 20 insertions. As a pan‐HER TKI, poziotinib has also been tested in other tumor types and yielded promising antitumor efficacy with manageable toxicity in HER2‐positive tumors, such as gastric cancer, breast cancer, and head and neck squamous cell carcinoma.^168^, ^169^ HER kinase inhibitors are permitted for post‐line treatment and subsequent adjuvant therapy of HER2‐positive breast cancer and first‐line therapy of HER2‐positive, HR‐positive breast cancer. Particularly, the neoadjuvant phase III ALTERNATIVE trial showed the addition of lapatinib to trastuzumab plus an aromatase inhibitor in postmenopausal women with HER2‐positive, HR‐positive metastatic breast cancer doubled the duration of PFS without adding adverse events.^170^ Other HER kinase inhibitors are also explored. Pyrotinib is an emerging irreversible EGFR/HER2 dual TKI and has been approved in HER2‐positive breast cancer in China.^171^

### 3.5 ALK and ROS1 kinase inhibitors

ALK is a RTK within the insulin receptor family, comprised of an extracellular region, a single transmembrane domain, and an intracellular kinase region. ALK fusion proteins interact with a complex network of proteins and thereby drive aberrant proliferation and survival through PI3K/AKT/mTOR, MAPK, and JAK/STAT pathways. ROS proto‐oncogene 1 (ROS1) is a RTK with an unknown physiological role in humans, while ROS1 fusion proteins drive occurrence of various tumors, including glioblastomas, NSCLC, and IMTs. Though ALK and ROS1 fusion proteins existed in multiple tumor types, ALK and ROS1 kinases inhibitors are only approved for ALK or ROS1‐positive NSCLC before July 14, 2022. ALK and ROS1 rearrangements occur in 3–8% and 0.9‐2.6% of the overall NSCLC population, mostly on adenocarcinomas, with higher prevalence in nonsmokers, younger age and early‐stage brain metastasis.^124^, ^172^

The first ALK/ROS1 inhibitor, crizotinib, was initially approved in 2011. The PROFILE 1014 and the PROFILE 1007 trials confirmed a survival superiority of crizotinib over platinum‐based chemotherapy in first‐line and second‐line therapy (after 1 platinum‐based chemotherapy regimen) for ALK‐positive NSCLC, respectively.^173^, ^174^ Compared with chemotherapy, crizotinib extended the median PFS from 7.0 months to 10.9 months (HR, 0.45; 95%CI: 0.35, 0.60; *p* < 0.001) and upgraded ORR from 45% to 74% in first‐line treatment, median PFS from 3.0 months to 7.7 months (HR, 0.49; 95%CI: 0.37, 0.64; *p* < 0.001) and ORR from 20 to 65% in second‐line treatment.^173^, ^174^ Final OS analysis of the phase III PROFILE 1014 trial showed that there was an improvement in OS that favored crizotinib after crossover adjustment.^175^ While crizotinib induced resistance in approximately a third of ALK‐rearranged NSCLC owing to on‐target mutation or progression in the CNS.^176^ Unlike T790M gatekeeper mutation predominating in EGFR‐mutant NSCLC, a much broader spectrum of on‐target mutations has been detected in ALK TKI‐resistant NSCLC. The subsequent development of more selective and potent second‐generation ALK inhibitors, such as ceritinib, alectinib, and brigatinib, is efficacious after crizotinib‐driven resistance, including Leu1196Met and Gly1269Ala mutations in ALK.^177^, ^178^ In second‐line therapy, nearly half of the patients resistant to crizotinib can receive a complete or PR when using second‐generation ALK inhibitors. However, they did not target all the ALK mutants resistant to crizotinib.^179^, ^180^, ^181^, ^182^ The third‐generation ALK inhibitor, lorlatinib, is effective against resistance mutations (including p.G1202r) generated by first‐ and second‐generation ALK inhibitors.^183^ When lorlatinib was used in patients with ALK‐positive NSCLC previously treated with one or more ALK kinase inhibitors, the ORR can still reach 48%.^184^

Second‐ and third‐generation ALK inhibitors are not just for post‐line therapy. Continuous researches give more choice for patients with ALK‐positive NSCLC in first‐line treatment. The first second‐generation ALK inhibitor ceritinib showed a statistically significant and clinically meaningful improvement in PFS (median PFS: 16.6 vs. 8.1 months; HR, 0.55; 95%CI: 0.42, 0.73; *p* < 0.00001) versus chemotherapy.^185^ Then, alectinib, brigatinib, and lorlatinib were demonstrated to be superior to crizotinib on 12‐month EFS rate, ORR, and intracranial response in untreated ALK‐positive NSCLC.^186^, ^187^, ^188^ For example, second interim analysis of the phase III ALTA‐1L trial showed that median PFS was 24 months with brigatinib and 11 months with crizotinib (HR, 0.49; 95%CI: 0.35, 0.68; *p* < 0.0001).^189^ In addition, alectinib, brigatinib, and lorlatinib also have potent CNS penetration to control intracranial lesions even after resistance to crizotinib.^190^ Together with ceritinib, they have been approved to treat ALK‐positive NSCLC in first‐line treatment.

As the kinase domains of ALK is closely related to that of ROS1, ALK inhibitors regularly also bind to ROS1.^191^ The US FDA has approved two ROS1 kinase TKIs, crizotinib and entrectinib, for ROS1‐positive NSCLC. As mentioned above, crizotinib also inhibits ROS1 activity.^191^ Given a dose of 250 mg twice daily, the ORR was 72%, median PFS was 19.3 months, with median OS at 51.4 months. Progression in CNS because of its poor brain penetration makes the need for new ROS1‐targeting inhibitors with better cerebral penetration urgently.^192^ Entrectinib shows similar activity against TRK, ROS1, and ALK in vitro. Entrectinib inhibits NTRK‐fusion tumors will be discussed below. In vitro, its anti‐ROS1 activity is 40 times more potent than that of crizotinib. The phase II basket STARTRK‐2 trial demonstrated the efficacy of entrectinib in ROS1‐arranged NSCLC with no prior therapy with a ROS1 inhibitor. The ORR was 67.1% and median PFS was 15.7 months in the entire population. For the 24 patients with measurable brain metastases at diagnosis, the ORR was 79.2% and median PFS was 12 months.^193^, ^194^

Many efforts have been made to broaden the indications and enhance the antitumor activity of already approved ALK/ROS1 inhibitors. Inflammatory myofibroblastic tumors (IMTs) are a kind of rare mesenchymal tumor consisting of a variable mixture of myofibroblasts and inflammatory infiltrates that can occur throughout the body, mainly in the mesentery, retroperitoneum, and pelvis. ALK rearrangements occur in ≥50% of IMTs. On July 14, 2022, the US FDA approved crizotinib for patients with unresectable, recurrent, or refractory ALK‐positive IMTs based on a phase Ib trial (NCT01121588). The ORR was 67% for ALK‐positive IMTs with a consistent safety profile, the efficacy of which was confirmed by a phase II study (NCT01524926).^195^, ^196^ In addition, a phase I–II study (NCT01970865) showed clinical activity of lorlatinib in advanced ROS1‐positive NSCLC, including those with CNS metastases and those previously treated with crizotinib.^197^ Like EGFR TKIs plus ICIs for NSCLC, it is uncertain whether ALK/ROS1 TKIs could combine with ICIs. The outcome of group E in CheckMate 370 showed that 38% (five out of 13) of patients treated with nivolumab plus crizotinib developed severe hepatic toxicities, including two deaths.^198^ Another phase Ib trial evaluated the possibility of combination of nivolumab and ceritinib and subsequently showed a potent efficacy (ORR reaching up to 83%) with increasing toxicity in the treatment of naïve ALK‐rearranged NSCLC.^199^ Further studies are needed to determine the possibility and the pattern of combination regimes.

Researches are ongoing for developing new ALK/ROS1 inhibitors. Ensartinib has been approved in China for ALK‐positive NSLCL. A phase III trial (NCT02767804) showed that first‐line treatment with ensartinib for ALK‐positive NSCLC had superior efficacy to crizotinib in both systemic and intracranial disease.^200^ A phase II study (NCT03215693) demonstrated the efficacy and safety of ensartinib in crizotinib‐resistant, ALK‐positive NSCLC.^201^ Repotrectinib belongs to fourth‐generation ALK inhibitor that targets ALK, ROS1, and TRK with similar IC50 values for ALK, ALK (G1202R), and ALK (L1196M). It is in phase II clinical trials for solid tumors with ALK, ROS1, or NTRK1‐3 rearrangements (NCT03093116). A new ALK/ROS1 inhibitor XZP‐3621 is underestimation in patients with ALK or ROS1 rearrangement NSCLC (NCT05204628). Taletrectinib is a ROS1/TRK inhibitor with potent activity against ROS1 G2032R mutation. A phase I study of this agent showed preliminary efficacy in patients with crizotinib‐refractory ROS1‐positive NSCLC with manageable toxicities.^202^ Those novel ALK/ROS1/TRK, ALK/ROS1, or ROS1/TRK will provide more chance for tumors with ALK or ROS1 alterations.

### 3.6 MET receptor kinase inhibitors

Mesenchymal–epithelial transition factor (MET), also known as the tyrosine receptor of HGF, is a single‐pass transmembrane receptor. MET homodimerization activates the MAPK and PI3K/AKT/mTOR pathways, which promote cell migration, proliferation, and survival.^203^ MET dysregulation through gene amplification and/or overexpression, mutation, and rearrangement can promote cancer initiation, progression, and malignancy in epithelial cancers.^204^ Of these, MET exon 14 skipping mutations promote oncogenic activity by suppressing MET receptor degradation and occur in 3–4% of patients with NSCLC.^203^

Two small molecule kinase inhibitors targeting MET, capmatinib and tepotinib, got accelerated approval for NSCLC with MET exon 14 skipping in 2020 and 2021, respectively. For capmatinib, the phase II GEOMETRY mono‐1 trial demonstrated that the ORR in the treatment‐naïve population was 68% and DOR was 12.6 months, with 41% and 9.7 months, respectively, in the previously treated group. At the same time, low‐grade peripheral edema and nausea were the main toxic effects.^205^ To expand the scope of capmatinib in NSCLC with MET exon 14 skipping, a phase II trial (NCT04926831) is recruiting patients to evaluate the possibility of using capmatinib in neoadjuvant and adjuvant treatment. When NSCLC with MET exon 14 skipping treated by tepotinib, the ORR was 56% and was similar between the treatment‐naïve arm and the previously treated arm.^206^

Researches on MET inhibitors are focused in three directions. First, selective MET inhibitors are explored in other types of MET‐dysregulated NSCLC and different kinds of tumors. MET amplification or overexpression attributes to one of the predominant EGFR‐TKI resistance mechanisms in patients with EGFR‐mutated NSCLC.^124^ Phase Ib/II studies evaluated the efficacy and safety of gefitinib plus capmatinib or tepotinib after the failure of EGFR‐TKI therapy in patients with EGFR‐mutated, MET‐amplified/overexpressing NSCLC, the results of which showed these combination regimes were a promising treatment, especially for patients with a MET gene copy number ≥6 or high (IHC3+) MET overexpression.^207^, ^208^ Adding MET inhibitors to the continuous medication of previous EGFR TKIs may be another method to overcome resistance to EGFR TKIs induced by MET overexpression, and many researches are ongoing (NCT04816214 and NCT03940703). In addition, MET alterations also occur in other types of tumors. A phase Ib/II trial (NCT01988493) showed that tepotinib improved TTP versus sorafenib in treatment‐naïve HCC with MET overexpression.^209^ Another phase Ib/II (NCT02115373) trial indicated that tepotinib was efficacious in sorafenib pretreated HCC with MET overexpression.^210^ Then, combination strategies are investigated to enhance efficacy. Combination of MET inhibitors with ICIs, other targeted therapies (including VEGFR inhibitors, MEK inhibitors, and HER inhibitors), and chemotherapy are ongoing. Advanced NSCLC patients with MET exon 14 skipping mutations are prepared to receive capmatinib plus spartalizumab (a new PD‐1 inhibitor) (NCT04139317). A phase I trial (NCT05435846) is ready to recruit NSCLC patients with MET exon 14 skipping mutations to receive capmatinib plus trametinib. A phase I/II trial (NCT05439993) of paclitaxel and tepotinib is recruiting patients with advanced gastric and gastroesophageal junction carcinomas with MET amplification or MET exon 14 alterations. There are still many new MET inhibitors under development. Savolitinib is a selective MET TKI and yielded promising activity in pulmonary sarcomatoid carcinomas and NSCLC with MET exon 14 skipping with ORR reaching 42.9% and an acceptable safety profile (NCT02897479), which has already been approved in China for this kind of NSCLC.^211^ Phase III trials (NCT04923945 and NCT05261399) of savolitinib for NSCLC patients with MET exon 14 mutations or NSCLC patients whose disease progressed on osimertinib are recruiting. It is also tested for RCC, gastric cancer, and esophagogastric junction adenocarcinoma (NCT05043090 and NCT04923932). Tivantinib is the first non‐ATP competitive and selective oral MET kinase inhibitor. It has been explored in various types of tumors, such as NSLCL and HCC (NCT01395758 and NCT01755767). However, phase III study of tivantinib plus erlotinib versus placebo plus erlotinib for nonsquamous NSCLC was terminated (NCT01244191). Glumetinib is also a potent and highly selective Met inhibitor. Bozitinib is a highly selective ATP‐competitive Met inhibitor with blood‐brain barrier permeability. Both of them are on their phase II stage for NSCLC with MET exon 14 mutations (NCT04270591 and NCT04258033).

### 3.7 RET receptor kinase inhibitors

Rearranged during transfection (RET) is a transmembrane RTK whose homodimerization activates several downstream pathways, including MAPK, PI3K/AKT/mTOR, JAK/STAT, PKA, and PKC pathways, essential for the normal development and maturation of diverse tissues.^203^ Aberrant RET signaling in cancers, due to RET mutations, gene fusions, and overexpression, results in the activation of downstream pathways promoting proliferation, differentiation, and survival. RET fusions occur in 1–2% of NSCLC and up to 20% of papillary thyroid cancer. RET mutations may cause multiple endocrine neoplasia 2, characterized by a high risk of developing medullary thyroid cancer (MTC).^212^, ^213^

Multikinase inhibitors with anti‐RET activity, such as cabozantinib and vandetanib, were initially tested in patients with advanced RET‐rearranged NSCLC.^214^, ^215^ In 2020, RET‐selective inhibitors pralsetinib and selpercatinib received clinical approval for RET fusion‐positive NSCLC, RET‐mutant MTC, and RET fusion‐positive thyroid cancer based on the results of the phase I/II ARROW study and the phase I/II LIBRETTO‐001 trial, respectively.^213^ In RET fusion‐positive NSCLC, the ORR of pralsetinib was 53% in patients with previous platinum‐based chemotherapy and 70% in patients who were treatment naïve with no treatment‐related deaths. Correspondingly, the ORR of selpercatinib increased to 64 and 85%, respectively. The ORRs of pralsetinib were 71 and 60%, respectively, in patients with treatment‐naive RET‐mutant MTC and in patients who had previously received cabozantinib or vandetanib. The LIBRETTO‐001 trial showed that previous use of cabozantinib or vandetanib for RET‐mutant MTC has little impact on selpercatinib‐related ORR (69 vs. 73%). In patients with previously treated RET fusion‐positive thyroid cancer, the percentages who had a response were 89% for pralsetinib and 79% for selpercatinib, respectively.^213^, ^216^, ^217^, ^218^ In addition, selpercatinib is tested in advanced solid tumors, lymphomas, and histiocytic disorders with activating RET gene alterations (NCT04320888). Both pralsetinib and selpercatinib are under verification in the approved indications (NCT05170204, NCT04194944, and NCT04211337).

Many MET inhibitors are undergoing clinical trials. RXDX‐105 is a multikinase RET inhibitor. In phase I/Ib trial for RET inhibitor‐naïve patients with RET fusion‐positive NSCLCs, RXDX‐105 showed different ORRs between KIF5B‐RET‐containing and non‐KIF5B‐RET‐containing tumors. The ORRs were 0 and 67% for these two gene alterations, respectively (NCT01877811). The reason contributing to the differential responses is not clear.^219^ TPX‐0046 is a RET/SRC inhibitor. This agent is assessed in phase I/II clinical trial (NCT04161391) for advanced solid tumors harboring RET fusions or mutations. A RET inhibitor TAS0953/HM06 is evaluated clinically to treat advanced solid tumors with RET gene abnormalities (NCT04683250). Through the above efforts, it is expected that the prognosis of RET‐altered tumors can be improved.

### 3.8 FGFR kinase inhibitors

Fibroblast growth factor (FGF) receptors (FGFR1, FGFR2, FGFR3, and FGFR4) are RTKs consisting of three extracellular immunoglobulin (Ig)‐like domains (I, II, III), a transmembrane domain and two intracellular tyrosine kinase domains (TK1 and TK2).^220^ In the presence of FGF or other ligands, FGFRs dimerization further induces the activation of downstream signaling cascades, including PI3K/AKT/mTOR, MAPK, and JAK/STAT pathways, with a role in a variety of physiological processes, such as embryonic development, metabolic homeostasis, tissue repair, and regeneration.^19^ The aberrantly activated FGF/FGFR signaling plays a pivotal role in the oncogenic process, including proliferation, survival, migration, and invasion of cancer cells, angiogenesis and immune evasion in the tumor microenvironment (TME).^220^ Multikinase inhibitors with anti‐FGFR activity often simultaneously target VEGF/VEGFR signaling, thereby collaboratively interfering with tumor angiogenesis and regulating the immune microenvironment.^19^ The details of multikinase inhibitors will be discussed below, and we focus on selective FGFR inhibitors in this part. An analysis of 4853 solid tumors by the next‐generation sequencing (NGS) technique demonstrated that FGFR aberrations occurred in 7.1% of cancers. Among them, gene amplification, gene mutations, and gene rearrangement accounted for 66, 26, and 8%, respectively. Selective FGFR inhibitors are a promising method to treat FGFR‐ altered tumors.^19^, ^221^

To date, three selective FGFR kinase inhibitors, erdafitinib, pemigatinib, and infigratinib, are approved for treating urothelial carcinoma or cholangiocarcinoma.^222^, ^223^, ^224^ Erdafitinib is a pan‐FGFR inhibitor. A phase I study (NCT01703481) showed that erdafitinib‐related response rates in urothelial carcinoma and cholangiocarcinoma were highest among advanced solid tumors with genomic changes in the FGFR pathway.^225^ In the phase II BLC2001 trial, erdafitinib brought an ORR of 40% to patients with urothelial carcinoma susceptible to FGFR3 or FGFR2 genetic alterations whose diseases progressed during or following at least one line of prior platinum‐containing chemotherapy. While treatment‐related grade 3 or higher adverse events were reported in nearly half the patients.^222^ Analysis of long‐term efficacy and safety of erdafitinib showed consistent activity and a manageable safety profile.^226^ Pemigatinib and infigratinib are FGFR1‐3 inhibitors approved for previously treated cholangiocarcinoma with an FGFR2 fusion or other rearrangements. The ORR was 35.5% in pemigatinib and 23% in infigratinib. Hyperphosphataemia was the most common all‐grade adverse event irrespective of the cause in both pemigatinib and infigratinib and more than half of patients had a grade 3 or worse adverse event, including hyperphosphatemia, stomatitis, and fatigue.^223^, ^224^ In other words, FGFR inhibitors are mildly effective but have comparatively serious side effects.

These selective FGFR inhibitors have been explored in other tumors. Preclinical trials showed that erdafitinib inhibited the growth of glioma cells with FGFR3‐TACC3 fusions in vitro and in vivo. Erdafitinib manifested clinical improvement with stable disease and minor response for glioblastoma patients with FGFR3‐TACC3 rearrangements.^227^ However, infigratinib had limited efficacy in patients with recurrent gliomas and different FGFR genetic alterations.^228^ A phase I trial (NCT01004224) demonstrated antitumor activity of infigratinib for FGFR1‐amplified squamous cell NSCLC and FGFR3‐mutant bladder/urothelial cancers.^229^ The three approved FGFR inhibitors are also under evaluation in breast cancer, bladder cancer, prostate cancer, myeloid/lymphoid neoplasms, and NSCLC and in combination with PD‐1 monoclonal antibody for advanced solid tumors (NCT03238196, NCT04917809, NCT04754425, NCT03011372, NCT05210946, and NCT03547037).

There are many selective FGFR inhibitors at different stages of clinical trials. FGFR inhibitors with clinical activity are classified into FGFR1‐4, FGFR1‐3, and FGFR4 inhibitors. Futibatinib, rogaratinib, LY2874455, and ASP5878 belong to FGFR1‐4 inhibitors. Futibatinib is an irreversible FGFR1‐4 inhibitor. Phase I trial (NCT02052778) of futibatinib recruited patients with advanced solid tumors harboring FGF/FGFR aberrations. This trial highlighted that futibatinib was also effective in patients whose disease was already resistant to prior FGFR inhibitors.^230^ Phase II trials of futibatinib for urothelial carcinoma and breast cancer are ongoing (NCT04601857 and NCT04024436). In addition, it is in phase III trial (NCT04093362) for advanced cholangiocarcinoma harboring FGFR2 gene rearrangements. Rogaratinib is a pan‐FGFR inhibitor. Phase I trial of rogaratinib (NCT01976741) showed clinically active against several types of cancer, especially for the subgroup selected by FGFR mRNA expression.^231^ A phase II/III trial (NCT03410693) of rogaratinib has completed for urothelial carcinoma. Both LY2874455 and ASP5878 are on their phase I stage (NCT03125239, NCT01212107, and NCT02038673). AZD4547 and derazantinib are potent and selective FGFR1‐3 inhibitors. AZD4547 has been clinically evaluated as a second‐line treatment in FGFR1‐amplified squamous cell NSCLC, gastric adenocarcinoma with FGFR2 polysomy or gene amplification, malignant pleural mesothelioma, the results of which showed AZD4547 was well tolerated but had minimal antitumor activity.^232^, ^233^, ^234^ Derazantinib is on phase II stage for cholangiocarcinoma (NCT03230318). Selective FGFR4 inhibitors are mainly developed to inhibit the growth of HCC. Phase I trials of H3B‐6527 and FGF401 for HCC have completed (NCT02834780 and NCT02325739). According to previous studies, we conclude that finding the specific tumor histology with FGFR alterations is essential for the efficacy of FGFR inhibitors.

### 3.9 TRK receptor kinase inhibitors

NTRK1, NTRK2, and NTR3 genes encode tropomyosin receptor kinase A, B, and C (TRKA, TRKB, and TRKC), which are the members of the cell surface RTK family, regulating cell proliferation, differentiation, and apoptosis through activate pathways of PLCγ, MAPK, PI3K/AKT/mTOR, and PKC.^235^ NTRK gene fusions lead to TRK fusion proteins with constitutive, ligand‐independent activation of the intrinsic TK. The incidence of NTRK gene rearrangements is very low across a wide range of different tumor types.^236^, ^237^, ^238^ TRK TKIs are developed based on the tumor's molecular characteristics rather than a tumor primary site.^239^

Two agents, larotrectinib and entrectinib, have received US FDA approval for NTRK‐gene fusion locally advanced or metastatic tumors. A pooled analysis of three phase I/II clinical trials (NCT02122913, NCT02637687, and NCT02576431) showed that the ORR of larotrectinib was 79% in patients with TRK fusion‐positive solid tumors, with 16% of patients having CR.^240^ Subgroup analysis of those trials yielded the rapid and durable responses and high disease control rate for patients with TRK fusion‐positive CNS tumors.^241^ An integrated analysis of three entrectinib‐related phase I/II trials (ALKA‐372‐001, STARTRK‐1, and STARTRK‐2) showed that 57% of patients had an objective response, of which 7% were CR and 50% PR and median DOR was 10 months.^193^ What is more, grade 3 or 4 adverse events of any kind for larotrectinib and entrectinib happened in less than 10% of patients.^193^, ^240^ In total, the treatment of patients with NTRK fusion‐positive cancers with larotrectinib or entrectinib is associated with high response rates and well‐tolerated.

Similarly, developing new indications for existing drugs and exploiting new drugs are two important research directions. Trials have been done or are ongoing to find new usage scenarios. A pediatric phase I trial of neoadjuvant larotrectinib presented a promising medication for children with newly diagnosed and locally advanced TRK fusion sarcomas, which might expand the use of TRK inhibitors in neoadjuvant therapy.^242^ Though larotrectinib and entrectinib have been approved for TRK fusion‐positive solid tumors, studies are ongoing to assess their efficacy and safety in histologically specific tumors, such as glioma and non‐Hodgkin lymphoma (NHL) (NCT04655404 and NCT03155620). Acquired resistance to prior TRK kinase inhibitors has spawned the development of next‐generation TRK TKI. TRKA G595R mutation was found in LMNA‐NTRK1 fusion‐positive CRC treated with larotrectinib. ETV6‐NTRK3 fusion‐positive infantile fibrosarcoma progressed on larotrectinib because of TRKC G623R mutation. Selitrectinib is a second‐generation TRK TKI and was demonstrated to inhibit these two recurrent tumors.^243^ A novel potent TRK inhibitor, AZD7451, completely blocked TRC activation and inhibited the growth of high TRKC‐expressed adenoid cystic carcinoma in the preclinical trial.^244^ Phase I trial (NCT01468324) of this agent for patients with recurrent gliomas has been completed.

### 3.10 FLT3 receptor kinase inhibitors

FMS‐like tyrosine kinase 3 (FLT3), expressed in various lymphohematopoietic cells and tissues, belongs to the class III RTK family and plays an important role in regulating cell survival, cell proliferation, and differentiation of hematopoietic progenitor cells through activating PI3K/AKT/ mTOR, MAPK, and JAK/STAT downstream signalings.^245^ Mutations of the FLT3 gene exist in about 30% of patients with AML, with 25% having the FLT3 internal tandem duplication (ITD) mutation and the other 5–10% presenting with the FLT3 tyrosine kinase domain (TKD) mutation, which lead ligand‐independent activating of the RTK and the downstream proliferative signaling pathways.^246^, ^247^

Correspondingly, FLT3 inhibitors are developed to treat FLT3‐mutated AML.^248^ Midostaurin is the first FLT3 inhibitor approved for newly diagnosed FLT3 mutation‐positive AML. The phase IIB trial of midostaurin for wild‐type or mutated FLT3 AML and high‐risk myelodysplastic syndrome (MDS) showed that the rate of reduction in peripheral blood or bone marrow blasts by ≥50% was 71% in patients with FLT3‐mutant and 42% in patients with FLT3 wild‐type. The success of the following phase III trial led to the approval of midostaurin. Using midostaurin together with standard chemotherapy regimens significantly improved patients’ EFS from 3.0 months for placebo plus standard chemotherapy to 8.2 months for midostaurin plus standard chemotherapy (HR, 0.78; 95%CI: 0.66, 0.93; one‐sided *p* = 0.002) and OS from 25.6 months to 74.7 months (HR, 0.78; 95%CI: 0.63, 0.96; one‐sided *p* = 0.009). The rate of severe adverse events was similar in the two groups.^249^, ^250^ Given the structural similarity of FLT3 and KIT, midostaurin also inhibits D816V‐mutated KIT and has been approved for aggressive systemic mastocytosis.^251^ Rapid generation of resistance mutations, particularly in codon Asp835 (D835), gave rise to second‐generation FLT3 inhibitors.^252^ Gilteritinib, a second‐generation inhibitor, is the only US FDA‐approved FLT3 inhibitor to be used as a single agent for patients with relapsed or refractory AML having an FLT3 ITD, D835, or I836 mutation based on the results of the phase III ADMIRAL trial. In this trial, gilteritinib significantly prolonged survival compared with chemotherapy (median OS: 9.3 vs. 5.6 months; HR, 0.64; 95%CI: 0.49, 0.81; *p* < 0.001). The percentages of patients with CR (21.1 vs. 10.5%) and CR with full or partial hematologic recovery (34.0 vs. 15.3%) were higher in the gilteritinib than in the chemotherapy group. In addition, grade 3 or higher and serious adverse events occurred less frequently in the gilteritinib group than in the chemotherapy group.^253^ Taken together, FLT3 inhibitors are suitable for treating FLT3‐mutated AML in first‐ and second‐line treatment because of their excellent efficacy and low toxicity.

Researches on midostaurin and gilteritinib are ongoing. Only midostaurin has been approved for first‐line treatment of FLT3 mutant AML. A phase II study (NCT03836209) is recruiting untreated patients with FLT3 mutant AML to compare the effectiveness of gilteritinib to midostaurin in first‐line therapy. Enhancing efficacy through combination therapy is another trend. A phase I/II trial for patients with AML and MDS showed that the combination of midostaurin and a DMNT inhibitor 5‐azacytidine was an effective and safe regime, especially for patients with FLT3 mutations but not previously exposed to other FLT3 inhibitors.^254^ Combining gilteritinib with the anti‐CD33 antibody gemtuzumab plus cytarabine for FLT3‐ITD‐mutated relapsed/refractory AML is in the phase II stage (NCT05199051).

New FLT3 inhibitors are under different research stages. The second‐generation FLT3 inhibitor quizartinib is widely studied and is the most promising drug for approval. FLT3 inhibitors are classified as either type I or II based on their interaction with their kinase targets. Type I FLT3 inhibitors target either FLT3‐ITD or FLT3‐TKD point mutations, while type II inhibitors commonly only target FLT3‐ITD.^248^ Quizartinib is a selective and highly potent type II FLT3 inhibitor. Phase III trial (NCT02039726) of monotherapy with quizartinib has been proved to be efficacious and safe in patients with FLT3‐ITD‐mutated, relapsed/refractory AML. OS was longer for quizartinib than for salvage chemotherapy (median OS: 6.2 vs. 4.7 months; HR, 0.76; 95%CI: 0.58, 0.98; *p* = 0.02).^255^ A phase I trial (NCT01468467) yielded that following allogeneic hematopoietic‐cell transplant with quizartinib had acceptable tolerability and reduced relapse rate.^256^ Quizartinib, combined with standard chemotherapy, is a promising treatment for newly diagnosed AML because of its safety and tolerability (NCT 01390337).^257^ Another second‐generation FLT3 kinase inhibitor, crenolanib, belongs to type I inhibitor with activity against FLT3‐ITD mutants and FLT3‐D835 point mutants.^258^ Clinical trials similar to quizartinib are also underway in crenolanib, with results not yet available or published. For example, a Phase III trial (NCT03258931) comparing crenolanib with midostaurin following induction chemotherapy and consolidation therapy is ongoing in newly diagnosed FLT3‐mutated AML. Phase II studies (NCT01657682 and NCT01522469) of crenolanib in relapsed or refractory AML with FLT3 activating mutations have been finished, and the results have not yet been published. Besides, a novel FLT3 kinase inhibitor G‐749 has antitumor activity against the FLT3 wild type and mutants, including FLT3‐ITD, FLT3‐D835Y, FLT3‐ITD‐N676D, and FLT3‐ITD/F691L and can overcome drug resistance in preclinical models.^259^ As AML cells failed to develop resistant clones under incubation with quziartinib plus the CDK4 inhibitor PD0332991, a dual FLT3/CDK4 inhibitor AMG 925 came up and was demonstrated to overcome FLT3 inhibitor resistance.^260^

### 3.11 CSF1R kinase inhibitors

The TME is rich in cytokines and their receptors, which exert complex functions in antitumor response or tumor‐promoting effect.^261^ For example, cytokines such as IL‐2, IFNα, and IFNγ are involved in the antitumor response, while TGF‐β is correlated to the lymphocyte‐deficient phenotype.^262^, ^263^, ^264^ Most antitumor treatments utilizing cytokines and their receptors show limited therapeutic efficacy, perhaps because patients with advanced‐stage disease might not be suitable for cytokine‐based therapy.^265^ Colony‐stimulating factor 1 receptor (CSF1R) is a lineage marker for monocytes and macrophages and the receptor for the growth factor CSF1. CSF1/CSF1R axis promotes survival, differentiation, and proliferation of macrophages. The high levels of macrophage infiltration in human tumors and the numerous tumor‐promoting actions of these extremely plastic immune cells have provided a rationale for CSF1R inhibitors.^266^

Tenosynovial giant cell tumor (TGCT) is a rare and locally aggressive neoplasm with overexpression of CSF1. Before 2019, surgery was standard with no approved systemic therapy. The CSF1R kinase inhibitor, pexidartinib, is indicated for patients with TGCT based on the data from the phase III ENLIVEN trial in 2019, which pioneers the way for harnessing cytokines and chemokines for cancer therapy.^267^ Overexpression of the CSF1R ligand promotes cell proliferation and pexidartinib can inhibit the proliferation of CSF1R‐dependent cell lines in vitro and in vivo.^268^, ^269^ In the clinical setting, the ORR was 39% with pexidartinib versus 0% with placebo in patients with TGCT (*p* < 0.0001). Serious adverse events occurred in 13% of patients in the pexidartinib group, including increasing levels of aspartate aminotransferase, alanine aminotransferase, and alkaline phosphatase and hypertension. In the ENLIVEN trial, hepatotoxicity was a dose limiting toxicity.^270^ To further clarify the liver toxicity, a long‐term study is ongoing to evaluate hepatotoxicity associated with pexidartinib (NCT04635111).

Pexidartinib has also been explored in other tumor types. Trials of pexidartinib in advanced castration‐resistant prostate cancer, recurrent glioblastoma, and relapsed or refractory Hodgkin lymphoma have been finished, and the results are waiting to be published (NCT01499043, NCT01349036, and NCT01217229). A similar situation is for the phase I/II trial (NCT02452424) of pexidartinib plus anti‐PD‐1 antibody pembrolizumab for advanced melanoma and some other solid tumors.

The development of novel CSF1R kinase inhibitors is relatively slow and a few monoclonal antibodies against CSF1R are also exploited to target the CSF1/CSF1R axis. Edicotinib is a selective inhibitor of the CSF1R tyrosine kinase. Phase I/II study of this agent for relapsed/refractory classical Hodgkin lymphoma demonstrated its safety profile but limited antitumor activity in treating classical Hodgkin lymphoma.^271^ Phase II trial (NCT03557970) of evaluating edicotinib in patients with AML is under the writing stage. CSF1R monoclonal antibodies conclude AMG 820 and emactuzumab. AMG 820 was tolerated with manageable toxicities up to 20 mg/kg every 2 weeks but had limited antitumor activity.^272^ Emactuzumab showed its preliminary activity against TGCT with acceptable toxicities in a phase I trial and also resulted in poor response in solid tumors (NCT01494688).^273^, ^274^

### 3.12 RAF/MEK/ERK kinase inhibitors

The MAPK cascade, also known as the RAS/RAF/MEK/ERK signaling pathway, regulates cell proliferation, differentiation, and survival.^275^ As the first component of MAPK signaling, RAS has three gene isoforms: HRAS, KRAS, and NRAS and they are small GTPases that initiate RAF/MEK/ERK kinase cascade. The RAF family includes three RAF isoforms, ARAF, BRAF, and CRAF, and is the direct downstream of RAS. RAF activates two dual‐specific kinases MEK1/2, which contain a docking site for substrate ERK1/2. Activated ERK phosphorylates a number of substrates that regulate cell functions.^276^ Alterations in key genes of this pathway can lead to a consequential constitutional activation of MAPK, inducing transformation and tumor progression.^277^ Aberrant activation of this signaling pathway plays an important role in over 40% of human cancer cases, including melanoma, NSCLC, anaplastic thyroid cancer (ATC), and other types of cancer.^278^ RAS small molecule inhibitors are not protein kinase inhibitors and will be discussed below, and none of the ERK inhibitors have been approved by the US FDA to date.^279^, ^280^ In this part, we will focus on RAF and MEK kinase inhibitors.

BRAF, MEK, and ERK are oncogenes encoding serine‐threonine protein kinases. Some mutations in the BRAF gene result in constitutively activated BRAF proteins and then triggering downstream MEK and ERK.^276^, ^277^ Up to 66% of melanomas have BRAF mutations, BRAF V600E occupying nearly 80% of total BRAF mutations, followed by BRAF V600K (20%).^281^, ^282^ The main indication for BRAF and MEK inhibitors is BRAF V600 mutant melanomas. Three BRAF inhibitors (vemurafenib, dabrafenib, and encorafenib) and three MEK inhibitors (trametinib, cobimetinib, and binimetinib) are permitted for metastatic melanoma with BRAF V600E or V600K mutations as a single agent or in combination with other agents and exhibit excellent efficacies.^283^ Three drugs (vemurafenib, dabrafenib, and trametinib) are allowed as single agents for melanoma. In first‐line therapy, compared with chemotherapy (dacarbazine or paclitaxel), they increased median PFS by at least 3 months, from less than 2 months to over 5 months, and ORR from less than 20% to more than 50%. The rates of OS were also proved to be improved with BRAF or MEK inhibitors.^284^, ^285^, ^286^

Vertical blockade of BRAF and MEK in the MAPK pathway reduces paradoxical downstream activations and efficiently targets the MAPK pathway.^282^, ^283^ Three combinations of BRAF and MEK inhibitors have been approved by the US FDA for metastatic melanoma with BRAF V600E or V600K mutations: cobimetinib plus vemurafenib, dabrafenib plus trametinib, and encorafenib plus binimetinib.^278^, ^281^ Vemurafenib was the first BRAF kinase inhibitor approved for melanoma.^287^ Combination of cobimetinib with vemurafenib prolonged median PFS from 7.2 months to 12.3 months (HR, 0.56; 95%CI: 0.46, 0.72; *p* < 0.0001) and median OS from 17.4 months to 22.3 months (HR, 0.70; 95%CI: 0.55, 0.90; *p* = 0.005) and the safety profile for cobimetinib plus vemurafenib was tolerable and manageable in the phase III coBRIM trial.^288^ Compared with dabrafenib monotherapy in the phase III COMBI‐d study or vemurafenib in the phase III COMBI‐v study, the addition of trametinib to dabrafenib was demonstrated to promote ORR and prolong PFS and OS.^289^, ^290^ The COMBI‐v study also concluded that dabrafenib plus trametinib significantly alleviated disease‐associated and adverse‐event‐associated symptoms.^290^ Furthermore, trametinib to dabrafenib is effective against melanoma brain metastases.^291^ Similarly, the phase III COLUMBUS trial illustrated benefits in terms of PFS and OS in the combination of encorafenib with binimetinib compared with vemurafenib when treating patients who were BRAF‐V600E mutant melanoma and were treatment naïve or progressed on or after first‐line immunotherapy. Median PFS increased from 7.3 months in monotherapy to 14.9 months in the doublets (HR, 0.54; 95%CI: 0.41, 0.71; *p* < 0.0001), median OS from 16.9 months to 33.6 months (HR, 0.61; 95%CI: 0.47, 0.79; *p* < 0.0001).^292^ In short, dual inhibition of BRAF and MEK to unresectable or metastatic melanoma with BRAF V600 mutation is superior to inhibiting BRAF or MEK separately in the form of ORR, PFS, OS, and quality of life. At the same time, combination therapy could reduce toxicities by avoiding paradoxical downstream activations.^293^ Besides, adjuvant treatment with BRAF inhibitor dabrafenib and MEK inhibitor trametinib is approved in resected stage III melanoma with BRAF V600E or V600K mutations. Five‐year follow‐up of phase III COMBI‐AD trial demonstrated that 12 months of adjuvant therapy with dabrafenib plus trametinib resulted in a longer duration of relapse‐free survival and survival without distant metastasis.^294^

As mentioned above, other types of cancer may harbor BRAF mutations or MAPK pathway aberrations. Correspondingly, monotherapy with BRAF or MEK inhibitors and BRAF plus MEK inhibitors also have indications in other types of tumors. Vemurafenib is indicated for the treatment of patients with BRAF V600 mutant erdheim‐chester disease.^295^ Selumetinib, a new MEK inhibitor, broadens indication to neurofibromatosis type 1 with inoperable plexiform neurofibromas and the ORR reached 66% in the SPRINT phase II Stratum 1 trial.^296^ Dabrafenib in combination with trametinib is approved for advanced or metastatic BRAF V600E mutant NSCLC and ATC with BRAF V600E mutation.^297^, ^298^ The US FDA recently granted accelerated approval to dabrafenib in combination with trametinib for unresectable or metastatic solid tumors with BRAF V600E mutation after standard therapies.^299^

Though having not been permitted, many clinical trials have proven efficacy of BRAF or MEK inhibitors or BRAF plus MEK inhibitors in more cancer types. Phase II clinical trials showed that vemurafenib represented a potential new treatment option for BRAF‐V600E‐positive papillary thyroid cancer and NSCLC with BRAF V600 mutations.^300^, ^301^ Trametinib prolonged PFS of patients with recurrent low‐grade serous ovarian cancer compared with standard of care.^302^ Patients with BRAF V600E‐mutant glioma and BRAF V600E‐mutated biliary tract cancer benefited from the dabrafenib plus trametinib based on the results of the phase II ROAR trial.^303^, ^304^

However, BRAF or MEK inhibitors are not suitable for all BRAF‐mutant cancers. Phase II clinical trials showed that single‐agent vemurafenib did not show meaningful clinical activity in patients with BRAF V600E mutant‐CRC, which may attribute to tumor specificity and feedback mechanisms and may be conquered by combination strategies.^305^ Fortunately, the phase III BEACON study showed BRAF inhibitor encorafenib plus EGFR antibody cetuximab improved OS, ORR, and PFS compared with standard chemotherapy and is a new standard of care for previously treated BRAF V600E‐mutant metastatic CRC.^306^

The clinical benefit of combining PD‐1/PD‐L1 antibody with RAF and MEK inhibitors is inconsistent. Preclinical trials provide a rationale for these combining strategies.^307^ The phase III IMspire150 trial showed that treating BRAF V600‐mutant melanoma with atezolizumab, vemurafenib, and cobimetinib significantly increased PFS from 10.6 months with vemurafenib and cobimetinib to 15.1 months (HR, 0.78; 95%CI: 0.63, 0.97; *p* = 0.025) without obviously adding adverse events.^308^ However, the phase III COMBI‐i trial evaluated spartalizumab plus dabrafenib and trametinib for BRAF V600‐mutant melanoma, the results of which showed that the addition of spartalizumab to targeted therapy with dabrafenib and trametinib did not significantly prolong PFS but increased grade ≥3 adverse events by 22% compared with the dabrafenib plus dabrafenib group.^309^ Further details and trials are waiting to clarify this combination's possibility.

Novel kinase inhibitors involved in RAF, MEK, and ERK are under development. New RAF inhibitors are developed to resolve more molecular alterations in RAS‐RAF‐MEK signaling. Belvarafenib is a type II RAF inhibitor and is effective in BRAF V600E‐ and NRAS‐mutant melanoma.^310^ Mutations in ARAF contributed to resistance to belvarafenib, which could be addressed by combination strategies. Related experiments are executed in NRAS‐mutant melanoma and solid tumors (NCT04835805 and NCT03284502). Another RAF inhibitor, lifirafenib, is a reversible inhibitor of BRAF V600E, wild‐type ARAF, BRAF, CRAF, and EGFR and showed antitumor activity in BRAF V600‐mutated solid tumors (NCT02610361).^311^ Further trials may compare lifirafenib with first‐generation BRAF inhibitors and explore the possibility of lifirafenib as second‐line therapy (NCT03905148). Research on new MEK drugs is not always successful. The study on MEK inhibitor CI‐1040 has been halted because of its insufficient antitumor activity.^312^ Monotherapy with a second‐generation MEK inhibitor, mirdametinib, is not recommended.^313^ Trials are ongoing as combination regimes (NCT03905148 and NCT03170206). Refametinib is an allosteric MEK inhibitor and yields preliminary efficacy in some kinds of solid tumors, such as HCC and pancreatic cancers.^314^, ^315^ A phase II study (NCT01204177) showed that HCC patients with RAS mutations could benefit from refametinib/sorafenib combination with a DCR of 44.8%.^314^ However, follow‐up studies of this drug are lacking. Dual inhibition of RAF and MEK is a trend. RO5126766, also known as CH5126766 or VS‐6766, is a dual RAF/MEK inhibitor and has antitumor activity across various cancers with RAF‐RAS‐MEK pathway mutations.^316^ A combination of RO5126766 with PD‐1/PD‐L1 antibody may increase the antitumor effect.^317^ The development of selective ERK inhibitors lags far behind compared with the RAF and MEK inhibitors. The clinical trials of ERK inhibitors are in their early stages, such as ulixertinib, GDC0994, and LY3214996. Ulixertinib showed potent preclinical activity in BRAF‐ and RAS‐ mutant cell lines and had clinical activity in NRAS‐ and BRAF V600‐ and non‐V600‐mutant solid‐tumor malignancies with an acceptable safety profile in the phase I trial (NCT01781429).^318^ Phase II studies of this agent involved in solid tumors, NHL, AML, and MDS are under‐evaluated (NCT03155620, NCT04488003, NCT02296242, and NCT03155620). GDC0994 is another selective ERK inhibitor and only has been tested in phase I trials. The single‐agent activity was observed in BRAF‐mutant CRC (NCT01875705).^319^ The combination of cobimetinib and GDC‐0994 led to cumulative toxicity, which restricted further development (NCT02457793).^320^ LY3214996 is a potent and selective ATP‐competitive inhibitor of ERK with IC50 values for ERK1and ERK2 below 0.001 mmol/L.^321^ Clinical trials of this agent are ongoing (NCT02857270, NCT04616183, and NCT04956640).

### 3.13 PI3K/AKT/mTOR kinase inhibitors

The PI3K/AKT/mTOR signaling pathway triggered by various extracellular stimuli regulates cell proliferation, survival, and angiogenesis. PI3Ks are the family of vital lipid kinases widely distributed in mammalian cells that phosphorylate PIP2 into PIP3, which activates PDK1 and then influences AKT. Activation of PI3K and AKT leads to mTOR activation and phosphorylation of S6K1 and 4e‐BP1.^322^ Among the three classes of PI3K, class I is the most important, with four isoforms (PI3Kα, PI3Kβ, PI3Kδ, and PI3Kγ), the catalytic isoforms of which are encoded by PIK3CA, PIK3CB, PIK3CG, and PIK3CD, respectively. PI3Kα and PI3Kβ are ubiquitously expressed in various tissues. PI3Kδ expression is limited mainly to the B cells and their precursors, whereas PI3Kγ is expressed in T lymphocytes.^323^, ^324^ Hyperactivation of the PI3K/AKT/mTOR pathway in tumors provides a rationale to target key elements involved in this cascade.^325^, ^326^ AKT and mTOR kinase inhibitors are in clinical trials.^327^ Rapamycin and its analogs (temsirolimus and everolimus) are allosteric inhibitors of mTOR will be discussed below.^328^ Here, we focus on PI3K kinase inhibitors and briefly introduce AKT and mTOR kinase inhibitors.

It is reported that PIK3CA mutations occur in 40% of HR‐positive, HER2‐negative breast cancer.^329^, ^330^ The selective PI3Kα inhibitor alpelisib combined with fulvestrant is permitted to treat breast HR‐positive, HER2‐negative breast cancer harboring PIK3CA mutation after an endocrine‐based regimen. The phase III SOLAR‐1 trial demonstrated that the PFS and ORR of the alpelisib plus fulvestrant arm were better than those of the placebo plus fulvestrant arm (median PFS: 11.0 vs. 5.7 months, HR, 0.65, 95%CI: 0.50, 0.85, *p* < 0.001; ORR: 35.7 vs. 16.2%).^331^ Acquired of PTEN may attribute to resistance of alpelisib, which could be reversed by PTEN knockdown.^332^ Several critical phase II trials have received positive results concerned with PI3Kα inhibitor alpelisib. Monotherapy with alpelisib in later lines of therapy for PI3K‐altered, pretreated advanced cancer received an ORR of 30%.^333^ In another phase II study, alpelisib plus fulvestrant proved their antitumor activity and manageable toxicity in patients with PI3KCA‐mutated, HR‐positive, HER2‐negative advanced breast cancer after progression on a CDK4/6 inhibitor plus an aromatase inhibitor, which offered a choice for these kinds of patients (NCT03056755).^334^ As PI3Kδ is widely expressed in B cells and their precursors, idelalisib, copanlisib, duvelisib, and umbralisib are PI3Kδ inhibitors approved for the post line therapy of leukemia or lymphoma derived from B cells, including chronic lymphocytic leukemia (CLL)/small lymphocytic lymphoma (SLL), follicular lymphoma (FL), and marginal zone lymphoma (MZL).^335^, ^336^, ^337^, ^338^ The standard of care for patients with relapsed or refractory CLL is bendamustine plus rituximab. Idelalisib plus CD20 antibody rituximab for patients with relapsed CLL whose disease only suitable for rituximab alone received 19.4 months of median PFS and 81% of ORR, while the values were 6.5 months and 13%, respectively, for the placebo plus rituximab group.^339^ Final observation showed that OS favored the patients in the idelalisib plus rituximab group (median OS: 40.6 vs. 34.6 months; HR, 0.8; 95%CI: 0.5, 1.1; *p* = 0.1343).^340^ Good results from clinical trials resulted in the approval of idelalisib plus rituximab for CLL. Besides, adding idelalisib to bendamustine plus rituximab prolonged median PFS from 11.1 months in the bendamustine plus rituximab group to 20.8 months (HR, 0.33; 95%CI: 0.25, 0.44; *p* < 0.0001) (NCT01569295). In contrast, the accompanying serious adverse events and infections may prevent its approval. Compared with the PI3Kδ specific inhibitor idelalisib, the PI3Kδ/γ dual duvelisib shows a more potent inhibitory activity of PI3K protein and the US FDA has approved duvelisib for CLL or SLL after at least two prior therapies.^341^ In addition, the phase II DYNAMO study showed that duvelisib monotherapy may provide a new option for patients with heavily pretreated, double‐refractory indolent NHL. The ORR was 47.3%, which even reached 67.9% for SLL.^342^ Another PI3Kα/δ dual inhibitor, copanlisib, was granted US FDA accelerated approval in 2017 for FL after at least two prior systemic therapies based on an ORR of 59% under monotherapy with copanlisib in the phase II CHRONOS‐1 trial.^343^ In addition, the phase III CHRONOS‐3 trial supported the combination of copanlisib with rituximab for relapsed indolent NHL, in which the median PFS was increased by 7.7 months compared with rituximab monotherapy.^344^ Umbralisib is a selective PI3Kδ inhibitor indicated for MZL and FL and reduces the incidence of autoimmune complications as it additionally inhibits CK1ε.^345^, ^346^ Except for copanlisib, the other four PI3K‐approved inhibitors are orally bioavailable.^335^ However, the incidence of grade 3 or 4 adverse events is much higher in the PI3K inhibitor group than in the placebo group. The role of the PI3K/AKT/mTOR signaling pathway in the immune system renders inhibitors bring significant on‐target toxicities, such as pneumonitis, stomatitis, and infections, which also limit their use in the clinic.^347^

Though several PI3K inhibitors have been approved, efforts never stop to exploit more suitable inhibitors. Buparlisib and pictilisib belong to pan‐PI3K kinase inhibitors, which have been tested as a single agent or combination regimes in many tumor types, such as breast cancer, squamous cell carcinoma of the head and neck, glioblastoma, and lymphoma.^348^, ^349^, ^350^ Preclinical and early‐stage clinical trials have demonstrated the safety profile and preliminary activity of monotherapy with pan‐PI3K inhibitors in some tumor types, especially for breast cancer.^351^, ^352^, ^353^ However, two phase III trials (BELLE‐2 and BELLE‐3) did not support buparlisib plus fulvestrant for further development in breast cancer because of toxicities.^354^, ^355^ Taselisib is a selective PI3Kα inhibitor and yields antitumor activity toward PIK3CA‐mutant tumors, especially for breast cancer.^356^ Phase II LORELEI trial found that neoadjuvant letrozole plus taselisib for oestrogen receptor‐positive, HER2‐negative, early‐stage breast cancer increased the proportion of patients who achieved an objective response.^357^ But phase III SANDPIPER trial showed the addition of taselisib to fulvestrant for oestrogen receptor‐positive, HER2‐negative, PI3KCA‐mutant, advanced breast cancer was not suggested given its toxicities and modest clinical benefit.^358^ GSK2636771 is a PI3Kβ inhibitor. The safety profile was acceptable, but the efficacy in metastatic castration‐resistant prostate cancer was limited.^359^ More clinical trials are ongoing (NCT04439188 and NCT02951091).

To date, no AKT inhibitors have been approved. Several AKT inhibitors are in different clinical stages for various tumor types, especially for breast cancer, prostate cancer, and RCC, such as ipatasertib, capivasertib, afuresertib, and MK‐2206.^360^, ^361^ Their toxicities are acceptable as a single agent or in combination with other agents. The activation of the PI3K/AKT/mTOR signaling pathway caused by gene mutation and/or deletions of PTEN is associated with drug sensitivity.^362^ For example, ipatasertib is a selective ATP‐competitive inhibitor of AKT and has received positive results in treating triple‐negative breast cancer and prostate cancer.^362^, ^363^ Notably, the only published phase III clinical trial for AKT inhibitor is ipatasertib for previously untreated metastatic castration‐resistant prostate cancer. In this trial, ipatasertib plus abiraterone significantly improved PFS compared with placebo plus abiraterone among patients with PTEN‐loss tumors. Many trials are ongoing, and four out of five phase III trials are for breast cancer.^363^ The phase III IPATunity130 is evaluating ipatasertib plus paclitaxel as first‐line therapy for PI3KCA/AKT/PTEN‐altered triple‐negative breast cancer or HR‐positive, HER2‐negative breast cancer. Another phase III trial of ipatasertib in combination with atezolizumab and paclitaxel for triple‐negative breast cancer is ready to recruit. Two phase III trials (NCT04060862 and NCT04650581) are for HR‐positive or ER‐positive, HER2‐negative breast cancer. MK‐2206 is an allosteric inhibitor of AKT and resulted in a higher pathologic complete response rate in HR‐negative and HER2‐positive breast cancer when combined with standard neoadjuvant therapy (NCT01042379).^364^ However, it was not superior to standard RCC and pancreatic cancer treatment.^365^, ^366^

The approved mTOR inhibitors only inhibit TORC1, which could inversely activate AKT. mTOR kinase inhibitors can inhibit TORC1 and TORC2 and are supposed to avoid resistance caused by AKT phosphorylation.^328^ Currently, no mTOR kinase inhibitors are approved, but some are under evaluation, such as AZD2014, sapanisertib, and vistusertib.^367^, ^368^, ^369^ Notably, vistusertib plus anastrozole improved was superior to anastrozole alone in HR‐positive, recurrent or metastatic endometrial cancer in the form of PFS and ORR with manageable adverse events.^369^ Dual inhibition of PI3K and mTOR is a direction, and many dual PI3K/mTOR inhibitors are in their early‐stage trials, such as gedatolisib, paxalisib, samotolisib, voxtalisib, and apitolisib.^370^ For example, samotolisib is a promising dual PI3K/mTOR inhibitor and had a clinical benefit on PFS in metastatic castration‐resistant prostate cancer with tolerable side effects when combined with enzalutamide.^371^ Trials on advanced solid tumors, NHL, and histiocytic disorders are ongoing (NCT03155620 and NCT03213678). Paxalisib is a brain‐penetrant dual PI3K/mTOR inhibitor and was proved to cross the blood‐brain barrier in phase I clinical trial (NCT01547546).^372^ Further trials of paxalisib are undergoing in tumors with primary and secondary brain metastases, including glioblastoma and breast cancer with brain metastases (NCT03970447, NCT05009992, and NCT03994796). Dual inhibition is supposed to achieve better antitumor activity than inhibiting one mechanism alone but also increases toxicity. Phase II trial (NCT01442090) comparing apitolisib with everolimus in RCC failed to show better outcomes due to multiple on‐target adverse events.

### 3.14 JAK kinase inhibitors

The Janus kinase (JAK)/signal transducer and activators of transcription (STAT) pathway plays essential roles in tumorigenesis and immune function by mediating the cellular response to cytokines, interferons, and growth factors.^373^ JAKs belong to the intracellular nonreceptor protein tyrosine kinase family and consist of four isoforms (JAK1‐3 and TYK2).^374^ The STAT protein family comprises seven members, including STAT1‐6, STA5a, and STA5b.^375^ Upon ligands binding to the surface receptors, dimerization of JAK‐associated receptors induces activation of JAK kinases, which in turn recruit and phosphorylate cytosolic STAT proteins and lead to nuclear translocation of STATs, which function as transcription factors. Mutation or amplification of JAK1, JAK2, STAT3, and STAT5 frequently occurs in malignant tumors, which induces dysregulated JAK/STAT signaling and therefore provides targets for tumor treatment.^376^

Myelofibrosis is a kind of hematological malignancy that includes primary myelofibrosis, postpolycythaemia vera myelofibrosis, and postessential thrombocytosis myelofibrosis, and ectopically activates the JAK/STAT pathway.^377^, ^378^ Hyperactivation of JAK/STAT pathway provides a rationale for targeting JAK or STAT in myelofibrosis. Two JAK inhibitors are approved for myelofibrosis. Ruxolitinib, a selective JAK1/JAK2 inhibitor, significantly alleviates palpable splenomegaly, ameliorated debilitating myelofibrosis‐related symptoms, and improved OS compared with placebo. The primary endpoint was the proportion of patients with a reduction in spleen volume of 35% or more at 24 weeks. 41.9% of patients in the ruxolitinib group achieved a 35% or greater reduction in spleen volume from baseline, while less than 1% of patients had such curative effect (*p* < 0.001).^379^, ^380^, ^381^ Fedratinib, a JAK2‐selective inhibitor, has a similar role in controlling palpable splenomegaly when treating myelofibrosis.^382^, ^383^ Though JAK inhibitors have also been trialed and received mild response in patients with chronic neutrophilic leukemia, atypical CML, and metastatic pancreatic cancer, none of them are formally permitted.^384^, ^385^ Except for cancer, the JAK/STAT pathway also has impact on immunity, JAK inhibitors are therefore used in treating autoimmune diseases, which are beyond our discussion and will not be described in our review.^386^ Adverse events related to JAK inhibitors include anemia, thrombocytopenia, gastrointestinal symptoms, increased levels of liver transaminases, serum creatinine, and pancreatic enzymes. Among these, serious and fatal encephalopathy caused by fedratinib received a black‐box warning indicating that reliable prevention and monitoring strategies are needed during the medication process.^381^, ^383^

Several JAK inhibitors have been explored, but some of them discontinued further research due to serious adverse events or limited efficacy, such as JAK1/2 inhibitor AZD1480 and momelotinib. AZD1480 ceased in the phase I study because of unusual dose‐limiting toxicities and the lack of clinical activity.^387^ Momelotinib failed in two published III trials for the treatment of myelofibrosis. The phase III SIMPLIFY‐1 trial of momelotinib versus ruxolitinib in JAK inhibitor‐naïve myelofibrosis demonstrated that 24 weeks of momelotinib treatment was noninferior to ruxolitinib for spleen response but not for symptom response.^388^ The phase III SIMPLIFY‐2 trial of comparing momelotinib with the best available therapy in treating myelofibrosis previously treated with ruxolitinib showed that momelotinib was not superior to the best available therapy.^389^ Luckily, pacritinib is a promising JAK2/JAK2 (V617F) inhibitor and has received positive outcomes in clinical trials. Two phase III trials (PERSIST‐1 and PERSIST‐2) showed that pacritinib was well tolerated and induced significant and sustained spleen volume reduction and symptom reduction, even in patients with thrombocytopenia or resistance to ruxolitinib.^390^, ^391^ Phase II study of this agent for prostate cancer is ongoing (NCT04635059). Itacitinib is a JAK1 inhibitor and has shown preliminary antitumor activity and safety profile in early‐stage trials for B‐cell lymphoma and pancreatic cancers.^392^ Now it is under evaluation in many clinical trials for various tumor types, such as myelofibrosis, HCC, NSCLC, sarcoma, Hodgkin lymphoma, T‐cell prolymphocytic leukemia, and diffuse large B‐cell lymphoma (NCT04640025, NCT04358185, NCT02917993, NCT03670069, NCT03697408, NCT03989466, and NCT02760485).

### 3.15 CDK kinase inhibitors

Cell cycle is crucial for cell proliferation process and mainly mediated by cyclin‐dependent kinases (CDKs), cyclins, and CDK inhibitors. CDKs are the cell cycle activators and require cyclin proteins for phosphorylation of the key substrates. CDK inhibitors negatively regulates the activity of CDKs. In the human cell, at least 20 CDKs and 29 cyclins have been identified.^393^ Complexes of CDK1‐cyclin A/B, CDK2‐cyclin E/A, and CDK4/CDK6‐cyclin D are required in phase progression, completion of which is verified by three checkpoints at G1‐S, G2‐M, and metaphase‐to‐anaphase transitions.^394^ Increased expression levels of CDKs or cyclins, or decreased endogenous CDK inhibitors’ levels, have been observed in cancers. CDKs are protein‐serine/threonine kinases and have become a target for anticancer therapy based on their role in cell proliferation.^395^

To date, the CDK4/6 inhibitors have been proved to interfere with the proliferation of breast cancer cells by decreasing pRb phosphorylation and arresting the cell cycle in the G1 phase.^396^ Since 2015, three CDK4/6 inhibitors, palbociclib, ribociclib, and abemaciclib have been approved for HR‐positive/HER2‐negative breast cancer in various settings and combination regimens.^397^, ^398^ The addition of the CDK4/6 inhibitors palbociclib, ribociclib, and abemaciclib to an aromatase inhibitor were studied in postmenopausal HR‐positive/HER2‐negative advanced breast cancer in the first‐line setting in PALOMA‐2, MONALEESA‐2, and MONARCH‐3 trials, respectively. The three combination regimens nearly doubled the duration of median PFS and improved ORR compared with the single‐use of an aromatase inhibitor, with or without prolonged OS.^399^, ^400^, ^401^ In second‐line treatment, CDK4/6 inhibitors combined with the selective estrogen receptor degrader fulvestrant for HR‐positive/HER2‐negative breast cancer were studied in three trials: PALOMA‐3, MONALEESA‐3, and MONARCH‐2. The phase III trials showed the CDK4/6 inhibitors prolonged the PFS with or without OS.^402^, ^403^, ^404^ Besides, monotherapy with abemaciclib was also permitted for HR‐positive/HER2‐negative advanced breast cancer after prior endocrine therapy and 1–2 chemotherapy regimens according to an ORR of 19.7% in the MONARCH‐1 trial.^405^ The regime of abemaciclib plus standard endocrine therapy can be used as adjuvant treatment for postmenopausal, high‐risk, and HR‐positive/HER2‐negative early breast cancer.^406^ However, CDK4/6 inhibitors largely increased the rates of myelotoxic effects, especially for neutropenia and leukopenia. Fortunately, neutropenia and leukopenia are relatively easy to manage in clinic.^398^ In 2021, the newest CDK4/CDK6 inhibitor, trilaciclib, was permitted for patients with SCLC indicated to prevent chemotherapy‐induced myelosuppression.^407^ SCLC is an ideal disease model to assess the myeloprotective effect of trilaciclib. First, chemotherapy for SCLC is highly hematologically toxic and SCLC is a chemosensitive tumor, providing a chance to demonstrate that trilaciclib does not antagonize chemotherapy efficacy. Second, SCLC tumor cells replicate independently of CDK4/6 and trilaciclib will not directly have effects on the tumor.^408^ Trilaciclib administered before chemotherapy could transiently maintain hematopoietic stem and progenitor cells in G1 arrest and protect them from damage by cytotoxic chemotherapy, leading to faster hematopoietic recovery and enhanced antitumor immunity.^407^

In addition to the approved indications, these CDK4/6 inhibitors are also trying to broaden their use scopes. The phase II PALLET trial evaluated adding palbociclib to letrozole as neoadjuvant therapy in patients with ER‐positive early breast cancer. However, this regime did not increase the clinical response rate over 14 weeks.^409^ Phase II trials supported the use of this agent in HPV‐unrelated HNSCC and liposarcoma (NCT02101034 and NCT01209598).^410^, ^411^ Phase II trial of ribociclib in combination with docetaxel plus prednisone had acceptable toxicity and encouraging efficacy in metastatic castration‐resistant prostate cancer.^412^ Abemaciclib even showed promising clinical activity in patients with p16ink4A‐deficient mesothelioma in a single‐arm phase II trial (NCT03654833).^413^ These inhibitors are ongoing clinical trials for ovarian cancer, NSCLC, RCC, AML, and so on (NCT03936270, NCT03170206, NCT05468697, and NCT03844997).

New CDK4/6 inhibitors are still in development. Dalpiciclib is also a CDK4/6 inhibitor and has been approved in combination with fulvestrant for HR‐positive and HER2‐negative breast cancer progressing on endocrine therapy in China.^414^ Lerociclib is a novel CDK4/CDK6 inhibitor with IC50 values in the nanomolar range.^415^ It showed an antiproliferation role in ER‐positive breast cancer in animal models and is under clinical evaluation in treating breast cancer and NSCLC (NCT05085002 and NCT03455829). PF‐06873600 is a CDK4/6 and CDK2 inhibitor, which may overcome resistance to CDK4/6 inhibition.^416^ Phase II study (NCT03519178) of this agent for cancers in recruiting. In addition to CDK4/CDK6 inhibitors, inhibitors of other CDK isoforms are emerging. Dinaciclib is a CDK1/2/5/9 inhibitor with the values of IC50 below 4nM. It has shown preliminary efficacy and acceptable toxicity in treating CML in early‐stage trials and a phase III trial for CML has been completed (NCT01580228).^417^ Fadraciclib is a second‐generation inhibitor of CDK2/9, with IC50s of 5nM and 26nM, respectively, and two phase I/II trials (NCT04983810 and NCT05168904) for solid and hematological tumors are ongoing.^418^ CDKI‐73 is a CDK9 inhibitor, which is CDKI‐73 has been studied a lot in preclinical in recent years and may be a promising agent.^419^

### 3.16 BTK tyrosine inhibitors

Bruton's agammaglobulinemia tyrosine kinase (BTK) belongs to the Tec non‐RTK family, which is one of the largest kinase families in mammals and includes BTK, ITK, TEC, BMX, and TXK.^420^ BTK is also a key cytoplasmatic kinase in the B cell antigen receptor signal transduction pathway and regulates various signals, such as PI3K/AKT/mTOR, MAPK, and NF‐κB pathways.^421^ As these signaling pathways regulate the proliferation, differentiation, and apoptosis of B cells, the secretion of proinflammatory cytokines, as well as degranulation and histamine release, BTK inhibition is an essential method in treating B‐cell tumors and B‐cell immune diseases with expected effect and low toxicity.^422^ In our article, we focus on tumor‐related indications.

In 2013, a first‐generation BTK inhibitor, ibrutinib, was first approved by the US FDA for mantle cell lymphoma (MCL), CLL/SLL with or without 17p deletion, waldenström's macroglobulinemia (WM), and MZL.^421^ Ibrutinib irreversibly and covalently binds to Cys481 within the ATP‐binding pocket of BTK. The ORR achieved at least 60% in the second‐line treatment and can be beyond 80% in the first‐line therapy using ibrutinib alone, which was increased after a longer follow‑up.^422^ Combination approaches of ibrutinib with other drugs (mostly BCL‐2 inhibitor venetoclax or CD20 antibody rituximab or obinutuzumab or standard chemoimmunotherapy further explored clinical benefits. The aims of combination regimes are to induce CR for minimal residual disease and then enable treatment discontinuation.^421^ Ibrutinib's administration largely improved ORR, PFS, and even OS compared with prior standardized therapies.^423^, ^424^, ^425^, ^426^, ^427^ Notably, ibrutinib represents an important therapeutic advance for the treatment of CLL/SLL.^428^, ^429^ However, ibrutinib could also bring side effects, such as bleeding, diarrhea, rash, infection, atrial fibrillation, and even ventricular arrhythmias and sudden cardiac death caused by simultaneously inhibiting the activity of other kinases in the Tec family and/or several non‐BTK kinases, including c‐terminal Src kinase, HER2, and JAK3.^430^ In addition, mutations in BTK (C481S, C481R, C481F, and C481Y) and PLCG2 (R665W, L845F, and S707Y) region confer resistance to ibrutinib. Therefore, there is a need to develop the next‐generation BTK inhibitors to overcome drug resistance, improve the selectivity of BTK, and reduce drug toxicity.^420^ Under such circumstances, second‐generation BTK inhibitors with more selectivity and more potent binding ability in BTK, acalabrutinib and zanubrutinib, were launched in the market in 2017 and 2019, respectively.^430^ Acalabrutinib was approved for CLL/SLL based on the phase III ELEVATE TN trial for treatment‐naïve CLL/SLL and the phase III ASCEND trial for relapsed or refractory CLL/SLL, both of which demonstrated prolonged PFS in the acalabrutinib group compared with the traditional treatment group.^431^, ^432^ Direct comparison of acalabrutinib and ibrutinib in patients with previously treated CLL/SLL further verified that acalabrutinib received noninferior PFS with fewer cardiovascular adverse events.^433^ The other indication of acalabrutinib is MCL, according to 81% of patients achieving an ORR treated by acalabrutinib alone. The indications for zanubrutinib include MCL, WM, and MZL.^434^, ^435^ Though not approved for CLL/SLL, the recent results of the phase III SEQUOIA trial proved that zanubrutinib significantly improved PFS versus bendamustine–rituximab and is a potential new treatment option for untreated CLL/SLL.^436^ Similar to the relationship between acalabrutinib and ibrutinib, zanubrutinib treatment was associated with a better response rate and less toxicity when compared with ibrutinib in treating patients with WM.^437^

Tirabrutinib and orelabrutinib also belong to second‐generation BTK inhibitors. Tirabrutinib binds to BTK with an IC50 of 2.2nM. The data from phase II trials indicated favorable efficacy of tirabrutinib in patients with WM and primary CNS lymphoma.^438^, ^439^ This agent has completed evaluation for previously treated CLL/SLL or NHL in a phase II study (NCT01659255). Orelabrutinib has already received approval in China for patients with MCL or CLL/ SLL who have received at least one prior treatment.^440^ The binding of first‐ and second‐generation agents to BTK rely on Cys481. Mutations at Cys481 disrupt the covalent binding between BTK and BTK inhibitors, which further induce resistance.^430^

In contrast to the covalent inhibitors, the third‐generation BTK inhibitors, pirtobrutinib and nemtabrutinib, noncovalently and reversibly bind to BTK kinase, do not rely on Cys481, and may further overcome the resistance caused by previous BTK inhibitors.^420^ Pirtobrutinib was demonstrated to circumvent resistance caused by C481S and C481R. Besides, this agent's phase I/II study (NCT03740529) showed it was safe and active in multiple B‐cell malignancies, even for patients previously treated with covalent BTK inhibitors.^441^, ^442^ Pirtobrutinib is under phase III clinical trials for multiple B‐cell malignancies (NCT05023980, NCT04965493, and NCT04662255). Compared with pirtobrutinib, nemtabrutinib is in early‐stage clinical trials for B‐cell malignancies (NCT04728893 and NCT05458297).

### 3.17 IDH tyrosine inhibitor

Isocitrate dehydrogenase 1 (IDH1) and isocitrate dehydrogenase 2 (IDH2) are important metabolic enzymes that catalyze isocitrate to α‐ketoglutarate (αKG).^443^ Mutations in the enzymatic active site of IDH1 and IDH2 block the cycle reaction of isocitrate to αKG but trigger the conversion of αKG to 2‐hydroxyglutarate (2HG). 2HG inhibits αKG‐dependent dioxygenases involved in DNA and histone methylation and is thought to block cellular differentiation to promote leukemogenesis and tumorigenesis.^444^ Mutations in IDH1 and IDH2 can be detected in various human cancers, including AML (20%), cholangiocarcinoma (20%), chondrosarcoma (80%), and glioma (80%).^445^, ^446^ Mutations in IDH1 and IDH2 occur at conserved arginine residues within the enzymatic active site, such as IDH1 R132 and IDH2 R140 or R172. Therefore, mutated IDH as a therapeutic target stirs great interest in cancer treatment research. IDH inhibitors may effectively prevent the 2HG oncometabolite, subsequently reversing epigenetic dysregulation and restoring normal cellular differentiation.

To date, two IDH inhibitors have been approved for AML and/or cholangiocarcinoma.

Enasidenib is an IDH2 inhibitor approved by the US FDA for relapsed or refractory AML with IDH2 mutation in 2017. At 100 mg daily dose of enasidenib, 19% of patients achieved CR and CR with partial hematological recovery (CRh) in another 4% of patients in a phase I/II study (NCT01915498).^447^, ^448^ A phase III study (NCT02577406) is ongoing to verify the efficacy. In the following phase II trial (NCT02677922) for newly diagnosed, IDH2‐mutant AML ineligible for intensive chemotherapy, the addition of enasidenib to azacitidine significantly improved ORR (74 vs. 36%) without adding serious treatment‐related adverse events compared with azacitidine monotherapy.^449^ But enasidenib has not been granted for newly diagnosed AML. Phase I/II trials of enasidenib for other tumor types, such as IDH2‐mutated MDS, glioma, or angioimmunoblastic T‐cell lymphoma with an IDH2 mutation, and advanced hematologic malignancies with an IDH2 mutation, are under evaluation (NCT03744390, NCT02273739, and NCT01915498). The indications for the IDH1 inhibitor, ivosidenib, extend to IDH1 mutant AML and IDH1 mutant cholangiocarcinoma. Ivosidenib, given at a dose of 500 mg/daily as a single agent for newly‐diagnosed AML with an IDH1 mutation, demonstrated CR and CRh rates of 28.6 and 14.3%, respectively. The rates of CR and CRh were 24.7 and 8.0% in relapsed or refractory IDH1 mutant AML. Specially, ivosidenib in combination with azacitidine has been approved for newly diagnosed AML with an IDH1 mutation. The phase III AGILE trial for newly diagnosed IDH1‐mutated AML ineligible for intensive induction chemotherapy showed the combination of ivosidenib and azacitidine was superior to placebo and azacitidine in terms of EFS (HR, 0.33; *p* = 0.002) and OS (median OS: 24.0 vs. 7.9 months; HR, 0.44; 95%CI: 0.27, 0.73; *p* = 0.001). CR was 47% in the ivosidenib plus azacitidine arm and 15% in the placebo plus azacitidine arm. The other indication of ivosidenib is for the treatment of locally advanced or metastatic, chemotherapy refractory cholangiocarcinoma with an IDH1 mutation based on data from the phase III study AG120‐C‐005 (ClarIDHy). Ivosidenib at a dose of 500mg daily significantly improved median PFS (HR, 0.37; 95%CI: 0.25, 0.54; *p* < 0.0001) and resulted in a favorable OS benefit (median OS: 10.3 vs. 7.5 months; HR, 0.79; 95%CI: 0.56, 1.12; one‐sided *p* = 0.09) compared with placebo.^450^, ^451^ In addition, phase I studies of ivosidenib also showed clinical benefits in IDH1‐mutated advanced glioma and advanced mutant IDH1 chondrosarcoma. Ivosidenib is trying to expand to solid and other hematological tumors with an IDH1 mutation, such as glioma, pancreatic adenocarcinoma, and myedysplastic syndrome (NCT02073994, NCT05209074, and NCT03839771). Two phase II studies (NCT04056910 and NCT04044209) are evaluating the possibility of combining ivosidenib with PD‐1 inhibitor nivolumab for IDH1‐mutated tumors.

Research on drug sensitivity and resistance found several factors were associated with the IDH inhibitors. First, cooccurring RAS pathway mutations decreased the possibility of responding to ivosidenib monotherapy. Interestingly, patients with a JAK2 mutation achieved a higher percentage of a CR or CRh.^452^ Second, approximately 30% of patients had mutations in transcription factors and/or chromatin regulators, such as RUNX1, CEBPA, GATA2, DNMT3A, and ASXL1, which were associated with acquired relapse.^453^ Third, second‐site mutations in IDH1 (i.e., D279N and S280F) and IDH2 (i.e., Q316E and I318M) interfere with the binding of the IDH inhibitors to the enzymes attributed to the therapeutic resistance.^444^

Additional small molecule IDH inhibitors are under various preclinical and clinical development stages. IDH‐305, BAY‐1436032, and DS‐1001b belong to IDH1 inhibitors. In the phase I trial (NCT02381886) for IDH1‐R132‐mutant AML and MDS, IDH‐305 exhibited a narrow therapeutic window, and the following trials were hindered.^454^ Phase I studies (NCT02746081 and NCT03127735) of BAY‐1436032 for IDH1‐mutant solid tumors and AML demonstrated it was well tolerated and showed durable ORR in lower grade glioma.^455^ But the low ORR did not support further clinical development of this agent in AML.^456^ DS‐1001b showed antitumor activity toward recurrent gliomas and chondrosarcoma in xenograft models, and clinical trials are ongoing (NCT04458272 and NCT03030066).^457^, ^458^ IDH2 inhibitor AGI‐6780 has not yet advanced to the clinical stage and was demonstrated to induce cellular differentiation in leukemia cells.^459^ Vorasidenib is a dual IDH1/IDH2 inhibitor and showed preliminary antitumor activity in recurrent or progressive nonenhancing lower grade gliomas with good tolerance (NCT02481154).^460^ Phase I study (NCT05484622) of vorasidenib and pembrolizumab combination in recurrent or progressive enhancing IDH‐1 mutant astrocytomas is ready to recruit.

### 3.18 Src tyrosine inhibitor

Nine structurally similar nonreceptor protein TKs (Src, Fyn, Lyn, Yes, Blk, Lck, Fgr, and Yrk) constitute the Src family kinases, which interact with multiple tyrosine kinase, growth factor, integrin, and G‐protein‐coupled receptors and participate in pathways promoting cell proliferation and survival.^461^ Src is not a primary driver of tumorigenesis and mutations in Src are very rare. Thus, Src inhibitors may exert an auxiliary role in cancer treatments with limiting efficacy when used alone.^462^, ^463^

The selective Src inhibitor was not approved until 2 years ago for patients with actinic keratosis, a precancerous lesion of invasive cutaneous squamous cell carcinoma (iCSCC). Actinic keratosis may progress to iCSCC at a rate of 0.025–16% per lesion per year. Because of its unpredictable nature of progression, treatment is recommended. Cryosurgery is a common method for individual actinic keratosis lesions. Photodynamic therapy and topical agents (fluorouracil, diclofenac, imiquimod, and ingenol mebutate) are recommended for multiple lesions and surrounding solar‐damaged skin. However, local reactions or long‐term medication may reduce adherence and undermine treatment success. Hence, there is a need to find other methods for actinic keratosis. In 2020, tirbanibulin, a Src‐specific kinase and tubulin polymerization inhibitor, was approved for the topical treatment of actinic keratosis based on the data from two phase III trials.^464^ In trial 1, Src inhibition induced a significant difference in the rates of complete clearance between the tirbanibulin and vehicle arm (44 vs. 5%) after 5 consecutive days of treatment when evaluating at Day 57. The percentages were 54 and 13%, respectively, in trial 2. However, the rate of recurrence of lesions at 1 year was 47% among patients who had a complete response to tirbanibulin. The most common local reactions were erythema and flaking or scaling. But it is safe to use tirbanibulin, as severe local reactions were infrequent among tirbanibulin‐treated patients.^465^ Taken together, the Src inhibitor tirbanibulin is of mild efficacy, low toxicity, and short‐term medication for treating actinic keratosis.

Combined with other agents to increase other agents’ efficacy or avoid resistance caused by the principal agents, maybe a future for selective Src inhibitors and some studies have shown the possibilities in preclinical models. Saracatinib is a selective Src inhibitor, and it was well tolerated in patients with advanced solid malignancies in the phase I trial. In the follow‐up single‐drug studies, no good results were obtained.^466^ In ER‐positive ovarian cancer models, combined Src and ER blockade by saracatinib and fulvestrant inhibited ovarian cancer xenograft growth more effectively than monotherapy.^467^ Dual Src and MEK inhibition in ovarian cancer models addressed bypass activation.^468^ Attenuating Src kinase activity could increase the efficacy of poly‐ADP‐ribose polymerase (PARP) inhibitors in BRCA2‐altered prostate cancer cells.^469^ However, it failed to improve the activity of weekly paclitaxel in platinum‐resistant ovarian cancer and increase the efficacy of a VEGF‐targeted therapy in patients with relapsed metastatic clear RCC in clinical settings.^470^, ^471^ More clinical trials are needed to verify the possibility of combination.

Selective small molecule kinase inhibitors are supposed to inhibit a single protein kinase, but they usually bind to a few structurally related kinases.^10^ This feature brings two obvious clinical benefits, enhancing their efficacy for particular cancers and indicating for cancers harboring different molecular alterations.^6^ For example, targeting both CDK4 and CDK6 by palbociclib, ribociclib, abemaciclib, and trilaciclib is believed to increase the effectiveness in treating breast cancer.^397^ In addition, entrectinib, which is a TRK receptor kinase inhibitor indicated for NTRK fusion‐positive tumors, is also a potent inhibitor of ROS1, enabling it to be approved for ROS1‐positive NSCLC.^193^, ^194^, ^472^ On the other hand, off‐target toxicity deserves attention.^1^ As mentioned above, serious cardiovascular side effects induced by poor selectivity of first‐generation BTK inhibitor ibrutinib could be reduced by second‐generation BTK inhibitor acalabrutinib or zanubrutinib because of their high selectivity to BTK.^430^

## 4 SELECTIVE SMALL MOLECULE NONKINASE INHIBITORS

Selective small molecule nonkinase inhibitors bind to targets beyond the kinome, thereby blocking subsequent functions to control tumors.^10^ This part will first introduce the molecular targets involved in the nucleus, whose mechanisms are gene transcription, DNA repair, epigenetic modification, and nuclear protein exportation. Next, receptor and intracellular signaling inhibitors will be described. Finally, you can find agents involved in triggering apoptosis (Table 2 and Table S2).

**TABLE 2:** Summary of approved small molecule nonkinase inhibitors

Class	Drug name	Company	First approval	Targets	Administration pathway	Indications
HIF	Belzutifan (Welireg)	Merck	2021	HIF	Oral	VHL‐associated RCC, CNS hemangioblastomas and pNET
PARP	Olaparib (Lynparza)	AstraZeneca	2014	PARP1, PARP2, PARP3	Oral	Ovarian, fallopian tube or primary peritoneal cancer, breast cancer, pancreatic cancer, prostate cancer
PARP	Rucaparib (Rubraca)	Clovis Oncology	2016	PARP1, PARP2, PARP3	Oral	Ovarian cancer, prostate cancer
PARP	Niraparib (Zejula)	GlaxoSmithkline	2017	PARP1, PARP2	Oral	Ovarian cancer
PARP	Talazoparib (Talzenna)	Pfizer	2018	PARP1, PARP2	Oral	Breast cancer
DNMT	Azacitidine (Vidaza)	Celgene	2004	DNMT	Subcutaneous or intravenous	Myelodysplastic syndromes, AML, JMML
DNMT	Decitabine (Dacogen)	Otsuka	2006	DNMT	Intravenous	Myelodysplastic syndromes
HDAC	Vorinostat (Zolinza)	Merck	2006	HDAC1, HDAC2, HDAC3, HDAC6	Oral	cutaneous T‐cell lymphoma
HDAC	Romidepsin (Istodax)	Celgene	2010	HDACs	Intravenous	Cutaneous T‐cell lymphoma
HDAC	Belinostat (Beleodaq)	Acrotech	2014	HDACs	Intravenous	Peripheral T‐cell lymphoma
HDAC	Panobinostat (Farydak)	Secura	2015	HDACs	Oral	Multiple myeloma
XPO1	Selinexor (Xpovio)	Karyopharm Theraps	2019	XPO1	Oral	Multiple myeloma, diffuse large B‐cell lymphoma
CXCR4	Plerixafor (Mozobil)	Dr Reddys Labs	2008	CXCR4	Subcutaneous	Autologous transplantation for NHL and MM
KRASG12C	Sotorasib (Lumakras)	Amgen	2021	KRASG12C	Oral	NSCLC with KRAS G12C‐mutation
mTOR	Temsirolimus (Torisel)	PF Prism CV	2007	mTOR	Intravenous	RCC
mTOR	Everolimus (Afinitor)	Novartis	2009	mTOR	Oral	HR‐positive, HER2‐negative breast cancer, neuroendocrine tumors, RCC,
mTOR	Sirolimus (albumin‐bound) (Fyarro)	DR Reddys Labs	2021	mTOR	Intravenous	Perivascular epithelioid cell tumor
SMO	Vismodegib (Erivedge)	GenenTech	2012	SMO	Oral	(Locally advanced)/metastatic basal cell carcinoma
SMO	Sonidegib (Odomzo)	Sun Pharm	2015	SMO	Oral	Local advanced basal cell carcinoma
SMO	Glasdegib (Daurismo)	Pfizer	2018	SMO	Oral	AML
BCL‐2	Venetoclax (Venclexta)	Abbvie	2016	BCL‐2	Oral	CLL/SLL, AML

Abbreviations: RCC, renal cell carcinoma; pNET, pancreatic neuroendocrine tumor; NHL, non‐Hodgkin's lymphoma; MM, multiple myeloma; AML, acute myeloid leukemia; CLL/SLL, chronic lymphocytic leukemia/small lymphocytic lymphoma; JMML, juvenile myelomonocytic leukemia. Data sources: https://www.fda.gov/drugs/development‐approval‐process‐drugs/drug‐approvals‐and‐databases.

### 4.1 Hypoxia‐inducible factor 1α inhibitors

Hypoxia‐inducible factor 1α (HIF‐1α) and HIF‐2α trigger HIF‐1 transcription to promote induction of various proangiogenic genes, such as VEGF, VEGFR, EGF, ANGPT, Tie‐2, TIMP‐1, and PAI‐1, and thereby play critical roles in tumor proliferation, apoptosis, metabolism, and metastasis in response to low‐oxygen concentrations in the TME, which can be hydrolysis by VHL protein.^473^ HIF‐1 translation is also modulated by PI3K/AKT/mTOR, MAPK, JAK/STAT, NF‐Kβ, and Ca2+/CaM pathways in the cytoplasm. HIF‐1 accumulation in VHL‐associated tumors is a therapeutic target in cancer therapy.^474^, ^475^

Four strategies can be used for the interference of HIF‐1 accumulation. Direct inhibition of HIF‐1 transcription by HIF‐1α or HIF‐2α inhibitor is an important method. Blocking HIF‐1 translation or signaling pathways associated with HIF‐1 collection and enhancing HIF‐1 degradation can also circumvent the roles of HIF‐1.^474^ In 2021, the HIF‐2α inhibitor belzutifan was approved as monotherapy for patients with VHL‐associated RCC, CNS hemangioblastomas, and pancreatic neuroendocrine tumor (pNET) based on the basket phase II trial (NCT03401788) showing an ORR of 49, 63, and 83%, respectively. The most common adverse events were anemia and fatigue. Meanwhile, embryo‐fetal toxicity deserves attention.^476^

Clinical studies of belzutifan (as monotherapy or combination therapy) for particular tumor types are ongoing, especially for RCC and pNET. Among them, 12 out of all 18 trials are for RCC. A phase III study (NCT04195750) is ready to recruit 736 participants with RCC to compare belzutifan with everolimus. Combination of belzutifan with PD‐1 inhibitor and/or multikinase inhibitor (lenvatinib or cabozantinib) is a trend to increase efficacy and avoid resistance and is under estimation in several phase II or III trials (NCT05239728, NCT04976634, and NCT03634540).

The factors affecting the above four aspects of HIF‐1 may impact HIF‐1 accumulation. There are also HIF‐1α inhibitors under study, such as IDF‐11774 and PX‐478. IDF‐11774 is a HIF‐1α inhibitor that suppresses HSP70 chaperone activity to disrupt HIF‐1α refolding and stimulate HIF‐1α degradation. It showed antitumor activity on melanoma, prostate cancer, and thyroid cancer in vitro and/or in vivo.^477^, ^478^ PX‐478 is also an inhibitor of constitutive and HIF‐1α levels and thus HIF‐1 activity. It showed antitumor activity in various cancer types and could synergize with anti‐PD‐1 to impair tumor growth in preclinical trials.^479^, ^480^ A phase I trial (NCT00522652) of this agent for solid tumors has been completed, but the results have not been published.

### 4.2 PARP inhibitors

PARP contributes to DNA damage repair (DDR) and the maintenance of genomic stability, which allow cancer cells to develop resistance to radiation and DNA damaging chemotherapeutics.^481^ BRCA1/2 and ATM are tumor suppressors that are commonly mutated or dysregulated in breast or ovarian cancers. Tumors with BRCA, ATM, or other mutations have a deficiency in homologous recombination, which repairs the DNA double‐strand breaks, DNA and protein‐DNA cross‐links, and collapses replication forks, and thereby induces genomic instability. PARP protein is overexpressed in various types of cancers, such as breast, ovarian, and oral cancers compared with their normal surrounding healthy tissues. PARP inhibitors are employed to disturb the DDR and stall DNA replication to trigger apoptosis in cancers (also called synthetic lethality), specifically for cancers with homologous recombination deficiency (HRD), which refers to tumor BRCA, ATM, or other mutant, or a genomic instability score ≥42.^482^, ^483^ Therefore, inhibition of PARP activity will become a promising strategy for cancer therapy. Four PARP inhibitors (olaparib, rucaparib, niraparib, and talazoparib) have been licensed to date for use in the treatment of ovarian, breast, pancreatic cancer, and prostate cancer. PARP inhibitors for ovarian and breast cancer are studied more than other tumors.^482^

Three PARP inhibitors (olaparib, rucaparib, and niraparib) for ovarian cancer can be used as a single agent during maintenance therapy after response to first‐line platinum‐based chemotherapy or as monotherapy in the post‐line treatment.^484^, ^485^, ^486^, ^487^ In the maintenance setting, three pivotal phase III trials (SOLO‐2, NOVA, and ARIEL) demonstrated PFS was significantly prolonged in the PARP inhibitor group than in the placebo group (median PFS: 16.6–21.0 months vs. 5.4–5.5 months). Although BRCA mutations or HRD are not required for rucaparib and niraparib, subgroup analyses showed longer PFS in patients with these changes.^486^, ^487^ Monotherapy with rucaparib or niraparib is suitable for BRCA‐mutated or HRD‐positive ovarian cancer in post line therapy. The ORR ranged from 24% after 3 or more chemotherapies with niraparib to 54% after 2 or more chemotherapies with rucaparib. The main common adverse events of these drugs in ovarian cancer were nausea, fatigue, vomiting, and anemia.^488^, ^489^, ^490^

In breast cancer, olaparib and talazoparib demonstrated significant clinical benefits over chemotherapy in PFS and ORR for germline BRCA‐mutated, HER2‐negative metastatic or locally advanced breast cancer based on the phase III OlympiAD and EMBRACA trials, respectively. Notably, grade ≥3 adverse events in the olaparib arm were fewer than in the chemotherapy arm (36.6 vs. 50.5%). Compared with chemotherapy, talazoparib brought a 17% increase in grade 3–4 hematologic adverse events and a 6% decrease in the rate of nonhematologic grade 3 events.^491^, ^492^ In addition, olaparib was also approved for the adjuvant treatment of patients with germline BRCA‐mutated, HER2‐negative metastatic breast cancer after chemotherapy on the basis of better iDFS and OS compared with placebo.^493^

In addition, two PARP inhibitors, olaparib and rucaparib, received US FDA designation for metastatic castration‐resistant prostate cancer with homologous recombination repair gene mutation or BRCA mutation or ATM mutation.^494^, ^495^ The phase III PROfound trial recruited men with metastatic castration‐resistant prostate cancer who progressed on a hormonal agent, such as enzalutamide or abiraterone. Cohort A included the patients with at least one alteration in BRCA1, BRCA2, or ATM. In these individuals, olaparib was associated with longer PFS (median PFS: 7.4 vs. 3.6 months; HR, 0.34; 95%CI: 0.25, 0.7; *p* < 0.001) and OS (median OS: 19.1 vs. 14.7 months; HR, 0.69; 95%CI: 0.50, 0.97; *p* = 0.02) compared with enzalutamide or abiraterone. Anemia and nausea were the main toxic effects in patients who received olaparib.^496^ The phase II single‐arm trial TRITON2 showed an ORR of 44%, with 59% of patients occurring grade 3–4 adverse events.^497^ The phase III trial TRITON3 is ongoing to verify the clinical benefit of this agent (NCT02975934). In addition, olaparib was also approved for BRCA‐mutated pancreatic cancer in the maintenance setting after response to platinum‐based chemotherapy based on improved PFS and ORR compared with placebo.^498^

The approved PARP inhibitors are developing in two main directions. First, the therapeutic scope of PARP inhibitors is expanding to other tumor types, most with BRCA or ATM mutations, such as endometrial, urothelial, SCLC, STS, mesothelioma, and gastric cancer.^499^, ^500^ Take mesothelioma as an example. A phase II study (NCT03654833) yielded that the rate of patients with BAP1‐negative or BRCA1‐negative heavily treated mesothelioma who received disease control from rucaparib treatment at 12 weeks was 58%, and the ratio was 23% at 24 weeks.^501^ Second, combination with other agents is a promising method to increase efficacy. Chemotherapy, other targeted agents, and ICIs are the three major categories of combination partners. Phase II study (NCT01063517) of olaparib plus paclitaxel could improve OS compared with placebo plus paclitaxel in recurrent or metastatic gastric cancer, with a greater OS benefit in ATM low patients.^502^ Combined with VEGFR inhibitors is a deserving attempt. Phase III trial (NCT02477644) of adding olaparib to bevacizumab as first‐line maintenance for HRD‐positive ovarian cancer provided a significant PFS survival compared with bevacizumab alone.^503^ ICIs are another worthy companion. In the phase I/II MEDIOLA trial, the combination of olaparib with PD‐L1 inhibitor durvalumab showed promising antitumor activity and safety profile in patients with germline BRCA‐mutated metastatic breast cancer.^504^ Besides, a phase Ib/II trial (NCT03404960) demonstrated that 6‐month PFS of niraparib plus CTLA‐4 antibody ipilimumab was superior to that of niraparib plus PD‐1 antibody nivolumab for platinum‐sensitive advanced pancreatic cancer.^505^

New PARP inhibitors are ongoing in different clinical stages, such as, pamiparib, fluzoparib, iniparib, veliparib, and stenoparib.^482^ Among them, pamiparib and fluzoparib are the most representative, and they are all licensed in China. Pamiparib displays excellent PARP‐1 and PARP‐2 inhibition with IC50 of 1.3 and 0.9 nM, respectively. It is approved for the treatment of germline BRCA‐mutant recurrent advanced ovarian cancer based on a phase I/II trial (NCT03333915). This agent showed an ORR of 68.3% in the platinum‐sensitive cohort and 31.6% in the platinum‐resistant cohort.^506^ Phase III maintenance treatment trial (NCT03519230) with pamiparib versus placebo for platinum‐sensitive recurrent ovarian cancer is ongoing. Fluzoparib is a PARP‐1 inhibitor with an IC50 of 1.46 nM. The indication of fluzoparib is the same as pamiparib. The ORR reached 69.9%, with 4.4% of patients achieving a CR and 65.5% with a PR.^507^ Besides, the recently published phase III FZOCUS‐2 trial (NCT03863860) supported fluzoparib as maintenance therapy in patients with platinum‐sensitive, recurrent ovarian carcinoma, regardless of germline BRCA 1/2 mutation.^508^

Two other questions about PARP inhibitors are also important. First, the factors associated with the sensitivity of PARP inhibitors are studied extensively. As mentioned above, patients whose tumors harbor BRCA or ATM or other mutations or HRD are likely to respond to PARP inhibition. An HRD phenotype is associated with genomic scars and mutational signatures, which can be used for defining broader populations that may benefit from DDR‐targeted drugs. Moreover, there is a strong association between HRD and ovarian cancer platinum sensitivity. Therefore, platinum sensitivity functions as a surrogate marker for HRD.^483^ Second, ways to address resistance are under research. After the initial response to PARP inhibitors, tumors often develop drug resistance by developing compensatory mechanisms or restoring HR function that allows the cancer cell to repair the damage and proliferate. Combination therapy may address the bypassing activated signaling. Approximately 450 proteins that sense, signal, and/or repair DNA damage are involved in the DDR, such as MLH1, CHK1/2, WEE1, and RAD51. Therefore, inhibition of other proteins in the DDR may overcome resistance caused by PARP inhibitors.^509^

### 4.3 Epigenetic inhibitors

Tumor cells are not only activated by genetic alterations but also often use epigenetic processes to ensure their survival despite of various interference. Since epigenetic pathways exhibit greater flexibility of several orders of magnitude relative to genetic alterations, epigenetic alterations are receiving increasing attention.^510^ Epigenetics defines the regulation of chromatin structure and gene expression through modifying histone proteins and nucleic acids that regulate chromatin structure without affecting the underlying DNA sequence.^511^ Dysregulation of the epigenome drives aberrant transcriptional programs that promote cancer onset and progression, allowing for suppression of tumor suppressor genes and the increased expression of oncogenes, which leads to the development of targeted epigenetic drugs for the treatment of cancer.^512^, ^513^ Although epigenetic therapy has taken a long time to be accepted and proved effective in treating blood and solid tumors, recent drug discovery efforts have increasingly focused on DNA and histone modification. The DNA methyltransferase (DNMT) and histone deacetylase (HDAC) inhibitors are the most clinically advanced epigenetic therapies.^514^, ^515^

#### 4.3.1 DNMT inhibitors

DNA methylation is one of the best‐described epigenetic events that controls gene expression and is dysregulated in diseases like cancer.^516^ Regulation of DNA methylation depends on the family of DNMTs, including DNMT1, DNMT2, and DNMT3. DNMTs are a class of enzymes that transfer methyl groups from S‐adenosylmethionine to cytosine bases of CpG dinucleotides in gene promoters and regulatory regions.^517^ CpG promoter islands can be methylated during development, leading to long‐term gene silencing. DNA hypermethylation is also associated with silencing tumor suppressor genes and differentiation genes in various cancers.^518^ DNMT inhibitors, commonly known as hypomethylating agents, are the most widely used epigenetic therapies for cancer treatment. The DNMT inhibitors are mainly divided into two categories: (i) cytosine analog inhibitors and (ii) non‐nucleotide analog inhibitors.^519^ As a classical anticancer drug, DNMT inhibitors have been in clinical trials for more than 40 years with little effect. However, in the past 20 years, they have been revitalized due to the discovery of their mechanism of action.^520^ DNMT inhibitors mainly exert anticancer activity through the following mechanisms: (I) hypomethylation, (II) induction of double stranded DNA breaks, (III) cell cycle or G2 phase arrest, and (IV) stimulation of immune signals.^521^ To date, the US FDA approved two DNMT inhibitors, namely azacytidine and decitabine.^522^

Azacytidine is the first approved DNMT inhibitor for MDS, AML, and juvenile myelomonocytic leukemia (JMML). The phase III cancer and leukemia group B trial for higher‐risk MDS showed azacytidine treatment enhanced treatment response (60 vs. 5%, *p* < 0.001), delayed time to transformation to AML or death (21 vs. 13 months, *p* = 0.007), and improved quality of life compared with supportive care.^523^, ^524^ Moreover, OS was increased with azacitidine treatment relative to conventional care in another phase III trial (NCT00071799).^525^ In the following studies, it was found that treatment with azacitidine could substantially delay hematological relapse in measurable residual disease‐positive patients with MDS or AML who were at high risk of relapse.^526^ Patients with lower‐risk MDS could also benefit from azacitidine. The results of a phase III trial (NCT01566695) showed azacitidine significantly improved RBC transfusion independence and induced durable bilineage improvements. In addition to MDS, many efforts have been devoted to treating AML. Azacitidine was granted approval for maintenance therapy of AML ineligible for complete intensive curative therapy in 2020 based on the phase III QUAZAR AML‐001 trial. Azacitidine treatment favored in OS (median OS: 24.7 vs. 14.8 months; *p* < 0.001) and RFS (10.2 vs. 4.8 months; *p* < 0.001) compared with placebo.^527^ Azacitidine for elderly patients’ AML significantly prolongs OS (median OS: 24.5 vs. 16.0 months; HR, 0.47; 95%CI: 0.28, 0.79; *p* = 0.005) compared with conventional care regimes.^528^ Besides, azacitidine in combination with BCL‐2 inhibitor venetoclax or IDH inhibitor ivosidenib has been approved for AML, which has been discussed in other parts of this review.^529^ Failure also exists in the development process. TP53 inhibitor eprenetapopt combined with azacytidine for TP53‐mutated MDS and AML was reported to be safe and superior to azacytidine alone in the form of ORR, CR rate, and OS in a phase II trial (NCT03588078). However, it failed in the phase III trial.^530^ The newest indication of azacitidine is monotherapy before allo‐HSCT in pediatric patients with newly diagnosed JMML. The data from the phase II AZA‐JMML‐001 trial showed azacitidine provided clinical benefit to JMML patients prior to HSCT in terms of clinical CR or PR.^531^

Similarly, decitabine was licensed for MDS based on several studies demonstrating an ORR of 49% in elderly patients with high‐risk MDS, reduction of AML transformation, and improvements in patient‐reported quality of life.^532^ Besides, as the medication of decitabine via intravenous is inconvenient, the phase II ADOPT trial provided an alternative dosing schedule that can be administered in an outpatient setting with comparable efficacy and safety.^533^ Decitabine for the treatment of AML has been researched extensively. A phase II study of decitabine investigated the efficacy and toxicity of decitabine as initial therapy in older patients with AML showing an ORR of 25%, median OS of 7.7 months, and 7% of 30‐day mortality.^534^ A phase III trial compared decitabine with treatment choice in older patients with newly diagnosed AML and showed an improvement in response rates with decitabine compared with standard therapies (17.8 vs. 7.8%; HR, 2.5; 95%CI: 1.4, 4.8; *p* = 0.001). Moreover, a phase II study (ChiCTR‐IIR‐16008182) found that rhG‐CSF combined with minimal‐dose decitabine maintenance after allo‐HSCT can reduce the incidence of relapse in patients with high‐risk AML.^535^ Decitabine has also been studied in solid tumors, but only refined in phase I stage.^536^

Several aspects concerned with DNMT inhibitors deserve attention, including toxicity, efficacy, and combination therapy. First, the most common grade 3–4 adverse event was cytopenia, including neutropenia and thrombocytopenia. Clinically, there are corresponding drugs for myelosuppression, so it is not difficult to address. Second, several factors may affect the efficacy of DNMT inhibitors. Unfavorable‐risk cytogenetic abnormalities and TP53 mutations were correlated with favorable clinical response and robust (but incomplete) mutation clearance in AML and MDS treated by decitabine.^537^ We also observed that poor efficacy was seen in cases of solid tumors, primarily as single agents, including the following reasons: (i) the function of DNMT inhibitors depends upon DNA incorporation, while solid tumors divide relatively slowly; (ii) lower stability of DNMT inhibitors. Third, research on the combination of DNMT inhibitor with PD‐1 inhibitor provides a novel strategy for cancer treatment. DNMT inhibitors could upregulate PD‐1 and IFNγ signaling. Phase II trial showed azacitidine plus nivolumab brought an ORR of 33% for relapsed or refractory AML, which was 58 and 22% for hypomethylating agent‐naïve and hypomethylating‐pretreated patients, respectively.^538^ The addition of decitabine to PD‐1 antibody camrelizumab in patients with relapsed or refractory classical Hodgkin lymphoma who were clinically naïve to PD‐1 antibody received a higher CR rate than camrelizumab alone (71 vs. 32%; *p* = 0.003).^539^

Many other DNMT inhibitors are under preclinical or different clinical stages, such as guadecitabine, zebularine, and CP‐4200. The latter two drugs are in the preclinical phase.^514^ Specifically, guadecitabine is a decitabine analog and showed antitumor activity toward MDS, AML, peripheral T‐cell lymphoma, urothelial bladder carcinoma, ovarian cancer, and melanoma in clinical settings.^511^ The safety profile and efficacy of guadecitabine were initially demonstrated in MDS and AML (NCT01261312).^540^, ^541^ Even phase III trial (NCT02348489) for AML has been completed. Combination strategies broaden the use scope. Guadecitabine, in combination with ICI pembrolizumab or ipilimumab, resulted in a durable clinical benefit and reversed previous resistance to ICIs in solid tumors (NCT02998567).^542^, ^543^ Guadecitabine combined with standard chemotherapy enhanced antitumor immunity in platinum‐resistant ovarian cancer, urothelial carcinoma, and germ cell cancer.^544^, ^545^

#### 4.3.2 HDAC inhibitors

Proteins of the histone family are conserved alkaline proteins in eukaryotes and play pivotal roles in DNA packaging and gene regulation. Histone acetylation is associated with active transcription and is mainly localized at enhancer regions, promoters, and the gene body.^546^ The positively charged histone is neutralized after adding a negatively charged acetyl group to a histone tail, which further weakens the electrostatic interaction between histones and the negatively charged DNA, resulting in an open conformation of chromatin that is more accommodating to transcription factors. HDAC is an enzyme that inhibits the process of histone acetylation.^547^ At least 18 human HDACs have been identified, with varying function, localization, and substrates, and further classified into four classes of HDACs, namely class I, II, III, and IV. Classes I, II, and IV contain a zinc molecule in their active site and are inhibited by HDAC inhibitors. In the contrary, class III HDACs do not have a zinc molecule and are not hindered by any current HDAC inhibitors.^514^ Overexpression of HDAC is found in various cancers associated with the invasiveness and migration of cancer and is an indicator of poor prognosis.^548^ Inhibition of HDAC can reverse the process of histone acetylation and induce open chromatin conformation at tumor suppressor gene loci and thereby inducing tumor suppression through cell‐cycle arrest, apoptosis, and chemo/radio‐sensitization.^549^

To date, four HDAC inhibitors (vorinostat, romidepsin, belinostat, and panobinostat) have been approved to treat hematological malignancies.^550^ Vorinostat and romidepsin received US FDA approval for patients with cutaneous T‐cell lymphoma after two or one systemic therapy, respectively. The phase IIb trial (NCT00091559) showed the vorinostat‐related ORR was 29.5%, median TTP was 4.9 months, and 32% of patients had pruritus relief. The most common adverse events were diarrhea, fatigue, nausea, and anorexia.^551^ Romidepsin was approved based on a phase II trial, which demonstrated that the ORR of romidepsin was 34% and the median DOR was 13.7 months with pruritus improvement in 43% of patients. Drug‐related adverse events were generally mild, including nausea, vomiting, fatigue, transient thrombocytopenia, and granulocytopenia.^552^ However, adding romidepsin to CHOP (the standard first‐line treatment of peripheral T‐cell lymphoma) as first‐line treatment for cutaneous T‐cell lymphoma did not improve PFS, response rates, and OS but increased adverse events.^553^ Belinostat brought an ORR of 25.8% to patients with relapsed or refractory peripheral T‐cell lymphoma. The median DOR, PFS, and OS were 13.6, 1.6, and 7.9 months, respectively. The most common grade 3–4 adverse events were anemia, thrombocytopenia, dyspnea, and neutropenia.^554^ Based on the above data from the phase II study, the US FDA approved belinostat for relapsed or refractory peripheral T‐cell lymphoma. Finally, panobinostat is a potent oral pan‐deacetylase inhibitor and was licensed for multiple myeloma in combination with bortezomib and dexamethasone. The phase III PANORAMA1 trial showed that the addition of panobinostat to bortezomib and dexamethasone prolonged the median PFS from 8.08 months in the placebo, bortezomib, and dexamethasone arm to 11.99 months in the panobinostat, bortezomib, and dexamethasone arm (HR, 0.63; 95%CI 0.52, 0.76; *p* < 0.0001) when treating patients with relapsed or refractory multiple myeloma.^555^

HDAC inhibitors as a single agent or a part of combination regimes have been explored in other solid and hematological tumors, such as NSCLC, gastrointestinal carcinoma, malignant pleural mesothelioma, neuroblastoma, AML, MDS, and so on.^514^ High‐quality published research mainly revolves around the first approved selective HDAC inhibitor, vorinostat. The efficacy of monotherapy with HDAC inhibitor vorinostat was assessed in relapsed or refractory indolent NHL and MCL in a phase II study (NCT00253630). Only patients with FL and MZL responded to vorinostat, with no formal responders among patients with MCL.^556^ Monotherapy with vorinostat given as a second‐line or third‐line treatment also failed in the phase III VANTAGE‐014 trial for advanced malignant pleural mesothelioma. Vorinostat treatment did not prolong OS compared with placebo (median OS: 30.7 vs. 27.1 weeks; HR, 0.98; 95%CI: 0.83, 1.17; *p* = 0.86).^557^ Considering the limitation of vorinostat monotherapy, vorinostat combined with other agents is widely studied. First, adding vorinostat to standard chemotherapy has received positive outcomes in treating multiple myeloma, AML, MDS, and NSLCL. The phase III VANTAGE 088 trial showed that the combination of vorinostat and bortezomib for relapsed or refractory multiple myeloma prolonged PFS relative to bortezomib plus placebo (median PFS: 7.63 vs. 6.83 months; HR, 0.77; 95%CI: 0.64, 0.94; *p* = 0.01).^558^ The ORR in the phase II trial of vorinostat with idarubicin and cytarabine for newly diagnosed AML or MDS reached 85%.^559^ Vorinostat enhanced the efficacy of carboplatin plus paclitaxel in first‐line therapy of advanced NSLCL in confirmed response rate (34 vs. 12.5%).^560^ Second, vorinostat improves sensitivity to radiotherapy. In a phase I trial (NCT00455351) for gastrointestinal carcinoma, vorinostat combined with pelvic palliative radiotherapy was safe.^561^ As shown in a phase II trial (NCT02035137), the response rate of vorinostat plus ^131^I‐metaiodobenzylguanidine for patients with relapsed or refractory neuroblastoma was 32% and the value was 14% in the ^131^I‐metaiodobenzylguanidine arm.^562^ Third, HDAC inhibitor combined with ICI is a trend. The studies of pembrolizumab with vorinostat in phase I/Ib study for advanced renal or urothelial cell carcinoma and in a phase II study for progressive advanced mucosal cancer are ongoing (NCT02619253 and NCT04357873). In addition, the combination of HDAC inhibitor and targeted agent of other mechanisms is investigated. Phase I/II trial (NCT01266031) of bevacizumab versus bevacizumab plus vorinostat in adults with recurrent glioblastoma has been completed. The efficacy of DNMT inhibitor plus HDAC inhibitor is not certain. A phase I/II trial (NCT00387465) of combining epigenetic therapy with azacitidine and entinostat resulted in objective and durable responses in patients with heavily pretreated NSCLC.^563^ However, two phase II trials (NCT00313586 and NCT01522976) showed that adding entinostat to azacitidine did not increase clinical response for patients with MDS or AML with myelodysplasia‐related changes. Further studies are needed to determine the feasibility of combination therapy.^564^, ^565^

Many HDAC inhibitors are underdeveloped, including, but not limited to, pracinostat, quisinostat, dacinostat, AR‐42, mocetinostat, and citarinostat. Pracinostat is a pan‐HDAC inhibitor and has been evaluated in the phase II clinical stage for various of solid and hematological tumors, including MDS, AML, prostate cancer, and sarcoma (NCT01993641, NCT01912274, NCT01075308, and NCT01112384).^566^, ^567^, ^568^ Dacinostat and quisinostat belong to the second‐generation and pan‐HDAC inhibitors for class I, II, and IV HDACs.^568^, ^569^ Preclinical evaluation of dacinostat verified an improved efficacy compared with other HDAC inhibitors. Another pan‐HDAC inhibitor, quisinostat, proved its safety and tolerability in phase I trial (NCT00677105) for solid tumors and showed efficacy and safety in patients with previously treated cutaneous T‐cell lymphoma in a phase II trial (NCT01486277).^569^, ^570^, ^571^ AR‐42 is also a pan‐HDAC inhibitor and has been investigated in phase I stage for multiple myeloma, T‐ and B‐cell lymphomas, AML, and solid tumors (NCT01129193 and NCT01798901).^572^, ^573^ Mocetinostat is a class I/IV HDAC inhibitor in the clinical phase II stage.^574^ Citarinostat is a selective HDAC6 inhibitor, and a phase I trial for smoldering multiple myeloma is ongoing (NCT02886065).^575^

### 4.4 XPO1 inhibitors

The nuclear export receptor XPO1, also known as CRM1, with a pleiotropic role in transporting a plethora of proteins and RNA species, including rRNAs, snRNAs, mRNA, microRNAs, and tRNAs, is in charge of the nuclear export of tumor suppressor protein and growth regulators from the nucleus to the cytoplasm.^576^ XPO1 functions together with RAN GTPase, which provides the energy for transport and ensures the directionality of nuclear export. XPO1 is involved in the export of nearly 220 proteins. Among these proteins, several tumor suppressors, including p53, BRCA1/2, and p27, have been extensively studied.^577^ It is overexpressed in multiple cancers, including pancreatic, gastric, prostate, and CRC, and such overexpression is associated with disease progression, treatment resistance, and inferior PFS and/or OS.^578^ Overexpression of XPO1 in various cancers can lead to decreased tumor suppressors and aberrant cytoplasmic localization. Nuclear export blockade of tumor suppressor proteins has been postulated as the primary mechanism of action for XPO1 inhibitors.^579^

The XPO1 inhibitor selinexor covalently and reversibly interacts with Cys528 in the nuclear export signal‐binding pocket of XPO1. Therefore, it restores tumor suppressor function and eventually causes tumor cell apoptosis.^580^ Selinexor was initially proved to be of safety and preliminary antitumor activity in three critical phase I trials for advanced solid tumors, bone or STS, and relapsed or refractory acute leukemia (NCT01607905, NCT01896505, and NCT02212561). The most common adverse events were nausea, vomiting, anorexia, and fatigue. Grade 3–4 toxicities were fatigue, thrombocytopenia, anemia, lymphopenia, and leukopenia.^580^, ^581^, ^582^ Subsequently, many large clinical trials have been launched, and some of their results have been published. In 2019, the phase II STORM trial of selinexor in combination with dexamethasone for patients with triple‐class refractory multiple myeloma showed that a PR or better was observed in 26% of patients with a median DOR of 4.4 months.^583^ Another phase III trial for multiple myeloma was the BOSTON trial. This trial recruited patients with previously treated multiple myeloma to receive once‐per‐week selinexor, bortezomib, and dexamethasone or twice‐per‐week bortezomib and dexamethasone. Median PFS was 13.93 months with selinexor, bortezomib, and dexamethasone and 9.46 months with bortezomib and dexamethasone (HR, 0.70; 95%CI: 0.53, 0.93, *p* = 0.0075). Grade 3–4 adverse events, including thrombocytopenia, fatigue, and anemia, were higher in the three‐drug combination.^584^ Based on the outcomes of STORM and BOSTON trials, the US FDA granted selinexor for the treatment of multiple myeloma. Besides, monotherapy with selinexor is licensed in patients with relapsed or refractory diffuse large B‐cell lymphoma based on the phase II SADAL trial. In this study, single‐agent selinexor improved OS compared with historical data, with an ORR of 28%.^585^, ^586^ Phase III study (NCT04442022) for relapsed or refractory diffuse large B‐cell lymphoma is ongoing to evaluate the value of adding selinexor to R‐GDP.

Clinical trials of selinexor for dedifferentiated liposarcoma, recurrent glioblastoma, NHL, and AML also received positive results. Among them, the studies of dedifferentiated liposarcoma and recurrent glioblastoma have advanced to clinical phase II and/or phase III.^587^, ^588^, ^589^ Notably, in the recent published phase II/III trial SEAL, patients with heavily treated dedifferentiated liposarcoma were assigned to receive selinexor or placebo. PFS with selinexor was longer than that with placebo (median PFS: 2.8 vs. 2.1 months; HR, 0.70; 95%CI: 0.52, 0.95; one‐sided *p* = 0.011). Besides, the absence of CALB1 predicted a longer PFS with selinexor compared with placebo (median PFS: 6.9 vs. 2.2 months; HR, 0.19; *p* = 0.001). Therefore, the expression of CALB1 may be used as a biomarker to choose suitable patients with dedifferentiated liposarcoma for the treatment of selinexor.^588^ Besides, other predictive biomarkers have been investigated in different situations. Cytogenetically normal AML is characterized by mutated NPM1, the protein product of which results in abnormal cytoplasmic localization and hinders differentiation of AML cells. XPO1 inhibition relocalizes it to the nucleus and promotes the differentiation of AML cells. Therefore, XPO1 inhibitors may be effective against cytogenetically normal AML with NPM1 mutations.^590^ In addition, overexpression of XPO1 or mutant (E571K or E571G) XPO1 can drive leukemogenesis and can be obstructed by the XPO1 inhibitor selinexor.^591^ On the contrary, expression of mutant‐p53 in diffuse large B‐cell lymphoma confers resistance to selinexor treatment.^592^

Eltanexor and verdinexor also belong to XPO1 inhibitors, the binding mode of which is similar to that of selinexor.^579^ Etanexor is an investigational XPO1 inhibitor with low CNS penetrance and an acceptable tolerability profile, which yields antitumor activity in preclinical models of human multiple myeloma, ALL, AML, and gastric cancer.^593^, ^594^, ^595^, ^596^ A phase I/II study (NCT02649790) of the safety, tolerability, and efficacy of eltanexor in relapsed or refractory cancer is ongoing. MDS is one of the tumor types in the ongoing trial. The preliminary results were published in August 2022, showing that single‐agent etanexor met the presetting goals.^594^ Verdinexor is another XPO1 inhibitor and shows antitumor efficacy against esophageal carcinoma and neuroblastoma in xenograft models.^597^, ^598^ Phase I trial (NCT02431364) is designed to evaluate the safety and tolerability of etanexor in healthy adults, but it has been terminated.

### 4.5 CXCR4 inhibitors

C‐X‐C‐motif chemokine receptor 4 (CXCR4) is a G‐protein‐coupled receptor involved in a number of physiological processes in the hematopoietic and immune systems and also has important roles in promoting tumor cell proliferation, metastasis, and angiogenesis.^599^, ^600^ CXCR4 is found to be a prognostic marker in many different cancers, including leukemia, breast, lung, prostate, ovarian and CRC, where the stromal cell‐derived factor‐1 (SDF‐1)/CXCR4 axis (also called CXCL12/CXCR4 axis) initiates divergent signaling pathways, including PI3K/AKT, MAPK, and JAK/STAT signals, which can result in a variety of responses such as chemotaxis, cell survival and/or proliferation, increase in intracellular calcium, and gene transcription.^601^, ^602^ In addition, the organs and tissues that possess high levels of SDF‐1, such as liver, lung, bone marrow, and lymph nodes, attract the migration of CXCR4‐expressing cancer cells.^603^ CXCR4 is a promising target for imaging and therapy of both hematologic and solid tumors.^604^

To date, none of the CXCR4 inhibitors are approved directly to target tumors. The CXCR4 inhibitor plerixafor is approved for patients with NHL or multiple myeloma to mobilize hematopoietic stem cells into the peripheral blood for autologous transplantation.^605^, ^606^ However, it has also been investigated in several tumor types. The CXCL12/CXCR4 axis can transactivate HER2 and promote intraosseous tumor growth in prostate cancer. Inhibition of the CXCL12/CXCR4 axis through the CXCR4 inhibitor plerixafor could abrogate the initial establishment of tumor growth in vivo. In other words, plerixafor can prevent bone metastasis in prostate cancer.^607^ Besides, adding plerixafor to radio‐chemotherapy for cervical cancer improved primary tumor response and reduced lymph node metastases with no increase in toxicity in xenograft models.^608^ In addition, the SDF‐1/CXCR4 axis may mediate resistance to VEGFR inhibition in recurrent high‐grade glioma. Preclinical data showed plerixafor could inhibit glioma progression after anti‐VEGF pathway inhibition. Other studies demonstrated that SDF‐1/CXCR4 was responsible for postirradiation tumor revascularization in glioblastoma.^609^, ^610^ To date, the phase I trial of plerixafor and bevacizumab showed the combination regime was well tolerated in high‐grade glioma.^610^ Phase I/II of infusing plerixafor to standard chemoradiation improved local control of tumor recurrences in patients with newly diagnosed glioblastoma.^609^ A clinical study of plerixafor is ongoing for pancreatic cancer (NCT04177810).

There are many other small molecule CXCR4 inhibitors for cancers in development, namely small peptide molecule and small nonpeptide molecule inhibitors.^611^ LY2510924 is one of the peptide molecule inhibitors. It showed preclinical antitumor activity in multiple cancer types and proved safe in phase I trials (NCT02652871,).^612^ However, two phase II studies (NCT01439568 and NCT01391130) for SCLC and RCC failed to deliver the additional role with LY2510924.^613^, ^614^ Mavorixafor belongs to a nonpeptide molecule inhibitor and is a selective, allosteric CXCR4 inhibitor with promising anticancer properties. Phase Ib trial (NCT02923531) of the combination of mavorixafor with nivolumab demonstrated potential antitumor activity and a manageable safety profile in patients with advanced RCC.^615^ Phase I study (NCT04274738) mavorixafor in combination with ibrutinib in patients with WM harboring mutations in MYD88 and CXCR4 is ongoing. Some CXCR4 inhibitors are in preclinical stages, even in the fundamental experiments, such as WZ811 and AMD3465.^616^, ^617^

### 4.6 RAS inhibitors

The principal upstream factor of the MAPK pathway is RAS protein, which is a small, membrane‐bound guanine nucleotide‐binding GTPase and concludes four members, including HRAS, NRAS, KRAS4A, and KRAS4B. The KRAS4A and KRAS4B are two isoforms of KRAS.^279^ About 19% of cancer patients have a RAS mutation, KRAS mutations accounting for the majority of RAS gene mutations (75%), followed by NRAS mutations (17%) and HRAS mutations (7%). 70% of RAS mutations are G12D, G12V, G12C, G13D, and Q61R.^618^ KRAS G12C mutation happened in 13.8% of NSCLC.^619^

Once, RAS protein was thought to be untargetable because of lacking binding pockets on its protein surface.^280^, ^620^ Luckily, the KRAS G12C mutant provides a cysteine residue for designing covalent inhibitors. Sotorasib is a selective and irreversible inhibitor of the KRAS G12C and showed antitumor activity against advanced solid tumors harboring the KRAS G12C mutation.^13^ This agent obtained approval by the US FDA in 2021 for patients with KRAS G12C‐mutated NSCLC who have already received an ICI and/or platinum‐based chemotherapy. The phase II CodeBreak 100 trial showed that the ORR was 37.1% and the median DOR was 11.1 months without new safety signals.^621^, ^622^ Clinical trials involving neoadjuvant therapy and first‐line therapy with sotorasib, and combination therapy with sotorasib and other agents are underway (NCT05400577, NCT04933695, and NCT05074810).

There are other RAS inhibitors or agents of other mechanisms under development for targeting RAS mutant tumors. Adagrasib is the most promising agent. It is also a selective and irreversible KRAS G12C inhibitor. In the phase II study (NCT03785249) of this agent for patients with KRAS G12C mutant NSCLC previously treated with PD‐1/PD‐L1 antibody and platinum‐based chemotherapy, the ORR reached 42.9% and the median DOR was 8.5 months. Besides, the intracranial confirmed ORR was 33.3% and the safety profile was acceptable.^623^ Phase III study (NCT04685135) of adagrasib for patients with KRAS G12C mutant NSCLC is ongoing. JDQ443 is a selective covalent inhibitor of KRAS G12C and forms novel interactions with the switch II pocket.^624^ It showed potent antiproliferative activity in KRAS G12C‐mutated and KRAS G12C/H95 double mutated cell lines and is in clinical trials for KRAS G12C‐mutated tumors (NCT04699188 and NCT05132075). Prenylation of mutant RAS is the primary activator of downstream signaling. The predominant form of prenylation is farnesylation, which is unique in HRAS mutations. Tipifarnib is a highly potent and selective farnesyltransferase inhibitor that disrupts HRAS function. Phase II trial of this agent for head and neck squamous cell carcinoma showed an ORR of 55% in patients with high mutant HRAS variant allele frequency. Median PFS was 5.6 months and median OS was 15.4 months. The most frequent adverse events included anemia and lymphopenia (NCT02383927).^625^ In addition, tipifarnib also resulted in modest clinical activity in HRAS‐mutant salivary gland cancer and urothelial carcinoma harboring HRAS mutations.^626^, ^627^

### 4.7 mTOR inhibitors

mTOR, a classical downstream effector of the PI3K pathway, is usually assembled in two complexes, mTOR complex 1 (mTORC1) and mTORC2, with different regulatory mechanisms.^322^ It is hyperactivated in various solid tumors. Therefore, targeting mTOR in cancer treatment has become a promising strategy.^328^, ^370^

Rapamycin, also known as sirolimus, and its analogs (temsirolimus and everolimus) constitute the first generation of mTOR inhibitors, which bind to FKBP12 and preferentially inhibit mTORC1.^328^ Both temsirolimus and everolimus have been approved to treat RCC. Temsirolimus improved OS (median OS: 10.9 vs. 7.3 months; HR, 0.73; 95%CI: 0.58, 0.92; *p* = 0.0078) and PFS (median PFS: 5.5 vs. 3.1 months; HR, 0.66; 95%CI: 0.53, 0.81; *p* = 0.0001) compared with interferon in previously untreated RCC. Everolimus was superior to placebo for PFS in metastatic RCC after treatment with sunitinib, sorafenib, or both.^628^, ^629^ Besides, the addition of everolimus to exemestane can overcome resistance due to constitutive activation of the PI3K/AKT/mTOR pathway after prior therapy with letrozole or anastrozole in HR‐positive, HER2‐negative breast cancer.^630^ Due to low bioavailability and patent expiration, sirolimus in cancer treatment was hardly seen.^328^ While in 2021, the sirolimus protein‐bound particle was approved for malignant perivascular epithelioid cell tumor based on the phase II AMPECT trial showing an ORR of 39%.^631^ But both the on‐target immunosuppressive roles and resistance caused by subsequent phosphorylation of AKT ser473 by mTORC2 restrict the use of rapamycin and its analogs. mTOR kinase inhibitors are under development, which can simultaneously suppress the activity of both mTORC1 and mTORC2 and have been discussed above.^328^, ^370^

### 4.8 SMO inhibitors

Hedgehog (Hh) signaling plays a pivotal role in embryonic development, tissue homeostasis, regeneration, and stem maintenance in adults. In the absence of Hh ligands, the transmembrane Patched receptor (PTCH) antagonize the activation of smoothened (SMO). Upon Hh ligand binding, the PTCH receptor is exported from the cilium allowing SMO to enter and to activate downstream elements of the pathway. SMO activation promotes the dissociation of glioma‐associated oncogene (GLI) transcriptional factors GLI1, GLI2, and GLI3, from their negative regulator SUFU, and subsequently induce nuclear translocation. Its deregulation in its regulators such as PTCH, SMO, GLI1, and GLI2 causes many different tumors, including basal cell carcinoma (BCC), AML, and several solid cancers.^632^, ^633^ The SMO protein represents a bottleneck in the canonical Hh signal transduction system, and its genetic deletion or pharmacological blockade is associated with a complete abrogation of ligand‐induced target gene expression. A great deal of work has been devoted to the development of SMO inhibitors, which is hoped to be able to block the Hh signaling at upstream level.

The US FDA accepted three SMO antagonists (vismodegib, sonidegib, and glasdegib), to treat BCC or AML.^634^, ^635^, ^636^, ^637^, ^638^ Specifically, Hp signaling is aberrantly activated in around 95% of BCC. Data showed an ORR of 43% in locally advanced BCC and 30% in metastatic BCC in phase II study (NCT00833417) of vismodegib. However, adverse effects often lead to drug discontinuation. Serious adverse events were reported in 25% of patients, including seven deaths.^634^, ^635^ The phase II BOLT study showed that 200 mg sonidegib can also be used in locally advanced or metastatic BCC with an ORR of 43%.^635^ In combination with low‐dose cytarabine, glasdegib was capable of bringing an OS of 8.3 months and an ORR of 18.2% to patients with newly diagnosed AML in adults ≥75 years or with comorbidities that preclude use of intensive induction chemotherapy. Therefore, the US FDA approved glasdegib in combination with low‐dose cytarabine for the treatment of AML.^636^, ^637^, ^638^

While serious adverse events and drug resistance hinder their use in the clinic. Many efforts have been paid to solve these problems.^639^, ^640^ With the high frequency of serious adverse events in SMO inhibitors, researchers tried to find ways to address this problem. First, discontinuation of SMO inhibitors after CR of locally advanced BCC and rechallenging when relapse after discontinuation may be a good way. The data showed the median RFS of discontinuation of vismodegib was 18.4 months, and 85% of relapsed patients could still respond to vismodegib rechallenge. Second, intermittent use of medication ensures efficacy and reduces side effects.^641^ The phase II MIKIE trial showed that two long‐term intermittent vismodegib dosing regimens for BCC showed good activity and largely reduced the percentages of side effects.^642^ The SMO inhibitors’ resistance is mainly from three aspects: drug‐resistance mutations, bypassing signaling activation, and SMO‐independent Hh signaling activation.^640^ The secondary or acquired resistance of the SMO inhibitors in BCC is relatively rare than EGFR inhibitors in NSCLC. SMO‐D473H and SMO‐A459V mutations showed a reduction in sensitivity to vismodegib.^643^ Moreover, the preclinical study found a slow‐cycling tumor population characterized by LGR5 expression and active Wnt signaling was responsible for resistance to vismodegib in treating BCC. Therefore, the synergy between Wnt and smoothened inhibitors is a strategy for overcoming tumor relapse.^644^ Another preclinical trial showed sonic Hh and PI3K pathways were activated in PTEN‐deficient glioblastoma, simultaneously targeting both pathways resulted in markedly growth reduction of PTEN‐deficient glioblastomas.^645^ In addition to the canonical signaling cascade, SMO‐independent activation of GLI cross‐talks with pathways such as MAPK, PI3K/AKT, and NF‐κB and mediates the resistance to the SMO inhibitors. Under this condition, GLI inhibitors can be used for treating cancers.^633^

The approved SMO inhibitors are being tested in other tumor types, and novel SMO inhibitors are developing. Phase I study (NCT01125800) of sonidegib in pediatric brain and solid tumors demonstrated that sonidegib was well tolerated.^646^ Phase II study in children and adults with relapsed medulloblastoma showed that the treatment of sonidegib was effective, with five of the 10 medulloblastomas with activated Hh pathway demonstrating a CR or PR.^646^ However, the phase II studies (NCT00939484 and NCT01239316) of vismodegib for medulloblastoma showed that not all patients responded to vismodegib, indicating the necessity to identify target populations that will genuinely benefit.^647^ Besides, the phase I/II trial (NCT01064622) for pancreatic cancers failed to show any clinical benefit if adding vismodegib to gemcitabine in an unselected cohort.^648^ A phase III study (NCT03416179) evaluating intensive chemotherapy with or without glasdegib or azacitidine with or without glasdegib for patients with untreated AML has been finished. However, the results have not been published. Two phase III studies (NCT04168502 and NCT04842604) of glasdegib for hematological tumors are ongoing. Several new SMO inhibitors exist, such as taladegib, PF‐5274857, Sant‐1, and MRT‐83. Among them, taladegib is in the clinical stage. Phase I trial of this agent for locally advanced or metastatic BCC showed that responses were observed in patients previously treated with Hh therapy (11 out of 31) and in Hh treatment‐naïve (11 out of 16) patients.^649^ Phase II studies of this agent on various types of tumors, are ongoing (NCT05199584). At the same time, the other SMO inhibitors are still in the preclinical stage.

### 4.9 BCL‐2 inhibitors

Anticancer therapies mainly involve viral and cellular oncogenes, cellular proliferation, and transformation. However, programmed cell death was deemed a logical and realistic therapeutic strategy until the discovery of apoptosis after tumor cells exposure to glucocorticoids, cytotoxic agents, or radiation.^650^ The BCL‐2 family contains two subgroups with opposite functions: proapoptotic and antiapoptotic members. BAX, BAK1, BIM, BID, and BBC3 belong to proapoptotic molecules. On the contrary, antiapoptotic molecules conclude BCL‐2, BCL‐XL, BCL‐W, BCL‐2‐A1, and MCL1.^651^ Prosurvival and proapoptotic proteins synergistically balance cellular apoptosis and determine whether apoptosis occurs. Once apoptosis happens, cytochrome C releases from the mitochondria and thereby triggers the activation of proteins of the caspase family, which proteins from the IAP family can negatively regulate.^652^ However, evasion of apoptosis is a hallmark of cancer that arises when this balance is tipped in favor of survival. Many antiapoptotic proteins associated with tumor cell survival and resistance to chemoradiotherapy, such as BCL‐2, BCL‐ XL, MCL1, or the IAP proteins, are overexpressed in human tumors. Therefore, many therapeutic agents have been designed to block these proteins.^653^

To date, only the BCL‐2 inhibitor venetoclax has been approved for treating hematological tumors. Venetoclax belongs to BH3 mimetics, which are small molecules that mimic the binding of BH3‐only proteins to antiapoptotic BCL‐2 proteins and therefore block antiapoptotic function. BH3‐only proteins are a subclass of proapoptotic BCL‐2 proteins that contain only one BCL‐2 homology domain, which can bind to the groove formed by four BCL‐2 homology domains of antiapoptotic members of the BCL‐2 family. The BCL‐2 inhibitors are designed to imitate the structure of BH3‐only proteins.^650^ Venetoclax monotherapy was permitted for the treatment of adult patients with CLL/SLL based on one phase I trial (NCT01328626) and two phase II trials (NCT02141282 and NCT01889186), which demonstrated the efficacy and safety profile of venetoclax in patients with previously untreated CLL harboring 17p deletion and relapsed or refractory CLL/SLL, including those with poor prognostic features or progressing on BTK inhibitors. The ORR ranged from 65 to 79%. The pooled analysis of four early‐phase trials showed that the estimated median PFS, DOR, and TTP were 30.2, 38.4, and 36.9 months, respectively. Serious adverse events occurred in half of the patients, including neutropenia, infection, anemia, and thrombocytopenia. Another adverse event, tumor lysis syndrome, should be avoided by controlling the drug dose.^652^, ^654^, ^655^, ^656^ Subsequent trials of venetoclax combinations further expanded the benefits of venetoclax in the setting of both refractory and untreated CLL/SLL. The phase III CLL14 trial showed that patients with previously untreated CLL and coexisting conditions received longer PFS with venetoclax and obinutuzumab than with chlorambucil and obinutuzumab.^657^ In another phase III MURANO trial, patients with relapsed or refractory CLL were randomly assigned to receive venetoclax plus rituximab or bendamustine plus rituximab. The results showed that venetoclax plus rituximab resulted in significantly higher rates of PFS than bendamustine plus rituximab.^658^ The other indication of venetoclax is for patients with newly diagnosed AML. Venetoclax combined with hypomethylating drugs or chemotherapy (azacytidine, decitabine, or low‐dose cytarabine) was demonstrated to be superior to the chemical drug alone when treating patients with newly diagnosed AML whose disease was ineligible for intensive chemotherapy. The ORR of combining venetoclax with hypomethylating agent therapy (decitabine or azacitidine) was 60% in a phase Ib trial (NCT02203773).^659^ The phase III VIALE‐A trial for previously untreated AML showed OS was significantly longer in patients treated with azacitidine plus venetoclax than those with azacitidine plus placebo (median OS: 14.7 vs. 9.6 months; HR, 0.66; 95%CI: 0.52, 0.85; *p* < 0.001).^529^ Similarly, another phase III trial (NCT03069352) verified the clinical benefits in venetoclax plus low‐dose cytarabine in terms of OS, CR rate, and reduction in risk of death compared with placebo plus low‐dose cytarabine.^660^

Expanding the indications of venetoclax and achieving durable and deep response through combination regimes are two developmental directions for venetoclax. Many tumor types, especially for other hematological tumors are under investigation, such as WM, ALL, and multiple myeloma. Venetoclax was tested in patients with previously treated WM in a phase II study (NCT02677324), which found venetoclax was safe and high active, even in patients progressing on BTK inhibitors.^661^ Single‐agent venetoclax was active in relapsed or refractory multiple myeloma with *t* (11;14) translocation. Phase I study (NCT03314181) combining venetoclax with CD38 antibody daratumumab and dexamethasone demonstrated a high rate of deep and durable responses in patients with relapsed or refractory multiple myeloma, especially for patients with relapsed or refractory multiple myeloma those with *t* (11;14).^662^ Investigating novel combined therapy for the approved indications is another measure. For example, combination of venetoclax with BTK inhibitor ibrutinib or acalabrutinib with or without chemoimmunotherapy with obinutuzumab as frontline treatment for CLL has received positive outcomes in several phase II trials (NCT02910583, NCT03580928, and NCT02756897).^429^, ^663^, ^664^ This combination regime is under investigation in the phase III trial (NCT03836261).

Several resistant mechanisms to venetoclax have been described, including alterations of other BCL‐2 proteins’ expression, mutations in the BCL‐2 protein, and modifications in mitochondrial function. For example, increased expression of BCL‐XL is associated with resistance to venetoclax in MCL. Mutations in the BCL‐2 protein, such as G101V and F104L, could largely reduce the BCL‐2 binding affinity for venetoclax compared with wild‐type BCL‐2. Combinatorial strategies may conquer the resistance.^650^ As mentioned above, agents targeting BCL‐XL, MCL1, or the IAP proteins are also under investigation, such as BCL‐XL inhibitors ABBV‐155, WEHI‐539, and A‐1155463, MCL1 inhibitors AMG176, MIK665, and AZD5991, and IAP inhibitors and SMAC mimetics LCL161 and birinapant. Moreover, there are also dual BCL‐2/BCL‐XL inhibitors, including navitoclax, APG‐1252, and AZD4320.^529^ Take navitoclax as an example. It was initially proved to be safe and effective as a single agent in several phase I studies (NCT00406809 and NCT00481091) for hematological or solid tumors.^665^, ^666^ Another phase I study (NCT03181126) utilized venetoclax with low‐dose navitoclax and chemotherapy to treat patients with relapsed/refractory ALL or lymphoblastic lymphoma. The results showed the combination regimes were well tolerated and had promising efficacy.^667^ Phase II study (NCT03222609) evaluated the addition of navitoclax to ongoing ruxolitinib for patients with myelofibrosis with progression or suboptimal response.^668^ Two phase III studies (NCT04472598 and NCT04468984) are assessing first‐line or post‐line therapy of navitoclax plus ruxolitinib for patients with myelofibrosis.

Selective small molecule inhibitors are often used in patients with the presence or absence of specific predictive molecular alteration detected from tumor tissue or body fluid sampling such as blood.^669^, ^670^ With the development of NGS, it is much easier and more convenient to unlock alterations that exist in genes.^671^, ^672^ However, not all selective small molecule inhibitors require individualized patient selection due to alteration of a particular gene or hyperactivation of a kind of signaling pathway that existed in nearly all cases with a specific tumor.^10^ For example, almost all patients with CLL and AML are characterized by overexpression of BCL‐2, so additional tests are not required when patients use BCL‐2 inhibitor venetoclax.^529^, ^673^

## 5 MULTIKINASE SMALL MOLECULE INHIBITORS

As mentioned above, multikinase inhibitors have a wide range of targets. They block the VEGF/VEGFR signaling pathway while also inhibiting other pathways to exert antiangiogenic and antiproliferative effects.^20^, ^21^ Multikinase inhibitors have various indications, and their development is mainly empirical. None of these drugs make use of a biomarker to identify patients that respond.^10^ To date, there are nine multikinase inhibitors approved by the US FDA, namely sorafenib, sunitinib, pazopanib, vandetanib, axitinib, regorafenib, cabozantinib, lenvatinib, and tivozanib. Clear cell RCC, HCC, and thyroid cancer are the most studied in the clinic^21^, ^674^, ^675^ (Table 3).

**TABLE 3:** Summary of approved multikinase small molecule inhibitors

Class	Drug name	Company	First approval	Targets	Protein substrate	Administration pathway	Indications
VEGFR multikinase	Sorafenib (Nexavar)	Bayer Healthcare	2005	c‐CRAF, BRAF, mutant BRAF, KIT, FLT‐ 3, RET, RET/PTC, VEGFR1, VEGFR2, VEGFR3, PDGFRβ	Tyrosine	Oral	HCC, RCC, radioiodine‐ refractory DTC
VEGFR multikinase	Sunitinib (Sutent)	CPPI CV (Pfizer)	2006	PDGFRα, PDGFRβ, VEGFR1, VEGFR2, VEGFR3, KIT, FLT3, CSF‐1R, RET	Tyrosine	Oral	GIST, RCC, pNET
VEGFR multikinase	Pazopanib (Votrient)	Novartis	2009	VEGFR1, VEGFR2, VEGFR3, PDGFRα, PDGFRβ, FGFR1, FGFR3, Kit, Itk, Lck, c‐Fms	Tyrosine	Oral	RCC, soft tissue sarcoma
VEGFR multikinase	Vandetanib (Caprelsa)	Genzyme Corp	2011	EGFR, VEGFR, RET, BRK, TIE2, EPH receptor, Src,	Tyrosine	Oral	MTC
VEGFR multikinase	Axitinib (Inlyta)	PF Prism CV	2012	VEGFR1, VEGFR2, VEGFR3	Tyrosine	Oral	RCC
VEGFR multikinase	Regorafenib (Stivarga)	Bayer Healthcare	2012	RET, VEGFR1, VEGFR2, VEGFR3, KIT, PDGFR‐alpha, PDGFR‐beta, FGFR1, FGFR2, TIE2, DDR2, TrkA, Eph2A, RAF‐1, BRAF, BRAF V600E, SAPK2, PTK5, Abl, CSF1R	Tyrosine	Oral	CRC, GIST, HCC
VEGFR multikinase	Cabozantinib (Cabometyx, Comertiq)	Exelixis	2012	MET, VEGFR1, VEGFR2, VEGFR3, AXL, RET, ROS1, TYRO3, MER, KIT, TRKB, FLT‐3, TIE‐2	Tyrosine	Oral	RCC, HCC, radioiodine‐ refractory DTC
VEGFR multikinase	Lenvatinib (Lenvima)	Eisai	2015	VEGFR1, VEGFR2, VEGFR3, FGFR1, FGFR2, FGFR3, FGFR4, PDGFRα, KIT, RET	Tyrosine	Oral	Radioiodine‐ refractory DTC, RCC, HCC, endometrial carcinoma
VEGFR multikinase	Tivozanib (Fotvida)	Aveo Pharms	2021	VEGFR1, VEGFR2, VEGFR3, c‐kit, PDGFRβ	Tyrosine	Oral	RCC

Abbreviations: HCC, hepatocellular carcinoma; RCC, renal cell carcinoma; DTC, differentiated thyroid carcinoma; GIST, gastrointestinal stromal tumor; pNET, pancreatic neuroendocrine tumor; MTC, medullary thyroid cancer; CRC, colorectal cancer. Data sources: https://www.fda.gov/drugs/development‐approval‐process‐drugs/drug‐approvals‐and‐databases.

Clear cell RCC is the most common type of RCC and is rich in vascular networks induced by dysregulation of the VHL/HIF pathway, which provides a scientific rationale for developing VEGFR‐focused multikinase inhibitors (sorafenib, sunitinib, pazopanib, axitinib, cabozantinib, lenvatinib, and tivozanib) in this tumor type.^475^, ^674^, ^676^, ^677^ Sunitinib is the only agent used in the adjuvant treatment setting based on a phase III S‐TRAC trial. Compared with placebo, sunitinib given on a 4‐weeks‐on, 2‐weeks‐off schedule for 1‐year prolonged duration of DFS (median DFS: 6.8 vs. 5.6 years; HR, 0.76; 95%CI: 0.59, 0.98; *p* = 0.03).^678^ The US FDA approved sunitinib and four other combination regimes for patients with treatment‐naïve RCC in the first‐line therapy.^677^ Monotherapy with sunitinib had a statistically significant advantage over interferon alfa in the major endpoint of PFS (median PFS: 11 vs. 5 months; HR, 0.42; 95%CI: 0.32, 0.54; *p* < 0.001).^679^, ^680^ Compared with sunitinib, the four combination regimes (axitinib plus avelumab, axitinib plus pembrolizumab, cabozantinib plus nivolumab, and lenvatinib plus pembrolizumab) not only have advantages in PFS but also in OS.^681^, ^682^, ^683^, ^684^ Adding a PD‐1/PD‐L1 inhibitor to a multikinase inhibitor can broadly promote the prognosis of the patient with RCC.^685^ The median PFS of lenvatinib plus pembrolizumab was the longest, reaching 23.9 months in the phase III CLEAR trial.^684^ In addition to tivozanib, the other six multikinase inhibitors approved for RCC can be used in second‐line therapy. Most of them were used in monotherapy, while lenvatinib plus everolimus is the only approved combination form and also brings the best median PFS duration time reaching 14.6 months.^686^ Monotherapy with tivozanib is indicated for RCC after two or more prior systemic therapies. In phase III TIVO‐3 trial, median PFS was significantly longer with tivozanib than with sorafenib (5.6 vs. 3.9 months; HR, 0.73; 95%CI: 0.56, 0.94; *p* = 0.016). Moreover, the proportion of hypertension with tivozanib (the most common grade 3 or 4 treatment‐related adverse event) was lower than with sorafenib (14 vs. 20%).^687^

Sorafenib was the first agent approved for patients with advanced HCC based on the phase III SHARP trial, which revealed survival benefits with sorafenib versus placebo (median OS: 10.7 vs. 7.9 months; HR, 0.69; 95%CI: 0.55, 0.87; *p* < 0.001).^688^, ^689^ In 2018, lenvatinib was allowed to treat advanced HCC in the frontline. In the phase III REFLECT trial, lenvatinib was noninferior to sorafenib in terms of OS (13.6 vs. 12.3 months; HR, 0.92; 95%CI: 0.79, 1.06). The median PFS and ORR on the lenvatinib arm were also superior to the sorafenib arm.^690^ Besides, regorafenib and cabozantinib were available in patients with HCC after treatment with sorafenib. The efficacy and tolerability of regorafenib were demonstrated by the phase III RESORCE trial. Regorafenib improved OS (median OS: 10.6 vs. 7.8 months) with a HR of 0.63 (95%CI: 0.50, 0.79; one‐sided *p* < 0.0001) compared with placebo.^691^ In the phase III CELESTIAL trial, duration of median OS (10.2 vs. 8.0 months; HR, 0.76; 95%CI: 0.63, 0.92; *p* = 0.005) and median PFS (5.2 vs. 1.9 months; HR, 0.44; 95%CI: 0.36, 0.52; *p* < 0.001) was longer with cabozantinib than with placebo. The ORRs were 4% and less than 1%, respectively (*p* = 0.009).^692^ Median OS durations with these agents were significantly prolonged than the placebo, reaching around 1 year.^691^, ^692^, ^693^, ^694^

In thyroid cancer, sorafenib, lenvatinib, and cabozantinib have received US FDA approval to treat radioiodine‐refractory differential thyroid cancer based on phase III trials (NCT00984282, NCT01321554, and NCT03690388). Median PFS in the sorafenib arm was 10.8 months and the value was 5.8 months in the placebo arm (HR, 0.59; 95%CI: 0.45, 0.76; *p* < 0.0001).^695^ Lenvatinib, as compared with placebo, improved PFS (median PFS: 18.6 vs. 3.6 months; HR, 0.21; 95%CI: 0.14, 0.31; *p* < 0.001) and increased response rate (64.8 vs. 1.5%; *p* < 0.001).^696^ Similarly, cabozantinib significantly prolonged PFS compared with placebo (HR, 0.22; 95%CI: 0.13, 0.36; *p* < 0.0001).^697^ Two multikinase inhibitors, vandetanib and cabozantinib, were on the market for radiographic progression of metastatic MTC.^21^, ^698^ Vandetanib was superior to placebo in terms of PFS (HR, 0.46; 95%CI: 0.31, 0.69; *p* < 0.001) and ORR (*p* < 0.001).^699^ The phase III trial (NCT00704730) showed that the median PFS and response rate were 11.2 months and 28% for cabozantinib versus 4.0 months and 0% for placebo.^700^ In total, all the drugs showed significant improvement in PFS with or without an increase in ORR compared with the placebo. However, none of them demonstrated a significant difference in OS.^696^, ^697^, ^699^, ^700^

Advanced imatinib‐resistant GISTs respond to second‐line sunitinib and third‐line regorafenib, both of which not only inhibit KIT but also PDGFR and/or VEGFR family members.^76^, ^701^, ^702^ Compared with placebo, sunitinib prolonged median time to tumor progression from 6.4 weeks to 27.3 weeks (HR, 0.33; 95%CI: 0.23, 0.47; *p* < 0.0001) and median PFS from 6.0 weeks to 24.1 weeks (HR, 0.33; 95%CI: 0.24, 0.47; *p* < 0.0001).^703^ In the following studies, researchers found that sunitinib preferentially suppresses KIT with secondary resistance mutations affecting the ATP‐binding pocket (exons 13 and 14) and exon 9 mutations. Unlike imatinib, PFS and OS of patients with KIT exon 9 mutations or with a wild‐type genotype were longer than that of those with KIT exon 11 mutations. Median PFS and median OS for patients with primary KIT exon 9 mutations reached 19.4 months and 26.9 months, 19.0 months and 30.5 months for patients with a wild‐type genotype, and 5.1 months and 12.3 months for patients with KIT exon 11 mutations. In addition, PFS and OS of patients with secondary KIT exon 13 or 14 mutations were longer than for those with exon 17 or 18 mutations.^704^ For regorafenib, the phase III GRID trial showed that third‐line therapy with regorafenib largely prolonged median PFS compared with placebo (4.8 vs. 0.9 months; HR,0.27; 95%CI: 0.19, 0.39; *p* < 0.0001) and DCR was 52.6 and 9.1%, respectively. No significant difference in OS between study arms was found, because 84.8% of patients in the control arm crossed over to regorafenib.^705^ A clinical study involving 33 patients showed patients whose tumors harbored a KIT exon 11 mutation had longer median PFS than patients with KIT/PDGFRA wild‐type and non‐SDH‐deficient tumors (13.4 vs. 1.6 months, *p* < 0.0001).^706^ While the definite correlation between kinase genotypes and the biological and clinical activity of regorafenib needs more researches.

Several other factors concerned with multikinase inhibitors deserve attention. First, in addition to the above diseases for multikinase inhibitors, they also have other indications: sunitinib for pNET, pazopanib for STS, regorafenib for metastatic CRC, and lenvatinib for endometrial carcinoma.^707^, ^708^, ^709^, ^710^ Second, the most common toxicities include hypertension, hand‐foot skin reaction, diarrhea, decreased weight and appetite, nausea, and fatigue, mainly attributed to on‐targeted mechanisms.^697^ Corresponding medications can treat these side effects to relieve symptoms, such as antihypertensive drugs for high blood pressure. Side effects can also be alleviated by reducing or discontinuing multikinase inhibitors.^711^ Third, as mentioned above, no markers are established to indicate the efficacy of multikinase inhibitors, especially for the only vascular‐targeted multikinase inhibitors. The resistance mechanisms of multikinase inhibitors are different from that of other small molecular inhibitors. Resistance to VEGFR inhibitors usually does not involve secondary mutation of the target but instead activates bypassing pathways that stimulate angiogenesis. Therefore, combining other mechanisms to synergistically inhibit tumor blood vessels may overcome the resistance of multikinase inhibitors.^220^

Many small molecule inhibitors targeting VEGFR are still in clinical trials and can be divided into selective VEGFR kinase inhibitors, dual mechanism inhibitors, and multikinase inhibitors (Table S3). Among them, multikinase inhibitors are the best developed. Selective VEGFR kinase and dual mechanism inhibitors relatively lag behind.

The development of selective VEGFR kinase inhibitors is limited. Vatalanib and semaxanib belong to selective VEGFR2 kinase inhibitors. Vatalanib mainly targets VEGFR tyrosine kinases in submicromolar range but it also inhibits PDGFR, KIT, and c‐Fms at higher concentrations. The antiangiogenic drug vatalanib added to chemotherapy with FOLFOX4 failed to show advantages in OS and PFS compared with FOLFOX4 in two phase III trials for first‐ and second‐line treatment of CRC (CONFIRM 1 and 2).^712^, ^713^ Similarly, trials of semaxanib for patients with advanced STS or head and neck cancers showed that semaxanib was well tolerated but with limited antitumor activity.^714^, ^715^ Therefore, the development of vatalanib and semaxanib was suspended and predicted limited roles of small molecule inhibitors that only target VEGFR2. Luckily, fruquintinib, a VEGFR1‐3 inhibitor, has been approved in China for NSCLC and CRC. Phase II study (NCT02590965) of third‐ and fourth‐line fruquintinib in patients with advanced nonsquamous NSCLC was superior to placebo in the form of PFS, survival rate, and DCR.^716^ The phase III FRESCO trial demonstrated that third‐line or later therapy with fruquintinib significantly prolonged PFS (median PFS: 3.7 vs. 1.8 months; HR, 0.26; 95%CI: 0.21, 0.34; *p* < 0.001) and OS (median OS: 9.3 vs. 6.6 months; HR, 0.65; 95%CI: 0.51, 0.83; *p* < 0.001) compared with placebo.^717^ Fruquintinib was licensed based on these two clinical trials.

As mentioned above, multikinase inhibitors have made great success in treating cancers. In addition to the US FDA‐approved multikinase inhibitors, anlotinib, apatinib, donafenib, and surufatinib have received approval in China. Anlotinib was licensed for NSCLC and STS. The phase III ALTER 0303 trial showed that anlotinib improved PFS (median PFS: 5.4 vs. 1.4 months; HR, 0.25; 95%CI: 0.19, 0.31, *p* < 0.001) and OS (median OS: 9.6 vs. 6.3 months; HR, 0.68; 95%CI: 0.54, 0.87; *p* = 0.002) in patients with advanced NSCLC progressing after second‐line or further treatment compared with a matched placebo.^718^ A phase II trial (NCT01878448) of this agent showed progression‐free rate at 12 weeks for refractory STS was 68% and ORR was 13%.^719^ Apatinib approved for gastric adenocarcinoma and gastroesophageal junction adenocarcinoma was based on a phase III trial, which demonstrated a longer duration of PFS (median PFS: 2.6 vs. 1.8 months; HR, 0.44; 95%CI: 0.331, 0.595; *p* < 0.001) and OS (median OS: 6.5 vs. 4.7 months; HR, 0.709; 95%CI: 0.537, 0.937; *p* = 0.0156) with apatinib than with a placebo.^720^ Donafenib is a deuterated sorafenib derivative and was allowed for first‐line treatment of HCC, as it exhibited superior OS outcomes (median OS: 12.1 vs. 10.3 months; HR, 0.831; 95%CI: 0.699, 0.988; *p* = 0.245) and fewer adverse events (38 vs. 50%; *p* = 0.0018) versus sorafenib.^721^ The last approved agent, surufatinib, is for neuroendocrine tumor, including extrapancreatic and pNETs based on two phase III trials (SANET‐ep and SANET‐p).^722^, ^723^ In addition, many multikinase inhibitors are in the developmental stage, such as altiratinib, cediranib, dovitinib, lucitanib, ningetinib, nintedanib, and telatinib. Cediranib is under phase III stage for ovarian, fallopian tube, and primary peritoneal cancer (NCT03278717, NCT02502266, and NCT02446600). Phase III studies of this agent for CRC, glioblastoma, biliary tract neoplasm, and NSCLC have been completed (NCT00399035, NCT00384176, NCT00777153, NCT00939848, NCT00795340, and NCT00245154). The primary endpoints were not met in glioblastoma and biliary tract neoplasm.^724^, ^725^ Dovitinib has been tried in many tumor types and has even been submitted an application to the US FDA for third‐line treatment of RCC based on positive results from a phase III study (NCT01223027).^726^ Lucitanib is an inhibitor of VEGFR1‐3, FGFR1‐3, and PDGFRα/β. Previous studies (NCT02053636 and NCT01283945) of lucitanib for breast cancer and solid tumors indicated that FGFR alteration was correlated with the sensitivity of lucitanib.^727^ Phase II/III trial (NCT04254471) of lucitanib in patients with SCLC is recruiting. Ningetinib is also a multikinase inhibitor with potent activity against MET, VEGFR2, and Axl. It has just completed a phase I trial for solid tumors (NCT04577703) and is ready to recruit patients with NSCLC harboring MET exon 14 skipping mutations for phase II study (NCT04992858). Similarly, a phase I trial (NCT03175497) of telatinib for solid tumors has been completed, and patients with gastric cancer and HCC are under phase II stage (NCT04798781) with telatinib. Nintedanib is successfully used in treating idiopathic pulmonary fibrosis and interstitial lung disease, but the indications for cancers are far from reaching.^728^, ^729^ Altiratinib is in preclinical stage. The development of these drugs will give more chances to patients.

The limited efficacy of selective VEGFR inhibitors and side effects companied with multikinase inhibitors urge the research on dual mechanism inhibitors, such as dual VEGFR/MET inhibitors foretinib and golvatinib, dual VEGFR/PDGFR inhibitors linifanib and vorolanib, and dual VEGFR/FGFR inhibitors brivanib and ODM‐203. The trials of the dual VEGFR/MET inhibitors stalled in phase II stage. Foretinib demonstrated activity in patients with advanced papillary RCC, especially in patients with germline MET mutations, and a manageable toxicity profile (NCT00726323).^730^ However, negative results were obtained from trials of triple‐negative breast cancer and gastric cancer (NCT01147484 and NCT00725712).^731^, ^732^ Although golvatinib has completed two phase II clinical studies (NCT01332266 and NCT01271504) for HCC and squamous cell carcinoma of the head and neck, the results have not yet been published. Clinical trials of dual VEGFR/PDGFR inhibitor linifanib are basically not continued because of limited efficacy and increasing toxicity. The only phase III trial (NCT01009593) of linifanib compared efficacy and tolerability of linifanib versus sorafenib in HCC without prior systemic therapy. However, linifanib did not meet predefined superiority and noninferiority OS boundaries.^733^ Vorolanib is a promising dual VEGFR/PDGFR inhibitor and has received preliminary efficacy in solid tumors with acceptable safety profiles (NCT03511222).^734^ Phase II study (NCT04373369) of vorolanib plus atezolizumab for extensive‐stage SCLC is ongoing. Dual inhibition of VEGFR and FGFR is another trend and may be beneficial for FGF/FGFR‐altered tumors. Brivanib received positive results in both phase I and II clinical trials but failed in all phase III clinical trials for HCC, all of which set OS as the primary endpoint, including frontline and second‐line therapies for advanced HCC and adjuvant therapy for unresectable HCC after transcatheter arterial chemoembolization (NCT00355238, NCT00858871, NCT00908752, and NCT00825955).^735^, ^736^, ^737^, ^738^ It is now under stage II trial for cervical cancer (NCT04395612). ODM‐203 is a pan‐VEGFR/FGFR inhibitor and has shown preliminary signs of therapeutic activity in solid tumors with acceptable tolerability (NCT02264418).^739^ More researches are needed to verify the safety and effectiveness of ODM‐203. In general, dual inhibitors can theoretically increase efficacy and reduce side effects, but the practical application remains to be verified.

## 6 CONCLUSIONS AND PERSPECTIVES

The development and approval of small molecule inhibitors have essentially transformed multiple cancers’ clinical care and improved prognosis in many tumors.^124^ Take NSCLC as an example. Chemotherapy was used to be the most popular treatment for locally advanced or metastatic NSCLC. While specific small molecule inhibitors now are widely used for NSCLC with EGFR mutation, ALK or ROS1 rearrangement, MET mutation, which can improve ORRs and PFS and reduce adverse side effects compared with chemotherapy.^740^, ^741^ Despite substantial progress in discovering small molecule inhibitors, many challenges and opportunities remain in the field.

### 6.1 Targets

**FIGURE 3:**
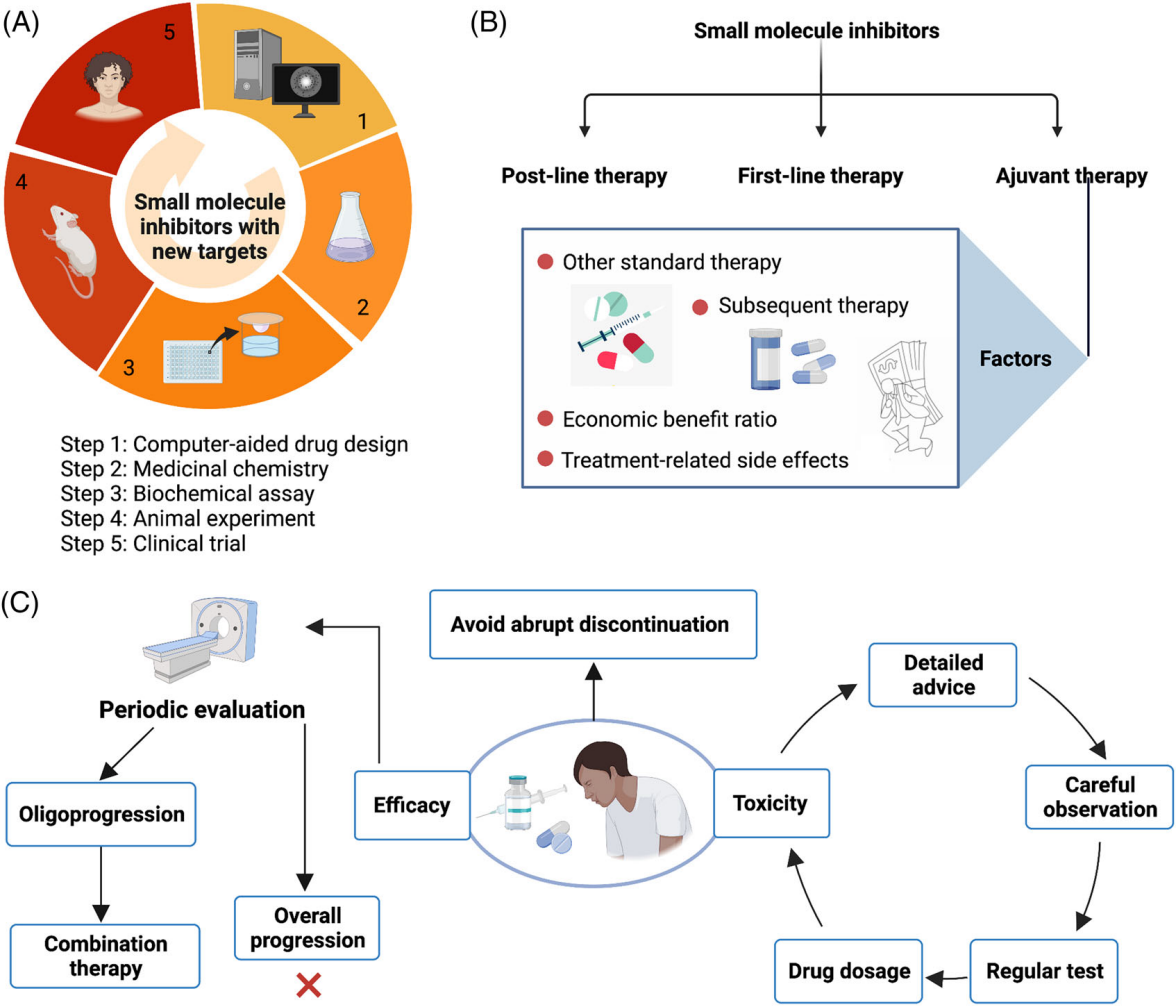
Some of the challenges and future directions of small molecule inhibitors. (A) Approaches to develop small molecule inhibitors with new targets. (B) Factors for adjuvant therapy with small molecule inhibitors. (C) Precautions in the clinical use of small molecule inhibitors. Figure created with BioRender.com

It is reported that at least one alteration in genes can be detected in approximately 40% of cancers.^742^ Although we have made significant progress in developing small molecule inhibitors targeting cancers, minimal gene alterations have successfully translated into clinical use.^743^ Take protein kinase inhibitors as an example, approved drugs have targeted only 5% of protein kinases.^5^ At the same time, a network of proteins contributes to repair DNA damage, only PARP inhibitors of this category have been approved.^9^ New therapeutic targets are to be investigated. With the development of computer‐aided drug design, medicinal chemistry, biochemical assays, animal experiments, and clinical trials, we believe more and more small molecular inhibitors with new targets will be developed to treat cancers.^1^ Actually, small molecule inhibitors targeting the aurora kinases, transcriptional kinases, immuno‐regulatory kinases, and EZH2 are actively pursued by pharmaceutical and academic groups^669^, ^744^ (Figure 3A).

### 6.2 Adjuvant and neoadjuvant therapy

The development of small molecule inhibitors is often initially used for post‐line therapy, gradually transitioning to first‐line treatment, and finally trying to be applied in adjuvant and neoadjuvant settings.^745^, ^746^

To date, several agents have received the permits for patients with early malignancies after surgery, such as imatinib for KIT or PDGFRA mutant GISTS, osimertinib for NSCLC with EGFR exon 19 deletion or exon 21 (L858R) mutations, neratinib for HER2‐positive breast cancer, dabrafenib plus trametinib for melanoma with BRAF V600E or V600K mutations, and sunitinib for RCC.^82^, ^156^, ^294^, ^678^, ^747^ Some of them can bring obvious clinical benefits to patients, like imatinib for patients with KIT or PDGFRA mutations.^82^ But more factors should be considered for patients who have traditional treatment in the adjuvant setting or whose advantages are insignificant.^747^ In NSCLC, adjuvant chemotherapy is still the standard for NSCLC and osimertinib is the most potent EGFR‐TKI for locally advanced or metastatic NSCLC. Subsequent therapies will be complex once patients progress to the early stage after treatment with osimertinib.^748^ Another example is sunitinib for RCC. Patients with RCC were treated for nine cycles (approximately 1 year) after surgery and 5‐year DFS rate only increased from 51.3% with placebo to 59.3% with sunitinib.^678^ In addition, more drugs did not meet the primary endpoints in clinical trials and were not approved for adjuvant therapy. For example, the phase III BRIM8 trial showed that the benefit of DFS in adjuvant vemurafenib monotherapy for 52 weeks was not consistency compared with placebo in patients with resected, BRAFV600 mutation‐positive melanoma.^749^ Taken together, the economic benefit ratio and treatment‐related side effects deserve more consideration in this situation^750^ (Figure 3B).

Though many efforts have been made for the neoadjuvant treatment of cancers with promising outcomes, no targeted drug has yet been approved. Although not yet approved, scientists have not stopped exploring. As mentioned above, numerous small molecule inhibitors have been explored in the field of neoadjuvant therapy, such as MET inhibitor capmatinib for NSCLC, TRK inhibitor larotrectinib for sarcomas, PI3K inhibitor taselisib, CDK4/6 inhibitor palbociclib, and AKT inhibitor MK‐2206 for breast cancer. ^242^, ^357^, ^409^ Another example is the use of neoadjuvant dabrafenib plus trametinib therapy in resectable stage III BRAF V600 mutant melanoma, which led to a high proportion of patients achieving a complete response (46%) and a complete pathological response (49%) without progression in a single‐arm phase II study.^751^ These trials are only in early stage, and these is still a long way from the indications for neoadjuvant therapy.

### 6.3 Efficacy and toxicity

It is crucial to balance the drugs’ efficacy and toxicity. Nearly all the small molecule inhibitors have a chance to be halted in more or less percent of patients because of severe side effects.^6^ In the clinic, detailed advice, careful observation, and regular testing of relevant indicators should be given to patients on medication. Dosage reduction or complete drug discontinuation should be adopted according to adverse reaction grade^5^ (Figure 3C).

### 6.4 Treatment interruption

We should avoid abrupt discontinuation of small molecule inhibitors without any other systematic treatment when the patients encounter the onset of progression. Because of differences in growth kinetics of responsive and resistant tumor subclones, abrupt discontinuation may lead to a disease flare.^1^, ^10^ Topical therapies such as radiotherapy are recommended for oligoprogression without cessation of small molecule inhibitors^752^, ^753^ (Figure 3C). However, small molecule inhibitors are not impossible to interrupt, they just need to be stopped at the right time. Treatment discontinuation of imatinib can be safe in patients with CML who have been in deep molecular remission for at least 2 years.^754^ A series of trials evaluated the safety of treatment discontinuation in nearly 4000 patients, which showed that approximately 50% of individuals experienced long‐term treatment‐free remission (TFR).^755^ What is more, combination ABL1 kinase inhibitors with agents of other mechanisms, such as JAK/STAT inhibitors, BCL‐2 inhibitors, and IFN‐α is demonstrated to achieve better elimination of CML stem cells and deeper molecular response, which in turn offer more possibilities for patients to discontinue therapy.^756^ In addition, advances in technology also help patients with CML achieve TFR. Disease undetectable verified by both conventional and droplet digital PCR (ddPCR) showed only a 10% risk of disease relapse. In comparison, the risk of relapse was 50–65% through ddPCR or conventional RT‐PCR.^757^ Whereas, imatinib discontinuation is not suitable for patients with GISTSs free of progression after 3 years of imatinib. Two‐year PFS rate was higher in the continuation group than in the interruption group (80 vs. 16%; *p* < 0.0001).^758^

### 6.5 Drug resistance and countermeasures

**FIGURE 4:**
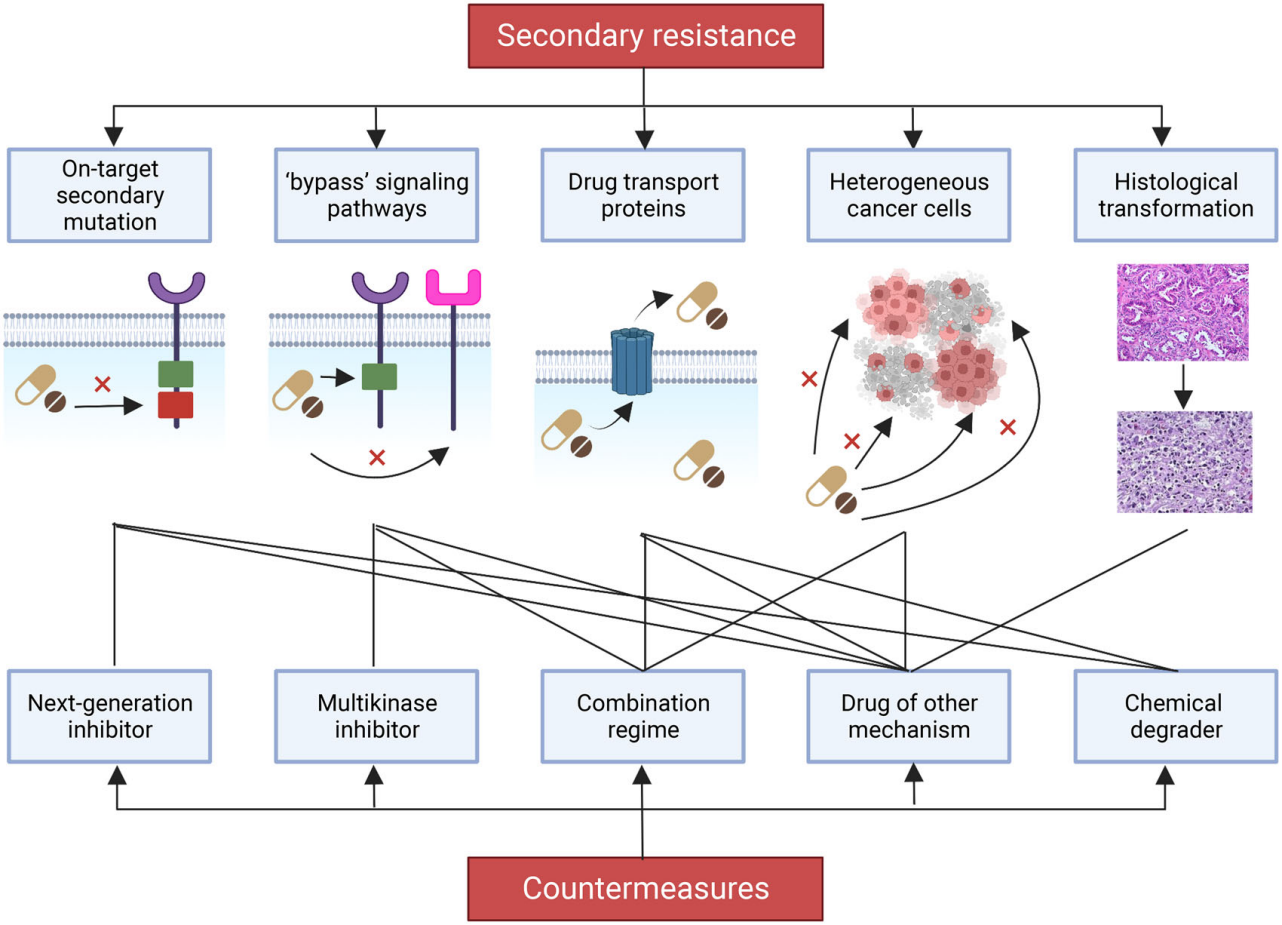
Mechanisms of secondary resistance of small molecule inhibitors and countermeasures. Figure created with BioRender.com

Resistance to small molecule inhibitors can be divided into primary and secondary resistance.^759^ Combination therapies are often needed to tackle primary resistance because of the molecular heterogeneity of tumors.^760^ The small molecular inhibitors are not curative, and the emergence of secondary resistance is a significant barrier to achieve long‐term remission in cancer. The reasons for secondary resistance are relatively complex.^6^ In this part, we focus on secondary resistance and corresponding solutions (Figure 4).

First, on‐target secondary mutations have been studied a lot, which spurs the enthusiasm for finding the next‐generation inhibitors with fewer resistance liabilities, such as ABL TKIs in CML, EGFR and ALK TKIs in lung cancer, and KIT TKIs in GISTs.^75^, ^756^, ^761^ Second, overexpression or amplification of other kinases with similar functions as the drugged kinase can cause the acquisition of “bypass” signaling pathways.^762^, ^763^ Multikinase inhibitors having the ability to target multiple targets can be applied for targeting “bypass” signaling pathways. For example, second‐line sunitinib and third‐line regorafenib are used for imatinib‐resistance GISTs.^76^ The use of a combination regimen is another approach. MET amplification is the second main reason for resistance to first‐generation EGFR TKIs, which may be controlled by dual EGFR/MET inhibition.^764^ One general mechanism is the production of drug transport proteins.^765^ In clinic, we can observe the phenomenon that different sites of tumor cells show different responses to small molecule inhibitors.^766^, ^767^ Next‐generation agents with markedly improved CNS penetration are capable of controlling central lesions. In rare cases, histological transformation also accounts for secondary resistance.^768^ However, some researchers found that other tissue types existed before treatment but became the primary type after medication.^769^ In short, next‐generation inhibitors, combination regimes, or drugs of other mechanisms can deal with secondary resistance under their individual conditions.

In addition to traditional ways to address resistance, new methods which are capable of inducing loss of the total proteins, rather than just loss of their functions, are ideal for overcoming resistance caused by mutation or overexpression.^770^, ^771^ Proteolysis‐targeting chimeras (PROTACs) fall into the class of chemical degraders and are heterobifunctional small molecules containing two ligands linked by a linker. The two ligands recruit and bind an E3 ligase complex and a specific protein of interest, respectively, promoting ubiquitination and subsequent degradation of the target proteins.^772^, ^773^ It is reported that E3 ligase–PROTAC–target ternary complexes induce approximately 200 ePKs degradations. Although none of these kinds of inhibitors are on the market and a small number of them are in the clinical development, it is believed that more PROTACs will enter clinical development in the near future.^774^

### 6.6 Combination therapy

Combination therapy aims to increase and prolong the therapeutic benefits or reversal of primary and acquired resistance through the utility of agents with different mechanisms.^775^ Several strategies can be used to achieve this goal.

First, vertical blockade of one signaling pathway is a strategy to increase the curative effect of small molecule inhibitors.^287^ Both BRAF and MEK are the key molecules in the MAPK pathway. Three combinations of BRAF and MEK inhibitors, cobimetinib plus vemurafenib, dabrafenib plus trametinib, and encorafenib plus binimetinib, have shown their superior to BRAF or MEK inhibitor monotherapy and have been approved by the US FDA for metastatic melanoma with BRAF V600E or V600K mutations.^275^, ^282^

Second, small molecule inhibitors’ horizontal inhibition of two or more pathways is another schedule.^75^ Multikinase inhibitors are a good example, which have been approved for various indications.^76^ Although the combination of different selective small molecule inhibitors that inhibit different signaling pathways have not been approved, many similar efforts have been made.^764^ For example, the combination of ABL1 inhibitor imatinib and MEK inhibitor binimetinib for treatment‐naive adult patients with confirmed advanced GISTs received 69.0% of ORR, which was 20% improvement in the ORR over imatinib alone.^776^ Addition of BCL‐2 inhibitor navitoclax to ongoing ruxolitinib therapy could reverse the resistance for patients with myelofibrosis, whose disease had been progressed or suboptimal response to ruxolitinib monotherapy.^777^ In addition, the combination of antibody drugs and small molecule inhibitors is also an effective way to jointly inhibit multiple signaling pathways. Dual blockade of the EGFR and VEGF pathways in EGFR‐mutated metastatic NSCLC is an example. The trials enrolled patients with metastatic EGFR‐mutant NSCLC to receive bevacizumab (or ramucirumab) plus erlotinib or erlotinib alone and the results showed that the addition of bevacizumab (or ramucirumab) to erlotinib significantly prolonged PFS but failed to prolong survival (median OS: 50.7 vs. 46.2 months; HR, 1.007; 95%CI: 0.681, 1.490; *p* = 0.97).^159^, ^778^, ^779^ Prolonged PFS was also found in the PI3Kα/δ copanlisib plus CD20 antibody rituximab group for indolent NHL, as compared with rituximab alone.^780^ However, not all targets can be used in combination. The combination of DNMT inhibitor and HDAC inhibitor failed to show clinical benefits for patients with MDS and AML compared with monotherapy with HDAC inhibitor.^564^ In other words, simultaneous horizontal inhibition of multiple targets or pathways is not always successful in all endpoints. Whether dual blockade suitable for clinical treatment deserves more consideration, including efficacy, cost‐effectiveness, compliance, and safety.

Combined small molecule inhibitors with ICIs is a prevailing direction.^781^ The PD‐1/PD‐L1 inhibitors are the most commonly used ICIs, restoring the ability of immune cells to destroy tumors. But the ORR of monotherapy with PD‐1/PD‐L1 inhibitors is less than 20%.^782^ On the other hand, small molecule inhibitors have pleiotropic effects on the immune system. VEGFR inhibitors modulate the TME by normalizing blood vessels. PI3K/mTOR inhibitors themselves are crucial for immune activation.^783^ Besides, most small molecule agents have the potential to block oncogenic signaling pathways and modulate PD‐L1 expression. For example, PARP inhibitors and MET inhibitors can upregulate PD‐L1 expression in breast cancer and HCC, respectively.^784^ Therefore, combining ICIs with appropriate small molecule inhibitors may confer a synergistic response in oncogenic addiction cancers. In RCC, four combination regimes (axitinib plus avelumab, axitinib plus pembrolizumab, cabozantinib plus nivolumab, and lenvatinib plus pembrolizumab) represent the best therapeutic effect for local advanced or metastatic RCC.^681^, ^684^, ^685^ Another example is unresectable advanced BRAFV600 mutation‐positive melanoma, the addition of atezolizumab to targeted therapy with vemurafenib and cobimetinib significantly increased PFS (median PFS: 15.1 vs. 10.6 months; HR, 0.78; 95%CI: 0.63, 0.97; *p* = 0.025) and was proved to be safe in phase III IMspire 150 trial.^308^ However, monotherapy with PD‐1/PD‐L1 inhibitors has poor efficacy in NSCLC with EGFR mutations because of a noninflamed TME formed in EGFR‐mutated cancer.^785^ The phase I TATTON trial also showed that osimertinib plus durvalumab was not suggested due to increased interstitial lung disease in patients with EGFR‐mutant NSCLC progression on a prior EGFR‐TKI. Downregulation of PD‐L1 due to EGFR inhibitors may make it better not to use it in combination.^163^ In addition, more experiments and clinical trials are required to determine whether a specific small molecule inhibitor is appropriately combined with a PD‐1/PD‐L1 inhibitor.^775^

Combination with other traditional therapies is also prevalent, such as a CDK4/6 inhibitor plus an aromatase inhibitor for HR‐positive, HER2‐negative breast cancer, BCL‐2 inhibitor plus chemotherapy for AML, HDAC inhibitor plus radiotherapy for neuroblastoma.^397^, ^562^, ^786^ Like combination of small molecule inhibitors with PD‐1/PD‐L1 inhibitors, small molecule inhibitors are not always suitable to combine with chemotherapy. For example, sunitinib plus FOLFIRI was not superior to FOLFIRI alone in median PFS (8.4 vs. 7.8 months; HR, 1.095; 95%CI: 0.892, 1.344; *p* = 0.807) and had a poor safety profile in previously untreated metastatic CRC.^787^ Though gefitinib plus pemetrexed or pemetrexed and carboplatin improved PFS compared with gefitinib alone in patients with untreated advanced NSCLC with EGFR mutations, the phase III IMPRESS trial indicated that the continuation of gefitinib plus chemotherapy was detrimental to OS when compared with placebo plus chemotherapy in patients with EGFR mutation‐positive NSCLC resistant to first‐line gefitinib (median OS: 13.4 vs. 19.5 months; HR, 1.44; 95%CI: 1.07, 1.94; *p* = 0.016), warning that first‐generation EGFR TKIs cannot be continued when chemotherapy is initiated after disease progression.^158^, ^788^ What is more, some inhibitors are mechanistically not expected to cooperate with specific mechanisms of chemotherapy. For example, CDK4/6 inhibitors are inappropriate in combination with DNA‐damaging or antimitotic chemotherapies as the former prevent cell‐cycle entry, thereby disturbing S‐phase‐ or mitosis‐targeting agents.^789^ Taken together, combination small molecule inhibitors with chemotherapies needs rational mechanisms and should be verified by clinical trials.

There are multiple ways to achieve combination therapy, but whether combination therapy is effective still needs to be verified by trials, especially large clinical trials, rather than a blind combination. Although clinical trials of combination therapy are at risk of failure, they are still a potential direction. We should continue researching combination therapies to improve patients’ prognoses.

In summary, small molecule inhibitors bind to a wide range of targets to control the proliferation, survival, apoptosis, differentiation, metabolism, and TME of malignancies, which offer convenience and good curative effect to patients with tumors harboring specific gene alterations or patients with specific histological tumors.^670^, ^760^ Although some problems and obstacles exist in the field, small molecule inhibitors will undoubtedly continue to play an essential role in treating cancers in the following decades.^10^

## AUTHOR CONTRIBUTION

H. S. S. offered direction and guidance of the manuscript. G. H. L. and T. C. drafted the initial manuscript. X. L. M. revised the manuscript. G. H. L. and X. Z. illustrated the figures and tables for the manuscript. All authors approved the final manuscript.

## CONFLICT OF INTEREST

The authors have no conflict of interest to declare.

## ETHICS STATEMENT

No ethical approval was required for this study.

## Supporting information

Supplementary Table S1 Summary of the pivotal candidates of small molecule kinase inhibitors. Data sources: https://clinicaltrials.gov/

Supplementary Table S2 Summary of the pivotal candidates of small molecule non‐kinase inhibitors. Data sources: https://clinicaltrials.gov/

Supplementary Table S3 Summary of the pivotal candidates of small molecule inhibitors targeting VEGFR.Data sources: https://clinicaltrials.gov/

## Data Availability

The data that support the findings of this study are available from the corresponding author upon reasonable request.
